# Synthesis of a heparin-related GlcN–IdoA sulfation-site variable disaccharide library and analysis by Raman and ROA spectroscopy

**DOI:** 10.1016/j.carres.2014.06.026

**Published:** 2014-12-05

**Authors:** Gavin J. Miller, Steen U. Hansen, Marek Baráth, Christian Johannessen, Ewan W. Blanch, Gordon C. Jayson, John M. Gardiner

**Affiliations:** a Manchester Institute of Biotechnology and School of Chemistry, University of Manchester, 131 Princess Street, Manchester M1 7DN, UK; b Manchester Institute of Biotechnology, University of Manchester, 131 Princess Street, Manchester M1 7DN, UK; c Department of Chemistry, University of Antwerp, Groenenborgerlaan 171, B2020 Antwerp, Belgium; d Institute of Cancer Sciences, Christie Hospital and University of Manchester, Wilmslow Road, Manchester M20 4BX, UK

**Keywords:** Disaccharides, Heparin, Sulfated carbohydrates, Raman–ROA, Iduronic aid

## Abstract

Synthesis of an array of differentially sulfated GlcN–IdoA disaccharides, accessible on good scale, directly from l-iduronate components is described. These are specifically directed to provide the sulfation variability at the key most common biologically relevant sulfation-variable l-IdoA O-2 and d-GlcN O-6 and amino sites of this heparin disaccharide. This sulfation-varied matrix has allowed the first evaluation of using Raman/ROA spectroscopy to characterize changes in spectra as a function of both site and level of sulfation with pure, defined heparin-related disaccharide species. This provides analysis of both similarities and differences to digest native heparin and this shows evidence of different types of changes in conformations and conformational freedom as a function of some specific sulfation changes at the disaccharide level. It is anticipated that this data set will open the way for applications to further site-specific sulfated saccharides and demonstrates the capability offered by Raman–ROA towards fingerprinting sulfation in heparin fragments.

## Highlights

• Synthesis of a matrix of sulfation-programmed GlcN–IdoA disaccharides.
• Demonstrates effective synthesis using various GlcN donors and Ido acceptor.
• Four homogeneous sulfation-varied heparin disaccharides used to obtain Raman and Raman ROA data.
• Spectra provide indicative comparisons of signature sulfation bands for differing sulfation levels.
• Data are used to provide comparisons/differences to native heparin spectra.

## Graphical abstract

**Figure:**
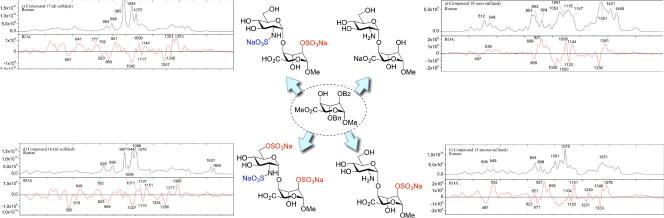

## 1 Introduction

Heparin and heparan sulfate (H/HS) are highly sulfated glycosaminoglycans (GAGs) involved in a variety of important biological recognition processes.^1^, ^2^, ^3^ The main repeating disaccharide unit of H/HS consists of an N-substituted d-glucosamine (GlcNX) unit α-1,4 linked to l-iduronic acid (l-IdoA) or d-glucuronic acid (d-GlcA). The rare l-IdoA sugar has some unique conformational properties, in being able to adopt readily interconverting chair (^1^*C*_4_ and ^4^*C*_1_) and skew-boat (^2^*S*_0_) conformers.^4^, ^5^, ^6^ This conformational plasticity has attracted considerable interest in seeking to understand how this additional flexibility of the idopyranose ring relates to the central role of H/HS in binding to, and regulating the biology of, a wide diversity of proteins. The conformational preferences differ as a function of protein binding in binary or ternary complexes^7^, ^8^, ^9^, ^10^, ^11^ although evidence suggests limited effects on the overall 3D shape of longer oligosaccharides.^12^ The role of sulfation state and the local environment (i.e., adjacent sugars in saccharides) is also evidently influential on the conformational equilibria, and thus has been of interest from theoretical^13^, ^14^, ^15^, ^16^, ^17^ and spectroscopic perspectives.^18^ Alongside backbone linkage and monomer unit differences, variations of sulfation are also key effectors in the biological roles of such oligosaccharides.^19^ There is growing evidence that changes in sulfation sites can also have a significant impact on binding selectivity and/or biology, even in shorter synthetic HS sequences or other GAGs.^19^, ^20^, ^21^, ^22^, ^23^

Although high resolution techniques such as crystallography and NMR spectroscopy have been used to investigate carbohydrate conformation, they are often difficult to apply to complex saccharides, and developing new methods to identify and characterize structural features and conformational dynamics in glycosylaminoglycans, such as the H/HS structures, are hence of great importance. Raman spectroscopy, which measures molecular vibrations, and Raman optical activity (ROA), which measures the small difference in Raman scattering from chiral molecules using circularly polarized light, are two complementary techniques that can provide such information. The combination of Raman and ROA can provide highly detailed information about the structure and conformational dynamics of biological molecules.^24^, ^25^, ^26^ Early ROA studies on mono- and disaccharides have demonstrated the sensitivity of ROA spectra to the identity of the sugar and the nature of the glycosidic linkage.^27^, ^28^, ^29^ Recently, we have used ROA to study the details of higher order structure in the non-sulfated glycosaminoglycan hyaluronan,^30^ glycan conformation in a high-mannose glycoprotein,^31^ and of the glycan of RNAseB^32^ and the effect of hydration on methy-l-glucose dynamics.^33^ Although Raman has been employed to begin to investigate the effects of sulfation on carbohydrate structure,^34^, ^35^ and ROA spectra have previously been reported for H/HS,^36^ much work remains to be undertaken to exploit the potential of ROA spectroscopy as a probe of structural effects of sulfation upon carbohydrates.

The highly heterogeneous nature of native H/HS, both in terms of backbone and sulfation patterns, means that access to structurally well-defined, simplified systems are essential in order to develop the structural accuracy of such analytical methods, that is, linking the specific and differentiated structural features of the repeating unit to the data obtained from Raman/ROA. Of particular interest in this context is characterizing the relationship of Raman/ROA spectra to variations in GAG sulfation patterns and the influence this has on conformational dynamics. To this end we report here the synthesis of a [GlcN–IdoA] library of methyl glycoside-capped disaccharides containing defined sulfation patterns across the IdoA O2, GlcN O6 and 2-amino sites (native sulfation sites), as a programmed matrix of sulfation variations for analysis using Raman/ROA spectroscopy. Whilst the disaccharide is the minimal unit to evaluate applications of Raman/ROA on sulfation variations, the biological relevance of such disaccharides has also been evidenced by work involving protein-binding experiments.^23^

Whilst NMR data confirm that the constituent monosaccharides in all sulfated disaccharides adopt consistent ring conformations, Raman/ROA identifies spectral features indicative of different effects on global conformation, which can be related to the variation in sites of sulfation. Comparison here with digest heparin indicates that the disaccharide unit could prove a suitable model for longer, sulfated heparin-related GAGs.

## 2 Heparin-like disaccharides

A diversity of sulfated disaccharides of the [IdoA–GlcN] type obtained from enzymatic depolymerisation methods has been previously characterized by NMR,^37^ and a number of synthetic routes to these types of HS disaccharide have been described.^38^ However, the reverse sequence disaccharide unit [GlcN–IdoA] is also of interest^23^, ^39^, ^40^ and it is the repeat unit in a number of longer synthetic heparanoid mimetics recently reported.^19^, ^41^, ^42^ This led us to target the synthesis of a sulfation-varied matrix of this repeat unit through our readily accessible l-idopyranoside **1**,^19^ chosen as the preferred acceptor for disaccharide incorporation, as it has the α-configuration found in native heparin linkages.

**Scheme 1:**
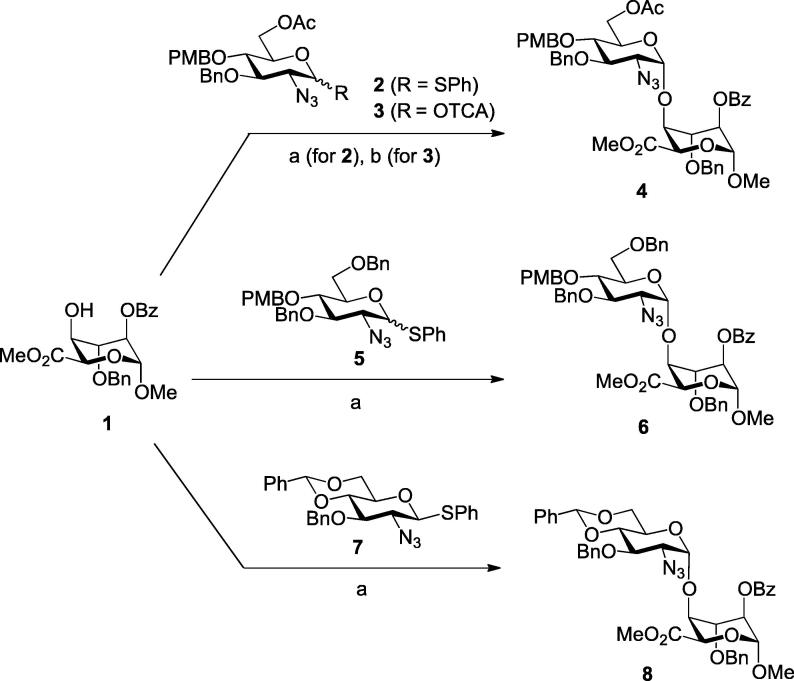
Reagents and conditions: (a) NIS, AgOTf, DCM, 92% for **4** via **2**, 77% for **6** (6:1 α/β), 80% for **8** (12:1, α/β) (b) TMSOTf, DCM, 95% for **4** via **3**.

Differentially-protected thioglycoside and trichloroacetimidate d-GlcN derivatives,^43^ having 2-azido non-participating groups were thus chosen as complementary donors for the synthesis of heparin-like disaccharides **4**, **6** and **8** (Scheme 1). This set of disaccharide targets was selected to enable divergence to a matrix of the biologically-related, site-specific sulfation patterns incorporating l-IdoA-O2S, d-GlcN-O6S and d-GlcNS, which are the key sites of sulfation variation in native H/HS oligosaccharides (with obligatory l-IdoA O2 sulfation). Moreover, variations in sulfation at these sites (particularly N-2 and O-6 of d-GlcN) are strongly implicated in many biological effects. An elegant approach recently reported by Hung et al.^23^ also provides disaccharides of this type, exploiting an l-iduronate-lactone based access (from a late stage l-iditol oxidation). Here, we exploit pre-installation of the iduronate carboxylate at the outset, which provides a complementary access to a matrix of IdoA–GlcN disaccharides with different combinations of sulfation at the common native sulfation sites.

All glycosylations of **1** proceeded in good yields and high anomeric selectivities for formation of the desired α-linked disaccharides and were utilized to access gram amounts of these key intermediates, for example **4** was prepared on 1.8 g scale from **2** (Scheme 1). Disaccharide **4** was formed with no detectable amounts of the β anomer with utilization of either trichloroacetimidate (**3**) or thiophenyl (**2**) as the anomeric leaving group. The d-GlcN-O6 benzyl-protected donor **5** yielded **6** as a 6:1 α/β mixture (from ^1^H NMR) of diastereoisomers which were readily separable by chromatography to afford the pure α anomer, again on gram-scale.

Anomeric selectivity and yield were also high for coupling 4,6-benzylidene protected donor **7** with acceptor **1** to afford the benzylidene-protected disaccharide **8**. The utilization of a benzylidene-protected d-GlcN unit was selected not only to access 4,6-unsulfated compounds of type **18** (vide infra), but also to demonstrate the utility of our thioglycoside donor/iduronate acceptor system. This complements earlier systems developed using trichloroacetimidate derived d-GlcN donors,^44^ providing an alternative means to prepare disaccharide-level benzylidene systems, suitable for late stage 4,6-positional manipulations.

**Scheme 2:**
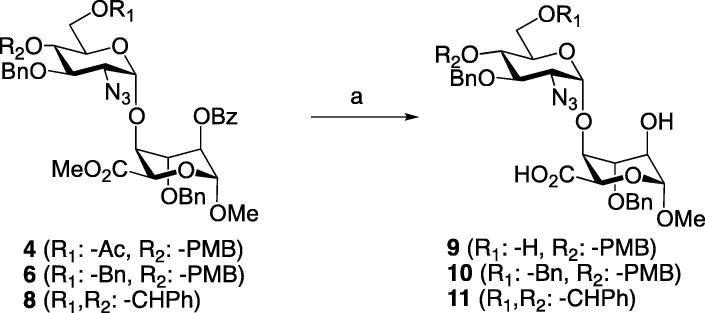
Reagents and conditions: (a) LiOH, THF/MeOH/H_2_O, 58% for **9**, 75% for **10**, 90% for **11**.

Disaccharides **4**, **6** and **8** were next taken through deprotection and sulfation sequences to afford the final sulfation-varied library. Firstly, treatment of all three disaccharides with LiOH in THF/MeOH/H_2_O provided the disaccharide carboxylic acids **9**–**11** (Scheme 2). This step was necessary to expose the l-IdoA-O2 position for sulfation on all species, whilst retaining options for selectivity at the GlcN-O6 position.

**Scheme 3:**
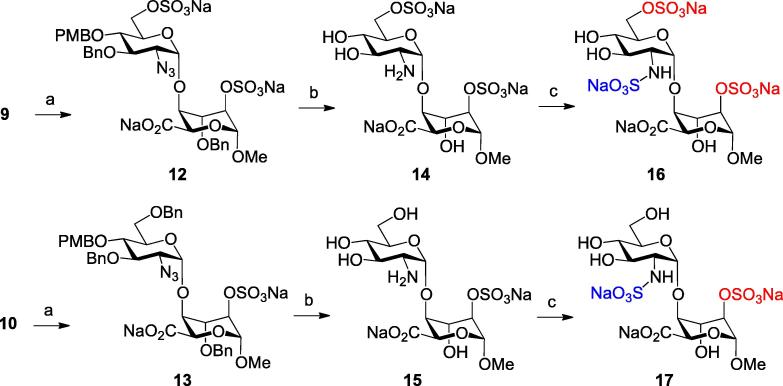
Reagents and conditions: (a) Py·SO_3_ complex, pyridine, 79% for **12**, 72% for **13** (b) H_2_, Pd(OH)_2_/C, MeOH/H_2_O, 99% for **14**, 99% for **15** (c) Py·SO_3_ complex, H_2_O, 79% for **16**, 92% for **17**.

Thus, 2,6-di-O-sulfation of **9** and individual 2-O-sulfation of **10**, afforded intermediates **12** and **13** respectively, in good yields. Subsequent hydrogenolysis then removed all benzylic ether linkages with concomitant azide reduction to afford heparin-like disaccharides **14** and **15**, differing only in their d-GlcN-O6 sulfation. Finally, N-sulfation was completed using SO_3_·pyridine complex in water to afford the d-GlcNS6S and d-GlcNS disaccharides **16** and **17** respectively (Scheme 3). This afforded four alternative variants of the final sulfation pattern for this disaccharide scaffold (**14**–**17**).

**Scheme 4:**
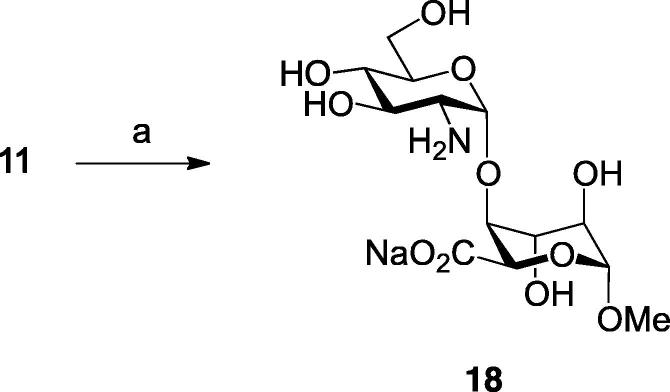
Reagents and conditions: (a) H_2_, Pd(OH)_2_/C, H_2_O, MeOH, 95%.

Alongside this we also wanted to diverge to the corresponding disaccharide lacking any sulfation. This would complete a matrix from completely non-sulfated to fully trisulfated variants, encompassing the main patterns seen in native heparin-like species. To access this unsulfated disaccharide, benzylidene-protected disaccharide **11** was exhaustively hydrogenolized to give the fully deprotected and unsulfated d-GlcN–l-IdoA species **18** (Scheme 4).

The synthetic approach detailed here thus allows a convenient access to a matrix of the key, biologically-relevant and differentially sulfated H/HS disaccharides. These are a useful tool-kit to investigate structural analysis of these minimal disaccharide repeat units of the native ligands.

**Figure 1:**
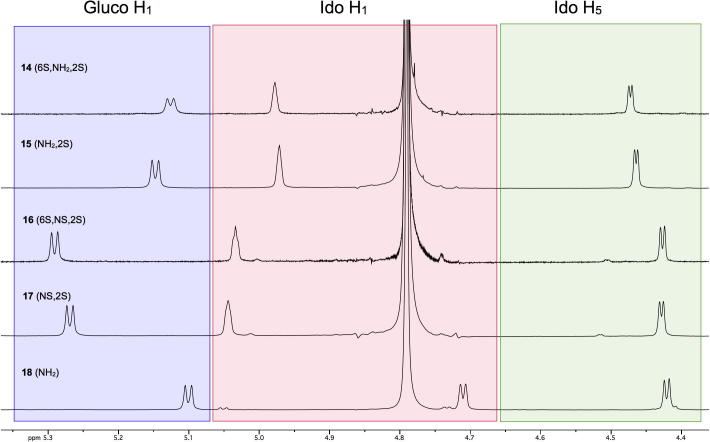
Key anomeric region ^1^H NMR data for **14**–**18** (Spectra recorded at 400 MHz in D_2_O).

The ^1^H NMR spectra of **14**–**18** were consistent with the anticipated effects of adding sulfation at O-2 of l-IdoA and NH_2_ of GlcN on their respective anomeric protons (in all cases approximately 0.2–0.3 ppm shift downfield). These data are overlaid in Figure 1. ^1^H NMR data also showed the presence of a rare *J*_2_,_4_ ‘*W*’ coupling in compound **15**, indicating l-iduronate components of the disaccharide adopting a ^1^*C*_4_ conformation (see Section 5).

Whilst these ^1^H NMR data can confirm shift effects, they do not readily provide indicative data about conformational change more broadly. We thus selected disaccharides **15**–**18**, which contain an increasing sulfation content (zero to three sulfates overall), for further analysis by Raman/ROA spectroscopy.

## 3 Raman/ROA analysis of HS disaccharides

**Figure 2:**
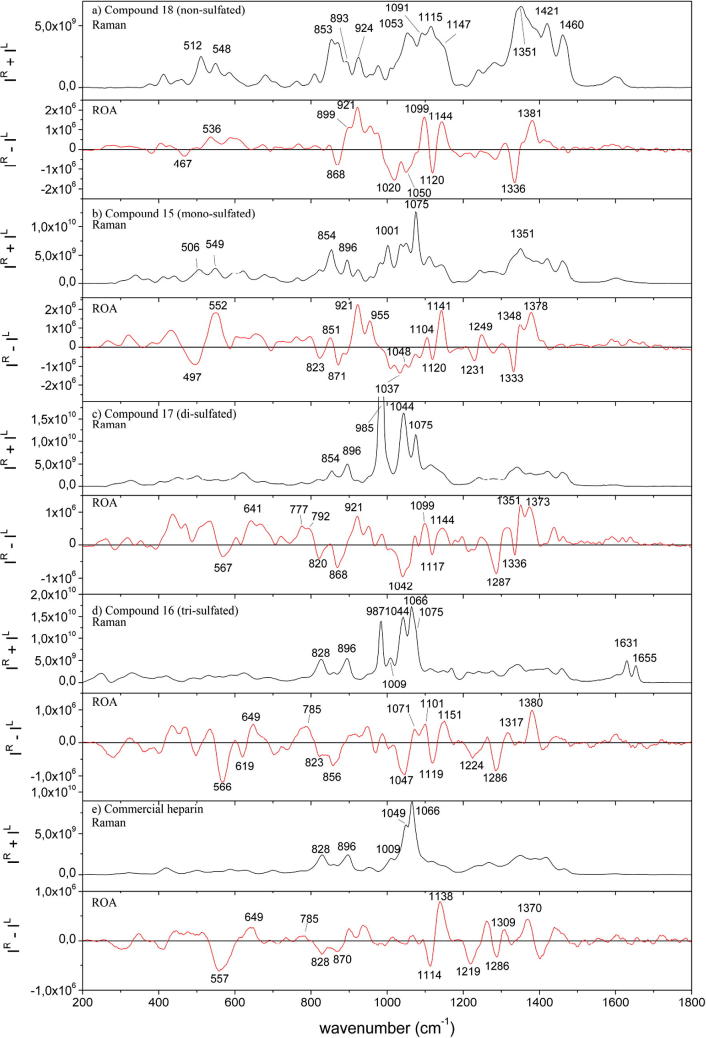
Raman (*I*^R^ + *I*^L^) and ROA (*I*^R^ − *I*^L^) spectra for compounds **15**–**18**, together with a set of spectra of commercially available heparin.

In Figure 2 we present the Raman (top, *I*^R^ + *I*^L^) and ROA (bottom, *I*^R^ − *I*^L^) spectra of (a) **18**; (b) **15**; (c) **17** and (d) **16**, together with (e) a commercial sample of heparin. Thus, we can directly compare the Raman and ROA spectra of the nonsulfated, sulfated, disulfated and trisulfated native forms of the GlcN–IdoA disaccharide. Differences are observable between these spectra, showing that Raman and ROA spectra are sensitive to the effects of sulfation on disaccharide conformation, which are discussed below.

The region between 900–1100 cm^−1^ in the Raman spectra presents the most obvious differences due to sulfation. A number of small-to-medium sized Raman features are found for the non-sulfated disaccharide (**18**), with a number of more intense and sharp bands then appearing for the different sulfated sugars. This is generally in good agreement with other reported Raman spectra of sulfated glycosaminoglycans.^45^, ^46^ In particular, a reasonably consistent set of Raman bands assigned to O—S—O_3_^−^ vibrations can be seen at ∼1075 cm^−1^ for the mono-, di- and tri-sulfated disaccharides (as a weak shoulder in the latter), suggesting that this band originates from the 2-O sulfation site. A small, yet reliable, band ∼896 cm^−1^ also appears to show a singular sensitivity to 2-O sulfation. The mono-sulfated compound **15** also generates a doublet of peaks at ∼1037 cm^−1^ and 1048 cm^−1^, which appear to merge into a stronger singlet at ∼1044 cm^−1^ upon further sulfation. This observation is in agreement with the reported association of this band with S=O symmetric stretching vibrations in N-sulfates,^47^ as only the di- and tri-sulfated species (**17** and **16**) contain N-sulfation. A similar sharp increase occurs for the band ∼987 cm^−1^, which is weak when no sulfate group is present, of moderate intensity upon 2-O-sulfation and much more intense upon addition of the other sulfate groups. This band appears to correspond to those of C—O—S vibrations previously reported,^46^ but is of far greater intensity here for the di- and tri-sulfated disaccharides. In addition, there is a band nearby at ∼1001 cm^−1^ for **15** that either completely or mostly disappears in the spectra of the other three samples. The most obvious difference for the spectrum of the tri-sulfated species 16 compared to the others is the sharp doublet appearing at 1631 and 1655 cm^−1^. Furthermore, a band appears at ∼1066 cm^−1^ in the tri-sulfated disaccharide, partly overlapping with the 1075 cm^−1^ band, and additionally, smaller bands at ∼828 cm^−1^ and ∼1009 cm^−1^. These three bands can hence be assigned as suggestive markers for 6-O-sulfation.

The corresponding ROA spectra display a complex set of changes in response to sulfation patterns. Overall these ROA spectra display profiles similar to those reported by Rudd et al.^36^ for a heparin sample, particularly the strong +/−/+ triplet observed from 1100–1150 and and the −/+ couplet couplet from 1300–1400 cm^−1^. Similar features were also reported by Zhu et al.^48^ for *N*,*N*′-diacetylchitobiose. Increasing sulfation then gives rise to a series of band changes. The +ve ROA bands at ∼1141, 1249 and 1378 cm^−1^ all become more intense upon sulfation at the O2 site (**15**) but are then reduced upon N-sulfation (**17**). Within the complex band structure 800–1000 cm^−1^ the 2-O-sulfated and non-sulfated disaccharides display similar profiles, though the non-sulfated disaccharide tends to display broader ROA bands and two additional features at 899 and 955 cm^−1^. One ROA marker band for 2-O-sulfation does appear as a negative band at ∼823 cm^−1^. Further sulfation at N_2_ and O6 (**16**) leads to substantial decreases in the intensity of many of these ROA bands, while the pattern remains intact. Interestingly, we note that many of these ROA bands for the tri-sulfated form are very similar to those of the di-sulfated form. These spectral variations all suggest that sulfation at the O2 site leads to relatively few changes in the conformation of the d-GlcN–α-1,4-l-IdoA-OMe disaccharide, but does reduce to some degree the conformational mobility of the carbohydrate. Spectral changes suggest subsequent sulfation at the GlcN–NH_2_ site does lead to significant conformational changes and also an increase in conformational freedom of the disaccharide.

The most significant spectral variations occur in the region of 500–600 cm^−1^. At slightly lower wavenumber, from ∼400–500 cm^−1^, in the ROA spectra for each of these four disaccharides we observe small couplets that are +ve at lower wavenumber and −ve at higher wavenumber, that are typical of the α-1–4 linkage type.^49^ Although these couplets do show some variability in position and shape they all clearly verify the existence of the α-1–4 linkage, as other linkage types show sign inversion of these spectral features. This consistent profile identifying the linkage between the two sugar rings is in stark contrast to the intense −ve/+ve ROA couplet at ∼497/549 cm^−1^ measured for the mono-sulfated disaccharide and the strong −ve band ∼566 cm^−1^ for the di- and tri-sulfated forms. Such features have not been reported previously in the ROA spectra of carbohydrates and no assignment is yet available for these bands. However, it does appear that this band monitors significant differences in a structural moiety. Although the identity of this structural feature is not yet known, it is possible that this band originates from C—O—S stretching coordinates mixing with C—N—S stretching vibrations.

In order to further study the sensitivity of ROA towards sulfation patterns in related native GAGs, we measured the Raman and ROA spectra of a commercial sample of heparin, also presented in Figure 2. The Raman spectrum of heparin is again (and as previously reported in literature) dominated by intense bands arising from the sulfate moieties. The Raman bands at ∼896 cm^−1^ and 1049 cm^−1^ (slightly shifted from ∼1044 cm^−1^) indicate 2-O- and N-sulfation, respectively, while the bands at ∼828 cm^−1^, ∼1009 cm^−1^ and 1066 cm^−1^ are clear indicators of 6-O-sulfation. Interestingly, no Raman bands are observed at ∼987 cm^−1^ or 1075 cm^−1^ in the heparin spectrum. As the marker band for 2-O-sulfation at ∼896 cm^−1^ is present in the heparin spectrum, the lack of the ∼1075 cm^−1^ band clearly indicates that this band is more prone to conformational shifting and must be overlapping with the ∼1066 cm^−1^ band in heparin. Therefore, the ∼896 cm^−1^ is a more reliable marker for 2-O-sulfation.

The ROA spectrum of heparin reveals some intriguing similarities, and differences, when compared to the spectra of the disaccharides. While the ROA spectrum of heparin is quite similar to those of the di- and tri-sulfated disaccharides in the low wavenumber region (up to 900 cm^−1^), implying similar conformational distributions and sulfation patterns, the highly conserved region in the ROA spectra of the disaccharides, between 1000 and 1200 cm^−1^, changes fundamentally in the spectrum of heparin. Johannessen et al.^31^ reported a corresponding similarity between the low frequency ROA signatures for the glycan component of invertase and those of component mannose saccharides, and concluded that the conformations around the glycosidic linkages in the intact glycoprotein were likely to be similar to those adopted by the free sugars. Above 1000 cm^−1^ the complicated pattern of ROA bands is reduced to one couplet, −ve at ∼1114 cm^−1^ and +ve at ∼1138 cm^−1^. Above 1200 cm^−1^, the ROA spectrum of heparin is again similar to those of the N-sulfated disaccharides, and the tri-sulfated disaccharide in particular.

## 4 Conclusions

An efficient, short route to an array of differentially sulfated GlcN–IdoA disaccharides has been identified, demonstrating that these targets can be routinely accessed on good scale, directly from l-iduronate components. These are specifically directed to provide the sulfation variability at the key biologically-relevant l-idoA O-2 and d-GlcN O-6 and amino sites. This has allowed the first evaluation of using Raman/ROA spectroscopy to characterize changes in spectra as a function of both site and level of sulfation with pure, defined species. This has provided new data to correlate to sulfation level and also enabled categorization of both similarities and differences to native digest heparin. Data suggest evidence of changes in conformations and conformational freedom as a function of specific sulfation-site changes at the disaccharide level. It is anticipated that this data set will open the way for applications to further site-specific sulfated saccharides.

## 5 Experimental section

### 5.1 Raman and Raman optical activity measurements

All Raman and ROA experiments were performed using a ChiralRaman spectrometer (BioTools Inc, Jupiter FL, USA) operated via Critical Link LLC software. The instrument was set up in the backscattering geometry using a Millenium Pro Nd/VO_4_ laser (SpectraPhysics, UK) with an excitation wavelength of 532 nm, laser power at sample ∼400 mw, spectral resolution ∼7 cm^−1^ and total acquisition times of ∼24 h. The parent Raman spectra (*I*^R^ + *I*^L^) and the ROA difference spectra (*I*^R^ − *I*^L^) were recorded simultaneously. For each sample ∼100 μL of solution was filtered through a 0.22 μm spin filter (Merck Millipore, Germany) prior to being pipetted into a quartz cell (Starna, UK).

#### 5.1.1 Methyl 6-*O*-acetyl-2-azido-3-*O*-benzyl-2-deoxy-4-*O*-*p*-methoxybenzyl-α-d-glucopyranosyl-(1→4)-(methyl 2-*O*-benzoyl-3-*O*-benzyl-α-l-idopyranoside)uronate **4**

##### 5.1.1.1 From thioglycoside donor **2**

Glucosamine derived donor **2** (1.65 g, 2.97 mmol) and acceptor **1** (950 mg, 2.28 mmol) were mixed together, evaporated from dry toluene (3 × 20 mL) and left under high vacuum for 1 h. The suspension was diluted under a nitrogen atmosphere with dry DCM (45 mL) and 4 Å molecular sieves (800 mg) added. The mixture was cooled to 0 °C and NIS (2.5 g, 11.4 mmol) added. Then the mixture was stirred for a further 15 min at the same temperature whereupon a catalytic amount of AgOTf was added. The mixture was kept under a nitrogen atmosphere at 0 °C for a further 30 min. The suspension was then quenched by addition of a 1:1 mixture of saturated aqueous solution of Na_2_S_2_O_3_ and NaHCO_3_ (10 mL total) and filtered through Celite. The phases were separated and the organic layer washed with brine. The crude was then purified by column chromatography (EA/hexane 1:4→2:3), to yield **4** (1.8 g, 2.10 mmol, 92%) as a foam.

##### 5.1.1.2 From trichloroacetimidate donor **3**

Both reaction components, trichloroacetimidate **3** (430 mg, 1.04 mmol) and alcohol **1** (855 mg, 1.42 mmol) were combined, co-evaporated from dry toluene (3 × 10 mL) and dried under high vacuum for 1 h. To this mixture was added dry DCM (20 mL) and reaction was cooled to −30 °C. TMSOTf (2 μL, 0.01 mmol) was added and reaction monitored by TLC (EtOAc/hexane 1:3) until all alcohol was consumed (30 min). Then the reaction was quenched by addition of Et_3_N (0.2 mL) and solvents were evaporated in vacuo. The product **4** (850 mg, 0.99 mmol, 95%) was isolated after column chromatography (EtOAc/hexane 2:9). *R_f_ *= 0.5 (EtOAc/hexane 1:3); [*α*]_D_^20^ = −13.0 (*c* 10.0, CH_2_Cl_2_); ^1^H NMR (400 MHz; CDCl_3_) *δ* 8.13 (dd, *J *= 8.8, 1.2 Hz, 2H, Ph), 7.48–7.13 (m, 15H, Ph), 6.90 (d, *J *= 8.8 Hz, 2H, Ph), 5.13 (br s, 1H, H-1), 5.10 (br s, 1H, H-2), 4.95 (d, *J *= 12.0 Hz, 1H, C*H*_2_Ph), 4.85 (d, *J *= 2.0 Hz, 1H, H-5), 4.78 (d, *J *= 12.0 Hz, 1H, C*H*_2_Ph), 4.67 (d, *J *= 3.2 Hz, 1H, H-1′), 4.65 (d, *J *= 12.0 Hz, 1H, C*H*_2_Ph), 4.47 (d, *J *= 10.8 Hz, 1H, C*H*_2_Ph), 4.38 (dd, *J *= 12.0, 2.4 Hz, 1H, H-6′A), 4.28 (dd, *J *= 12.0, 3.2 Hz, 1H, H-6′B), 4.15–4.09 (m, 2H, C*H*_2_Ph, H-3), 4.03–3.97 (m, 3H, H-4′, H-4, H-5′), 3.95 (d, *J *= 10.8 Hz, 1H, C*H*_2_Ph), 3.85 (s, 3H, OC*H*_3_), 3.82 (s, 3H, OC*H*_3_), 3.52 (s, 3H, C(O)OC*H*_3_), 3.46 (t, *J *= 10.0 Hz, 1H, H-3′), 3.18 (dd, *J *= 10.0, 3.2 Hz, 1H, H-2′), 2.04 (s, 3H, C(O)CH_3_); ^13^C NMR (100 MHz; CDCl_3_) *δ* 170.6, 169.6, 165.5, 159.4, 137.7, 137.4, 133.3, 130.0, 129.7, 129.6, 128.8, 128.5, 128.4, 128.2, 128.1, 127.8, 113.8, 100.4, 99.7, 80.2, 76.1, 74.67, 74.51, 72.7, 72.4, 70.2, 67.8, 66.9, 63.8, 62.4, 56.3, 55.3, 52.5, 20.9; HRMS (FT-MS NSI^+^) *m*/*z* calcd for C_45_H_53_N_4_O_14_ [M+NH_4_]^+^ 873.3555, found 873.3553.

#### 5.1.2 Methyl 2-azido-3,6-di-*O*-benzyl-2-deoxy-4-*O*-*p*-methoxybenzyl-α-d-glucopyranosyl-(1→4)-(methyl 2-*O*-benzoyl-3-*O*-benzyl-α-l-idopyranoside) uronate **6**

Acceptor monosaccharide **1** (0.83 g, 2.04 mmol) and thioglycoside donor **5** (1.46 g, 2.45 mmol) were dissolved in dry DCM (15 mL) under N_2_. Freshly activated 4 Å powdered molecular sieves (0.59 g) were added and the solution cooled to −15 °C. After 10 min, NIS (0.92 g, 4.09 mmol) was added and after another 10 min AgOTf (10 mg, 0.041 mmol) added. The suspension changed colour from pale yellow to deep red, was stirred for 45 min and more AgOTf added (10 mg, 0.041 mmol). The reaction was quenched after 2½ h into a separating funnel containing a mixture of DCM (100 mL), saturated aqueous NaHCO_3_ (80 mL) and Na_2_S_2_O_3_ (20 mL, 10% aqueous). After shaking until the iodine colour was removed, the suspension was filtered through a short pad of Celite® washing with water and DCM. The layers were separated and the aqueous extracted with DCM (20 mL). The organic layers were combined, dried (MgSO_4_) and solvent removed in vacuo. The material was purified by silica gel flash column chromatography (EtOAc/hexane 1:2) to give **6** (1.40 g, 1.55 mmol, 77%) as a white foam. *R_f_ *= 0.32 (EtOAc/hexane 1:2); [*α*]_D_^20^ = −48.0 (*c* 1.0, CH_2_Cl_2_); ^1^H NMR (400 MHz; CDCl_3_) *δ* 8.01 (dd, *J* = 8.4, 1.2 Hz, 2H, Ph), 7.35–7.14 (m, 16H, Ph), 7.04 (dd, *J *= 7.7, 1.6 Hz, 2H, Ph), 6.98 (d, *J *= 8.7 Hz, 2H, Ph), 6.77 (d, *J *= 8.7 Hz, 2H, Ph), 5.04 (s, 1H, H-1), 4.98 (s, 1H, H-2), 4.84 (d, *J *= 11.9 Hz, 1H, C*H*_2_Ph), 4.75 (d, *J *= 2.0 Hz, 1H, H-5), 4.68 (d, *J *= 11.9 Hz, 1H, C*H*_2_Ph), 4.62 (d, *J *= 3.5 Hz, 1H, H-1′), 4.51 (t, *J *= 10.6 Hz, 2H, C*H*_2_Ph), 4.39 (d, *J *= 12.0 Hz, 1H, C*H*_2_Ph), 4.33 (d, *J *= 10.5 Hz, 1H, C*H*_2_Ph), 4.09 (d, *J *= 10.8 Hz, 1H, C*H*_2_Ph), 4.04 (br s, 1H, H-3), 3.94 (t, *J *= 2.4 Hz, 1H, H-4), 3.84–3.79 (m, 2H, C*H*_2_Ph, H-6′A), 3.74 (s, 3H, OC*H*_3_), 3.73–3.71 (m, 1H, H-6′B), 3.66 (s, 3H, OC*H*_3_), 3.61–3.55 (m, 2H, H-4′, H-5′), 3.44–3.40 (m, 4H, H-3′, C(O)OC*H*_3_), 3.12 (dd, *J *= 10.2, 3.5 Hz, 1H, H-2′); ^13^C NMR (100 MHz; CDCl_3_) *δ*.169.6, 165.5, 159.2, 137.9, 137.8, 137.4, 133.2, 130.6, 130.0, 129.6, 129.4, 128.8, 128.5, 128.4, 128.3, 128.3, 128.2, 128.0, 127.8, 127.7, 127.66, 113.6, 100.4, 100.0, 80.0, 77.4, 76.0, 74.6, 74.4, 73.6, 73.0, 72.4, 71.7, 67.8, 67.1, 63.7, 56.2, 55.3, 52.3; HRMS (ESI^+^) *m*/*z* calcd for C_50_H_53_N_3_O_13_Na [M+Na]^+^ 926.3471, found 926.3471.

#### 5.1.3 Methyl 2-azido-3-*O*-benzyl-4,6-*O*-benzylidene-2-deoxy-α-d-glucopyranosyl-(1→4)-(methyl 2-*O*-benzoyl-3-*O*-benzyl-α-l-idopyranoside) uronate **8**

Acceptor monosaccharide **1** (234 mg, 0.56 mmol) and thioglycoside donor **7** (355 mg, 0.75 mmol) were dissolved in dry DCM (4 mL) under N_2_. Freshly activated 4 Å powdered molecular sieves (0.22 g) were added and the solution cooled to 0 °C in an ice-bath. After 10 min NIS (259 mg, 1.15 mmol) was added and after another 10 min AgOTf (7 mg, 0.03 mmol) was added. The suspension changed colour from pale yellow to deep red, was stirred for 45 min and more AgOTf added (8 mg, 0.03 mmol). The reaction was quenched after 90 min into a separating funnel containing a mixture of DCM (30 mL), saturated aqueous NaHCO_3_ (30 mL) and Na_2_S_2_O_3_ (5 mL, 10% aqueous). After shaking until the iodine colour was removed, the suspension was filtered through a short pad of Celite® washing with water and DCM. The layers were separated and the aqueous extracted with DCM (10 mL). The organic layers were combined, dried (MgSO_4_) and solvent removed in vacuo. The crude was purified by silica gel flash column chromatography (EtOAc/hexane 1:3) to give **8 (**353 mg, 80%, 0.45 mmol, α/β 12:1) as a white foam. *R_f_* 0.21 (EtOAc/Hexane 1:3); [*α*]_D_^20^ = +66.5 (*c* 0.7, CH_2_Cl_2_) Data for major α anomer: ^1^H NMR (400 MHz; CDCl_3_) *δ* 8.18–8.15 (m, 2H, Bz), 7.53–7.13 (m, 18H, Ph), 5.53 (s, 1H, PhC*H*O), 5.16–5.15 (m, 1H, H-2), 5.14–5.13 (m, 1H, H-1), 4.96 (d, *J *= 12 Hz, 1H, C*H*_2_Ph), 4.88 (d, *J *= 2.0 Hz, 1H, H-5), 4.79 (d, *J *= 12 Hz, 1H, C*H*_2_Ph), 4.65 (d, *J *= 3.6 Hz, 1H, H-1′), 4.44–4.41 (m, 1H, C*H*_2_Ph), 4.38 (dd, *J *= 10.0, 4.8 Hz, 1H, H-4′), 4.16–4.14 (m, 1H, H-3), 4.06–4.05 (m, 1H, H-4), 4.02 (dt, *J *= 9.6, 4.8 Hz, 1H, H-5′), 3.96–3.93 (m, 1H, C*H*_2_Ph), 3.81 (s, 3H, C(O)OC*H*_3_), 3.68–3.54 (m, 3H, H-3′, H-6′A, H-6′B), 3.53 (s, 3H, OC*H*_3_), 3.21 (dd, *J *= 9.6, 3.6 Hz, 1H, H-2′); ^13^C NMR (100 MHz; CDCl_3_) *δ* 169.6, 165.6, 137.9, 137.6, 137.5, 133.3, 130.0, 129.7, 129.1, 128.8, 128.5, 128.4, 128.1, 128.0, 127.8, 126.2, 101.5, 100.5, 99.9, 82.4, 76.7, 75.9, 74.8, 72.8, 72.5, 68.6, 67.8, 67.0, 63.5, 63.2, 56.3, 52.4; HRMS (ESI^+^) *m*/*z*: calcd for C_42_H_43_NaN_3_O_12_ [M+Na]^+^: 804.2739, found: 804.2720.

#### 5.1.4 2-Azido-3-*O*-benzyl-2-deoxy-4-*O*-*p*-methoxybenzyl-α-d-glucopyranosyl-(1→4)-(methyl 3-*O*-benzyl-α-l-idopyranoside)uronic acid **9**

Disaccharide **4** (400 mg, 0.47 mmol) was dissolved in THF (3.0 mL) and MeOH (1 mL). The solution was cooled to 0 °C and LiOH (105 mg, 1.88 mmol) in H_2_O (1 mL) was added dropwise. The solution was stirred for 3 h at this temperature and quenched with 1 M HCl (50 μL). Solvents were removed in vacuo and the residue partitioned between EtOAc (50 mL) and water (10 mL). The layers were separated, the organics dried (MgSO_4_) and solvent removed in vacuo to give the crude material as a clear oil. This was purified by silica gel flash chromatography, eluting with DCM/MeOH, 95:5 to give **9** (190 mg, 0.27 mmol, 58%) as a white foam. *R_f_* 0.41 (DCM/MeOH 9:1); [*α*]_D_^20^ = −40.3 (*c* 4.0, MeOH); ^1^H NMR (400 MHz; MeOD) *δ* 7.43–7.30 (m, 10H, Ph), 7.22 (d, *J *= 8.7 Hz, 2H, Ph), 6.86 (d, *J *= 8.8 Hz, 2H, Ph), 5.13 (d, *J *= 3.8 Hz, 1H, H-1′), 4.89 (d, *J *= 11.0 Hz, 1H, C*H*_2_Ph), 4.87 (d, *J *= 2.8 Hz, 1H, H-1), 4.83–4.79 (m, 2H, C*H*_2_Ph), 4.73–4.68 (m, 3H, H-5, 2× C*H*_2_Ph), 4.63 (d, *J *= 10.5 Hz, 1H, C*H*_2_Ph), 4.18 (t, *J *= 3.3 Hz, 1H, H-4), 3.92 (t, *J *= 4.1 Hz, 1H, H-3), 3.89–3.87 (m, 1H, H-3′), 3.79 (s, 3H, PMBOC*H*_3_), 3.77–3.73 (m, 3H, H-5′, H-6′A, H-6′B), 3.68–3.67 (m, 1H, H-2), 3.63 (t, *J *= 9.2 Hz, 2H, H-4′), 3.57 (dd, *J *= 3.8, 10.3 Hz, 2H, H-2′), 3.45 (s, 3H, OC*H*_3_); ^13^C NMR (100 MHz; CD_3_OD) *δ* 172.9, 160.9, 139.5, 139.4, 131.7, 130.6, 129.5, 129.4, 129.0, 128.9, 128.7, 114.7, 104.0, 97.3, 81.9, 78.8, 76.4, 75.7, 75.5, 74.0, 73.8, 73.7, 69.5, 69.1, 65.2, 61.4, 56.5, 55.7; HRMS (FTMS NSI^−^) *m*/*z* calcd for C_35_H_40_N_3_O_12_ [M−H]^−^ 694.2617, found 694.2607.

#### 5.1.5 2-Azido-3,6-di-*O*-benzyl-2-deoxy-4-*O*-*p*-methoxybenzyl-α-d-glucopyranosyl-(1→4)-(methyl 3-*O*-benzyl-α-l-idopyranoside)uronic acid **10**

Disaccharide **6** (695 mg, 0.78 mmol) was dissolved in THF (5.0 mL) and MeOH (2 mL). The solution was cooled to 0 °C and KOH (92 mg, 1.63 mmol) in H_2_O (2 mL) was added dropwise. The solution was stirred for 3 h at this temperature. The reaction was extracted with EtOAc (2 × 50 mL) and HCl (25 mL, 0.1 M), dried (MgSO_4_), filtered and solvent removed in vacuo to give the crude material as a clear oil. This was purified by silica gel flash chromatography (EtOAc/Hexane 1:2 + 1% HCOOH) to give **10** (452 mg, 0.58 mmol, 75%) as a white foam. Further purification was achieved by crystallization (dissolved 0.35 g in 2 mL EtOAc and added 6 mL hexane) to yield fine white needles. *R_f_ *= 0.25 (EtOAc/hexane 1:1 + 1% HCOOH); mp 118–119 °C; [*α*]_D_^20^ = −18.8 (*c* 0.41, CH_2_Cl_2_); ^1^H NMR (400 MHz; CDCl_3_) *δ* 7.38–7.28 (m, 15H, Ph), 7.06 (d, *J *= 8.8 Hz, 2H, Ph), 6.80 (d, *J *= 8.8 Hz, 2H, Ph), 4.96–4.95 (m, 1H, H-1), 4.92 (d, *J *= 4.0 Hz, 1H, H-1′), 4.88–4.67 (m, 5H, H-5, 2× C*H*_2_Ph), 4.64 (d, 1H, *J *= 12 Hz, C*H*_2_Ph), 4.60 (d, 1H, *J *= 12 Hz, C*H*_2_Ph), 4.74–4.44 (m, 2H, d, 1H, *J *= 12 Hz, C*H*_2_Ph), 4.24–4.23 (m, 1H, H-3), 3.86–3.59 (m, 8H, H-2, H-4, H-2′, H-3′, H-4′, H-5′, H-6′A, H-6′B), 3.78 (s, 3H, PMBOC*H*_3_), 3.45 (s, 3H, OC*H*_3_); ^13^C NMR (100 MHz; CDCl_3_) *δ* 170.4, 159.3, 137.6, 137.4, 137.2, 129.9, 129.5, 128.6, 128.5, 128.1, 128.1, 128.0, 128.0, 127.8, 113.8, 103.2, 95.4, 81.1, 77.4, 75.7, 74.5, 73.6, 72.2, 71.9, 71.7, 71.3, 68.3, 66.3, 66.0, 63.7, 56.4, 55.3; HRMS (ESI^−^) *m*/*z* calcd for C_42_H_46_N_3_O_12_ [M−H]^−^ 784.3086, found 784.3099. Elemental analysis calcd for C_42_H_47_N_3_O_12_: C 64.19, H 6.03, N 5.35; found C 64.23, H 6.27, N 5.35.

#### 5.1.6 2-Azido-3-*O*-benzyl-4,6-*O*-benzylidene-2-deoxy-α-d-glucopyranosyl-(1→4)-(methyl 3-*O*-benzyl-β-l-idopyranoside)uronic acid **11**

Disaccharide **8** (310 mg, 0.40 mmol) was dissolved in THF (5.0 mL) and MeOH (2 mL). The solution was cooled to 0 °C and KOH (57 mg, 1.02 mmol) in H_2_O (2 mL) was added dropwise. The solution was stirred for 3 h at this temperature. The reaction was extracted with EtOAc (2 × 25 mL) and HCl (25 mL, 0.1 M) and then dried (MgSO_4_), filtered and solvent removed in vacuo to give the crude material as a clear oil. This was purified by silica gel flash chromatography (EtOAc/Hexane 1:2 + 1% HCOOH) to give **11** (235 mg, 0.38 mmol, 90%) as a white foam. *R_f_ *= 0.09 (EtOAc/hexane 1:1 + 1% HCOOH); [*α*]_D_^20^ = −71.5 (*c* 0.20, CH_2_Cl_2_); ^1^H NMR (400 MHz; CDCl_3_) *δ* 7.50–7.19 (m, 15H, Ph), 5.52 (s, 1H, PhC*H*O_2_), 4.95 (s, 1H, H-1), 4.93 (d, 1H, *J *= 10.8 Hz, C*H*_2_Ph), 4.85 (d, *J *= 1.6 Hz, 1H, H-5), 4.78 (d, *J *= 4.4 Hz, 1H, H-1′), 4.75 (d, 1H, *J *= 10.8 Hz, C*H*_2_Ph), 4.74 (d, 1H, *J *= 12 Hz, C*H*_2_Ph), 4.59 (d, 1H, *J *= 12 Hz, C*H*_2_Ph), 4.75–4.57 (m, 2H, C*H*_2_Ph), 4.30 (dd, *J *= 10.0, 4.8 Hz, 1H, H-4′), 4.19–4.18 (m, 1H, H-4), 3.97 (t, *J *= 10.0 Hz, 1H, H-3′), 3.88–3.87 (m, 1H, H-2), 3.82–3.81 (m, 1H, H-3), 3.76 (dt, *J *= 10.0, 4.8 Hz, 1H, H-5′), 3.65 (d, *J *= 11.6 Hz, 1H, H-6′A), 3.62 (d, *J *= 10.8 Hz, 1H, H-6′B), 3.58 (dd, *J *= 9.6, 4 Hz, H-2′), 3.38 (s, 3H, OC*H*_3_); ^13^C NMR (100 MHz; CDCl_3_) *δ* 171.2, 137.8, 137.3, 137.1, 129.3, 129.2, 128.7, 128.6, 128.5, 128.4, 128.3, 128.3, 128.1, 128.0, 126.2, 125.5, 103.2, 101.6, 95.7, 82.0, 78.0, 75.4, 72.2, 71.9, 71.4, 68.4, 66.2, 66.1, 63.5, 63.3, 56.4; HRMS (ESI^−^) *m*/*z* calcd for C_34_H_36_N_3_O_11_ [M−H]^−^ 662.2355, found 662.2357.

#### 5.1.7 2-Azido-3-*O*-benzyl-2-deoxy-4-*O*-*p*-methoxybenzyl-6-*O*-sulfonato-α-d-glucopyranosyl-(1→4)-(methyl 3-*O*-benzyl-2-*O*-sulfonato-α-l-idopyranoside)uronic acid di sodium salt **12**

Disaccharide **9** (190 mg, 0.27 mmol) was dissolved in dry pyridine (2.0 mL) under N_2_ at rt. SO_3_·Py (258 mg, 1.62 mmol, 6.0 equiv) was added and the suspension stirred at rt overnight. The reaction was quenched by addition of NaHCO_3_ (350 mg) in H_2_O (1 mL) and the solvents removed in vacuo. The crude white paste was then purified by silica gel flash chromatography, eluting with DCM/MeOH, 9:1, 4:1 and the converted to the sodium form using with Amberlite IRC 86 Na^+^ resin to give **12** (196 mg, 0.21 mmol, 79%) as a white glass. *R_f_* = 0.25 (5:1, DCM/MeOH + 1% NH_4_OH); [*α*]_D_^20^ = −13.8 (*c* 0.6, MeOH); ^1^H NMR (400 MHR, MeOD) *δ* 7.32–7.14 (m, 12H, Ph), 6.70 (d, *J *= 8.7 Hz, 2H, PMB), 5.00 (d, *J *= 3.9 Hz, 1H, H-1′), 4.90 (s, 1H, H-1), 4.85–4.81 (m, 2H, C*H*_2_Ph), 4.67 (d, 1H, *J *= 10 Hz, C*H*_2_Ph), 4.65 (d, 1H, *J *= 12 Hz, C*H*_2_Ph), 4.56 (d, 1H, *J *= 12 Hz, C*H*_2_Ph), 4.54 (d, 1H, *J *= 10 Hz, C*H*_2_Ph), 4.45 (d, *J *= 1.6 Hz, 1H, H-5), 4.32–4.27 (m, 2H, H-2, H-6′A), 4.15 (dd, *J *= 10.9, 1.8 Hz, 1H, H-6′B), 4.10 (s, 1H, H-3), 4.04 (s, 1H, H-4), 3.86 (dm, 1H, *J *= 12 Hz, H5′), 3.79 (t, *J *= 9.6 Hz, 1H, H-3′), 3.66 (s, 3H, PMBOMe), 3.55 (t, *J *= 9.6 Hz, 1H, H-4′), 3.42 (dd, *J *= 10.2, 3.8 Hz, 1H, H-2′), 3.31 (s, 3H, OMe); ^13^C NMR (100 MHz; MeOD) *δ* 175.8, 160.8, 140.0, 139.2, 131.9, 131.2, 129.4, 129.3, 129.0, 128.8, 128.7, 128.6, 114.6, 101.6, 95.4, 82.2, 78.8, 76.3, 75.7, 73.0, 71.6, 71.4, 71.4, 71.3, 67.1, 65.6, 56.0, 55.7; HRMS (FT MS NSI^−^) *m*/*z* calcd for C_35_H_39_N_3_ NaO_18_S_2_ [M−Na]^−^ 876.1573, found 876.1536.

#### 5.1.8 2-Azido-3,6-di-*O*-benzyl-2-deoxy-4-*O*-*p*-methoxybenzyl-α-d-glucopyranosyl-(1→4)-(methyl 2-*O*-sulfonato-3-*O*-benzyl-α-l-idopyranoside)uronic acid mono sodium salt **13**

Disaccharide **10** (193 mg, 0.25 mmol) was dissolved in dry pyridine (4.0 mL) under N_2_ at rt. Pyridine sulfur trioxide complex (119 mg, 0.78 mmol) was added and the suspension stirred at rt for 3 h. The reaction was quenched by addition of MeOH (0.3 mL) and the solvents removed in vacuo. The crude white paste was then purified by silica gel flash chromatography (DCM/MeOH, 15:1, 10:1 to 5:1). The product was then converted to the disodium salt by stirring with Amberlite IRC 86 Na^+^ resin (0.5 g) in MeOH (6 mL) for 2 h, filtered and evaporated to yield **13** (161 mg, 0.18 mmol, 72%) as a white glass. *R_f_ *= 0.27 (DCM/MeOH 5:1); [*α*]_D_^20^ = +2.9 (*c* 0.26, MeOH); ^1^H NMR (400 MHz; CD_3_OD) *δ* 7.40–7.23 (m, 15H, Ph), 7.06 (d, *J *= 8.8 Hz, 2H, PMB), 6.80 (d, *J *= 8.8 Hz, 2H, PMB), 5.33–5.27 (m, 1H, H-1), 5.12 (d, *J *= 4.0 Hz, 1H, H-1′), 4.81–4.75 (m, 3H, C*H*_2_Ph), 4.67 (d, 1H, *J *= 1.6 Hz, H-5), 4.65–4.61 (m, 3H, C*H*_2_Ph), 4.6 (br s, 1H, H-2), 4.51–4.48 (m, 2H, C*H*_2_Ph), 4.28–4.27 (m, 1H, H-3), 4.21–4.20 (m, 1H, H-4), 3.93–3.90 (m, 1H, H-3′), 3.82–3.77 (m, 2H, H-6′), 3.67–3.60 (m, 3H, H-2′, H-4′, H-5′), 3.74 (s, 3H, PMBOC*H*_3_), 3.42 (s, 3H, OC*H*_3_); ^13^C NMR (100 MHz; CD_3_OD) *δ* 175.7, 160.7, 139.4, 139.0, 131.6, 130.5, 129.4, 129.3, 129.2, 129.0, 128.8, 128.7, 128.6, 128.6, 114.6, 95.2, 82.7, 78.8, 76.5, 75.4, 74.4, 72.9, 72.9, 72.4, 71.2, 71.2, 71.1, 71.1, 70.9, 70.9, 69.3, 65.3, 55.6; FTMS (ESI^−^) *m*/*z* calcd for C_42_H_46_N_3_O_15_S_1_ [M−2Na+H]^−^ 864.2655, found 864.2665.

#### 5.1.9 Methyl 6-*O*-sulfonato-α-d-glucosaminopyranosyl-(1→4)-2-*O*-sulfonato-α-l-idopyranoside uronate tri sodium salt **14**

Disaccharide **12** (195 mg, 0.21 mmol) was dissolved in MeOH (2 mL), THF (0.5 mL) and H_2_O (1 mL). Pd(OH)_2_/C (200 mg) was added and the system purged with H_2_. Stirring was continued at rt for 2 d. The suspension was then filtered through Celite® and solvents removed in vacuo to reveal **14** (126 mg, 0.21 mmol, quant.) as a clear glass. ^1^H NMR (400 MHz; D_2_O): *δ* 5.04 (d, *J *= 3.6 Hz 1H, H1′), 4.98 (s, 1H, H_1_), 4.48 (d, *J *= 1.8 Hz, 1H, H-5), 4.32 (dd, *J *= 11.1, 2.6 Hz, 1H, H-6A′), 4.23 (s, 1H, H-3), 4.18 (s, 1H, H-2), 4.14 (dd, *J *= 11.1, 2.1 Hz, 1H, H-6B′), 4.04 (s, 1H, H-4), 3.90 (dt, *J *= 10.2, 2.2 Hz, 1H, H-5′), 3.66 (t, *J *= 9.8 Hz, 1H, H-3′), 3.47 (t, *J *= 9.7 Hz, 1H, H4′), 3.38 (s, 3H, OCH_3_), 2.89 (dd, *J *= 10.3, 3.2 Hz, 1H, H-2′); FTMS (ESI^−^) *m*/*z* calcd for C_13_H_22_NO_17_S_2_ [M−3Na+2H]^−^ 528.0335, found 528.0335. Other analytical data matched those previously reported.^23^

#### 5.1.10 Methyl α--d-glucosaminopyranosyl-(1→4)-2-*O*-sulfonato-α-l-idopyranoside uronate di sodium salt **15**

Disaccharide **13** (107 mg, 0.12 mmol) was dissolved in MeOH/H_2_O (3 mL/1 mL). To the flask was attached a 3-way tap and the system purged with N_2_. 10–20% Pd(OH)_2_/C (100 mg) was added and the system purged with H_2_. Vigorous stirring having a balloon with H_2_ attached (1 atm) was continued at rt for 36 h. The suspension was then filtered through Celite® and solvents removed in vacuo to reveal **15** (56 mg, 0.11 mmol, 98%) as a clear glass. *R_f_ *= 0.25 (EtOAc/pyridine/H_2_O/AcOH 6:5:3:1); [*α*]_D_^20^ = +49.1 (*c* 0.50, H_2_O); ^1^H NMR (400 MHz; D_2_O) *δ* 5.15 (d, *J *= 3.6 Hz, 1H, H1′), 4.97 (s, 1H, H-1), 4.46 (d, *J *= 1.7 Hz, 1H, H-5), 4.223–4.22 (m, 1H, H-3), 4.17 (ddd, *J *= 3.6, 2.4, 1.4 Hz, 1H, H-2), 4.03 (br s, 1H, H-4), 3.75–3.71 (m, 3H, H-5′, H-6A′, H-6B′), 3.67 (dd, *J *= 10.4, 9.3 Hz, 1H, H-3′), 3.41–3.36 (m, 4H, H4′, OC*H*_3_), 2.92 (dd, *J *= 10.4, 3.6 Hz, 1H, H-2′); ^13^C NMR (100 MHz; D_2_O) *δ* 175.3, 99.1, 94.2, 72.5, 71.9, 71.6, 71.1, 69.3, 66.2, 63.5, 59.9, 55.3, 54.6; ES MS 448.1 (M−2Na+H)^−^. Other analytical data matched those previously reported.^23^

#### 5.1.11 Methyl 6-*O*,*N*-di-sulfonato-α-d-glucosaminopyranosyl-(1→4)-2-*O*-sulfonato-α-l-idopyranoside uronate tetra sodium salt **16**

Disaccharide **14** (126 mg, 0.21 mmol) was dissolved in H_2_O (3 mL) at rt NaHCO_3_ (176 mg, 2.1 mmol) and SO_3_·pyr (167 mg, 1.05 mmol) were then added and the suspension stirred at room temperature. Further additions of both these reagents were made at 2 h and 4 h and stirring continued overnight. TLC analysis (EtOAc/Pyr/H_2_O/AcOH; 6:5:3:1) showed no starting material remained and a new species had formed on the baseline. Solvent was removed in vacuo to reveal a crude white solid which was purified by passage through a Sephadex G-20 resin using water as eluent. The target material **16** (115 mg, 0.16 mmol, 79%) was isolated as a white solid. ^1^H NMR (400 MHz; D_2_O) *δ* 5.29 (d, *J *= 3.5 Hz, 1H, H1′), 5.03 (s, 1H, H-1), 4.43 (d, *J *= 2.3 Hz, 1H, H-5), 4.33 (dd, *J *= 11.1, 2.3 Hz, 1H, H-6A′), 4.20 (t, *J *= 1.5 Hz, 2H, H-2, H-3), 4.16 (dd, *J *= 11.1, 2.1 Hz, 1H, H-6B′), 4.02–4.00 (m, 1H, H-4), 3.93 (dt, *J *= 10.0, 2.1 Hz, 1H, H-5′), 3.64 (dd, *J *= 10.3, 9.2 Hz, 1H, H-3′), 3.54 (dd, *J *= 10.0, 9.3 Hz, 1H, H4′), 3.38 (s, 3H, OCH_3_), 3.20 (dd, *J *= 10.3, 3.5 Hz, 1H, H-2′); ^13^C NMR (100 MHz; D_2_O) *δ* 174.9, 99.5, 97.4, 75.6, 74.6, 70.9, 69.9, 69.1, 67.49, 67.40, 66.4, 57.9, 55.4; FTMS (ESI^−^) *m*/*z* calcd for C_13_H_19_NNa_3_O_20_S_3_ [M−Na]^−^ 673.9349, found 673.9361. Other analytical data matched previously reported.^23^

#### 5.1.12 Methyl *N*-sulfonato-α-d-glucosaminopyranosyl-(1→4)-2-*O*-sulfonato-α-l-idopyranoside uronate tri sodium salt **17**

Disaccharide **15** (40 mg, 0.08 mmol) was dissolved in H_2_O (3 mL), NaHCO_3_ (41 mg, 0.49 mmol) and sulfurtrioxide·pyridine complex (39 mg, 0.25 mmol) were then added and the suspension stirred at room temperature. Further additions of both these reagents (same amounts) were made after 1, 3, 8 and 16 h and stirring continued for another 8 h. Solvent was removed in vacuo to reveal a crude white solid which was purified by passage through Sephadex G-20 size exclusion resin using water as eluent. The target material **17** (44 mg, 0.07 mmol, 92%) was isolated as a white solid. [*α*]_D_^20^ = +37.0 (*c* 0.70, H_2_O); ^1^H NMR (400 MHz; D_2_O) *δ* 5.27 (d, *J *= 3.5 Hz, 1H, H1′), 5.04 (s, 1H, H-1), 4.43 (d, *J *= 2.2 Hz, 1H, H-5), 4.22–4.20 (m, 2H, H-2, H-3), 4.00 (br s, 1H, H-4), 3.81–3.74 (m, 3H, H-5′, H-6A′, H-6B′), 3.63 (dd, *J *= 10.5, 9.2 Hz, 1H, H-3′), 3.46–3.42 (m, 1H, H4′), 3.38 (s, 3H, OC*H*_3_), 3.18 (dd, *J *= 10.5, 3.5 Hz, 1H, H-2′); ^13^C NMR (100 MHz; D_2_O) *δ* 175.2, 99.4, 97.3, 75.5, 74.4, 71.7, 70.9, 69.8, 67.3, 60.1, 58.0, 55.4; ES MS 550.0 (M−2Na+H)^−^. Other analytical data matched those previously reported.^23^

#### 5.1.13 Methyl-α-d-glucosaminopyranosyl-(1→4)-α-l-idopyranoside uronate mono sodium salt **18**

Disaccharide **11** (75 mg, 0.20 mmol) was dissolved in EtOH/H_2_O (5 mL/0.5 mL) and NaHCO_3_ (12 mg, 0.20 mmol) added. To the flask was attached a 3-way tap and purged with N_2_, 10–20% Pd(OH)_2_/C (53 mg) was added and the system purged with H_2_. Vigorous stirring having a balloon with H_2_ attached (1 atm.) was continued at 50 °C for 32 h. The suspension was then filtered through Celite® and solvents removed in vacuo to reveal **18** (42 mg, 0.11 mmol, 95%) as a clear glass. [*α*]_D_^20^ = +52.2 (*c* 1.15, H_2_O); ^1^H NMR (400 MHz; D_2_O) *δ* 5.01 (d, *J *= 3.6 Hz, 1H, H1′), 4.62 (d, *J *= 2.9 Hz, 1H, H-1), 4.33 (d, *J *= 2.7 Hz, 1H, H-5), 3.91 (t, *J *= 3.0 Hz, 1H, H-4), 3.86 (t, *J *= 4.3 Hz, 1H, H-3), 3.66–3.64 (m, 3H, H-5′, H-6A′, H-6B′), 3.50–3.43 (m, 2H, H-2, H-3′), 3.28–3.24 (m, 4H, H4′, OC*H*_3_), 2.60 (dd, *J *= 10.2, 3.6 Hz, 1H, H-2′); ^13^C NMR (100 MHz; D_2_O) *δ* 175.2, 101.6, 96.4, 74.0, 73.2, 72.1, 69.6, 68.9, 68.60, 68.49, 60.2, 55.3, 55.0. Other analytical data matched those previously reported.^23^

## Supplementary data

### MOL files

The following ZIP file contains the MOL files of the most important compounds referred to in this article.

MOL files ZIP file containing the MOL files of the most important compounds in this article.
