# Supplementary material for: Synthesis of a heparin-related GlcN–IdoA sulfation-site variable disaccharide library and analysis by Raman and ROA spectroscopy
Source: Carbohydr Res. 2014 Dec 5;400:44–53. doi: 10.1016/j.carres.2014.06.026 (PMC4245711; doi:10.1016/j.carres.2014.06.026)

SUPPORTING INFORMATION

Synthesis of a heparin-related GlcN-IdoA sulfation-site variable disaccharide library and analysis by Raman and ROA spectroscopy

Gavin J. Miller,[a] Steen U. Hansen,[a] Marek Baráth,[a$] Christian Johannessen,[b^] Ewan Blanch,[b] Gordon C. Jayson[c] and John M. Gardiner[a*]

[a]*Manchester Institute of Biotechnology and School of Chemistry, 131 Princess Street, University of Manchester M1 7DN, UK.*
[b]*Manchester Institute of Biotechnology, 131 Princess Street, University of Manchester M1 7DN, UK.*
[c]*School of Cancer and Enabling Sciences, Wilmslow Road, University of Manchester UK, M20 4BX.*
**Corresponding author. Email: gardiner@manchester.ac.uk*

1. General Experimental

All the chemicals used were purchased from commercial sources without further purification. Heparin was purchased from Sigma-Aldrich and used without further purification. All reactions were monitored by TLC on Merck silica gel plates $^{60}F_{254}$. Silica gel 60 (particle size 0.035-0.070 mm) was used for column chromatography. ^{1}H NMR spectra were recorded at 400 MHz and ^{13}C spectra at 100 MHz on Bruker DPX spectrometers. Mass spectra (MS) were recorded using a Micromass Platform II spectrometer using an electrospray ionization source or via the EPSRC National Mass Spectrometry Service (Swansea). Optical rotations were obtained using an AA-1000 polarimeter. Elemental analyses were performed by Micro Analytical Laboratory, School of Chemistry, The University of Manchester. NMR Data were reprocessed using iNMR 4 from Nucleomatica and Topspin.

2. ^{1}H, ^{13}C NMR and COSY Spectral Data for compounds 4-18

Methyl 6-*O*-acetyl-2-azido-3-*O*-benzyl-2-deoxy-4-*O*-*p*-methoxybenzyl-α-D-glucopyranosyl-(1⟶4)-(methyl 2-*O*-benzoyl-3-*O*-benzyl-α-L-idopyranoside) uronate 4

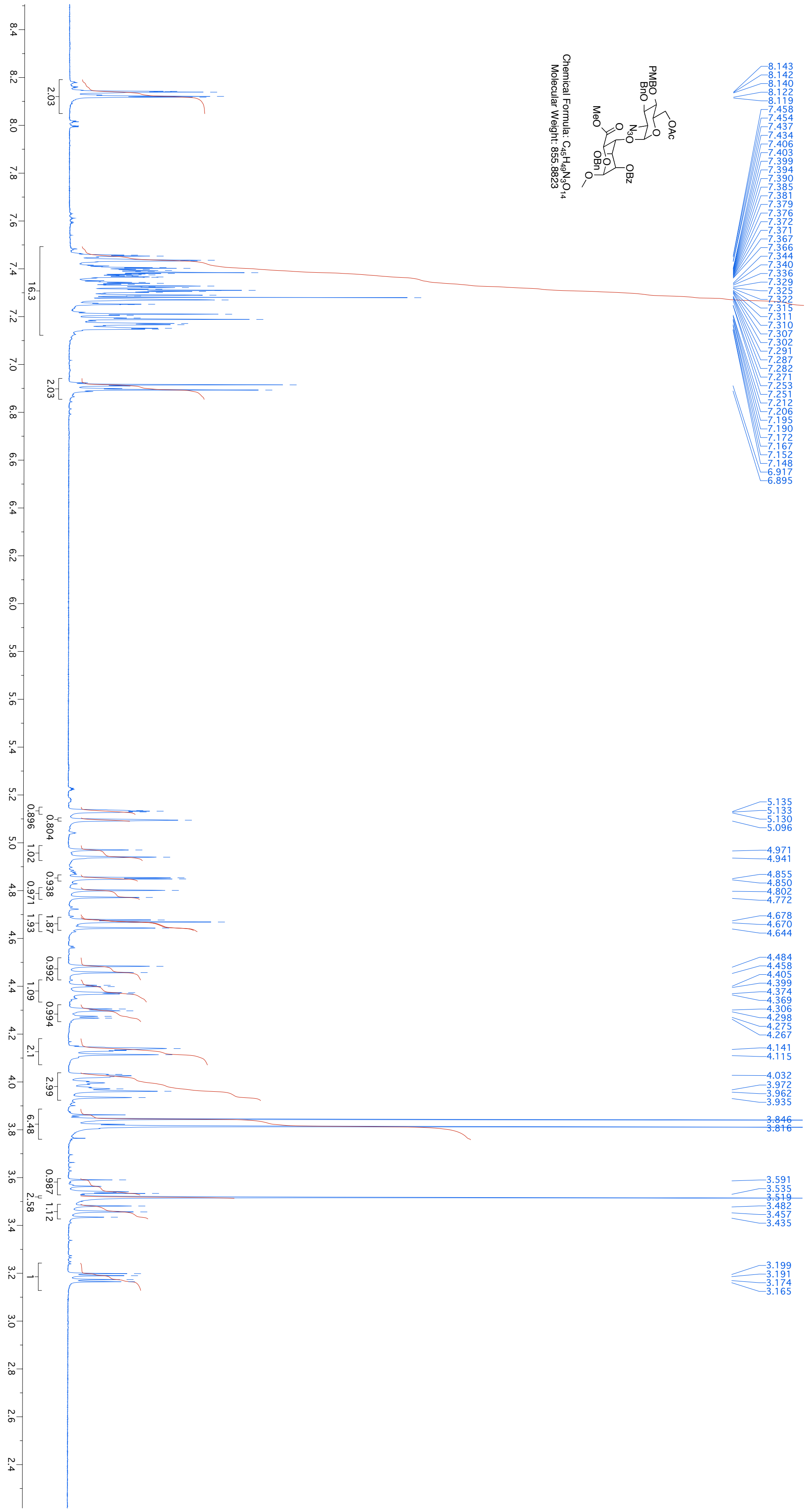

Methyl 6-*O*-acetyl-2-azido-3-*O*-benzyl-2-deoxy-4-*O*-*p*-methoxybenzyl-α-D-glucopyranosyl-(1⟶4)-(methyl 2-*O*-benzoyl-3-*O*-benzyl-α-L-idopyranoside) uronate 4

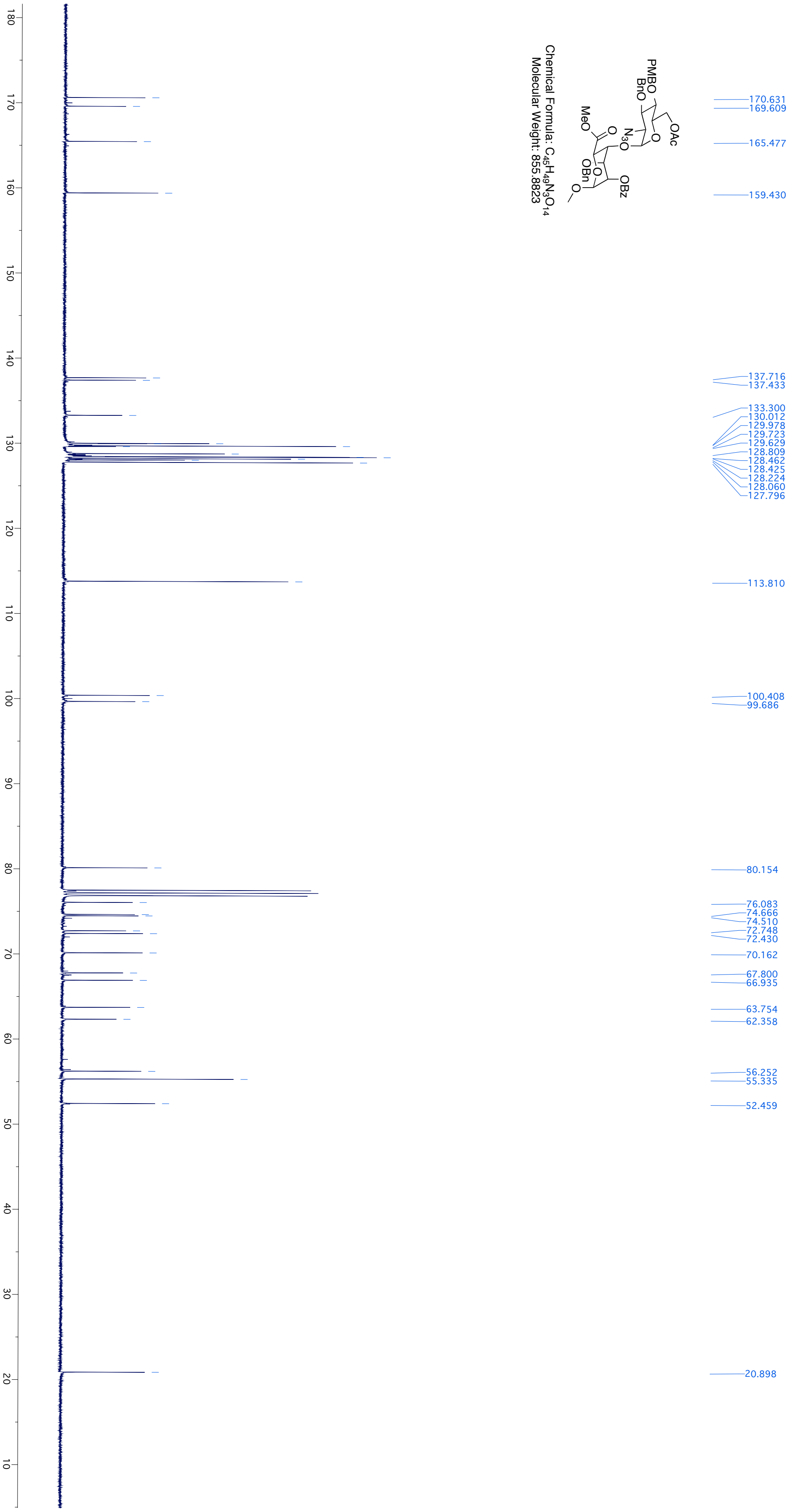

Methyl 6-*O*-acetyl-2-azido-3-*O*-benzyl-2-deoxy-4-*O*-*p*-methoxybenzyl-α-D-glucopyranosyl-(1⟶4)-(methyl 2-*O*-benzoyl-3-*O*-benzyl-α-L-idopyranoside) uronate 4

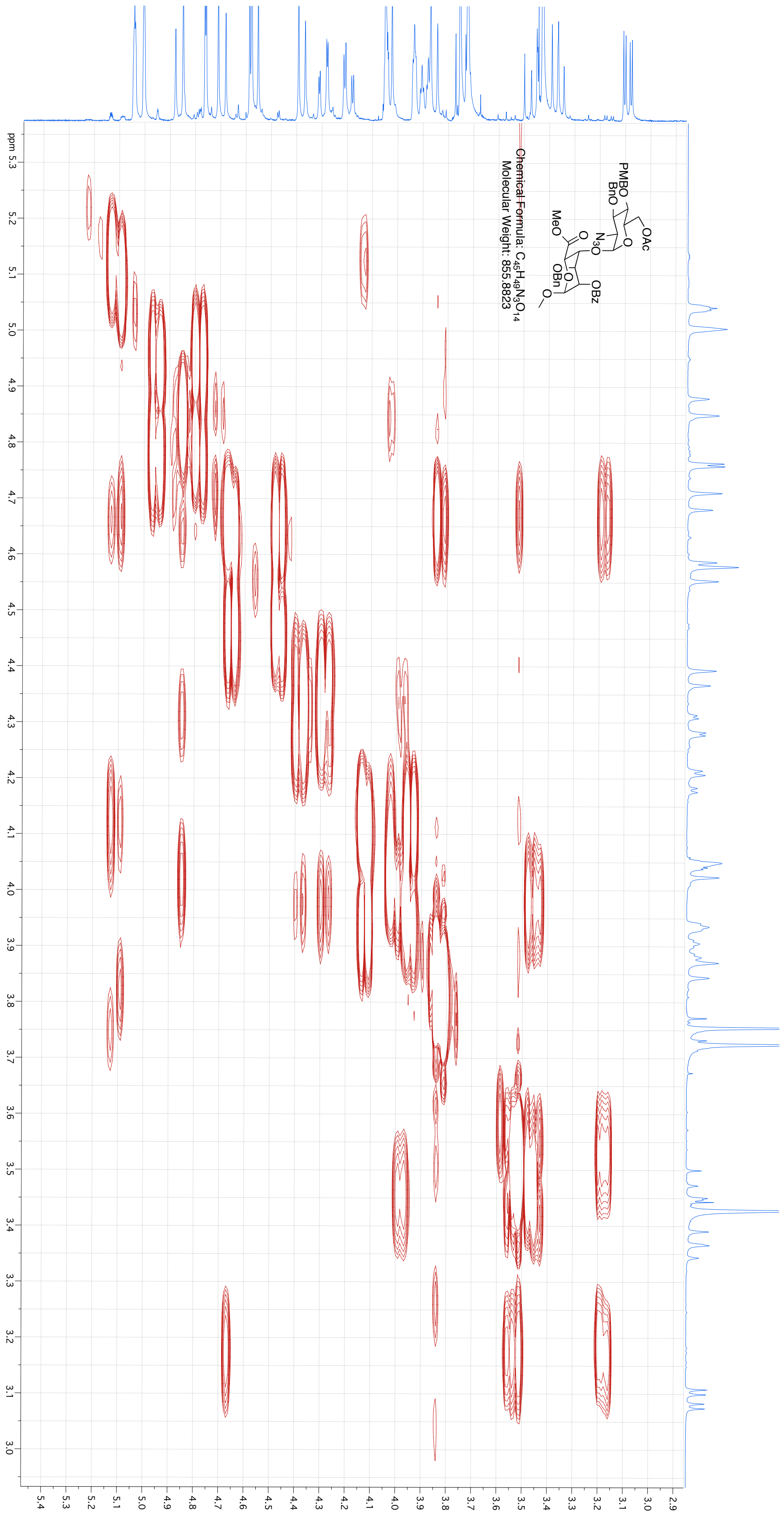

Methyl 2-azido-3,6-di-O-benzyl-2-deoxy-4-O-p-methoxybenzyl-α-D-glucopyranosyl-(1⟶4)-(methyl 2-O-benzoyl-3-O-benzyl-α-L-idopyranoside) uronate 6

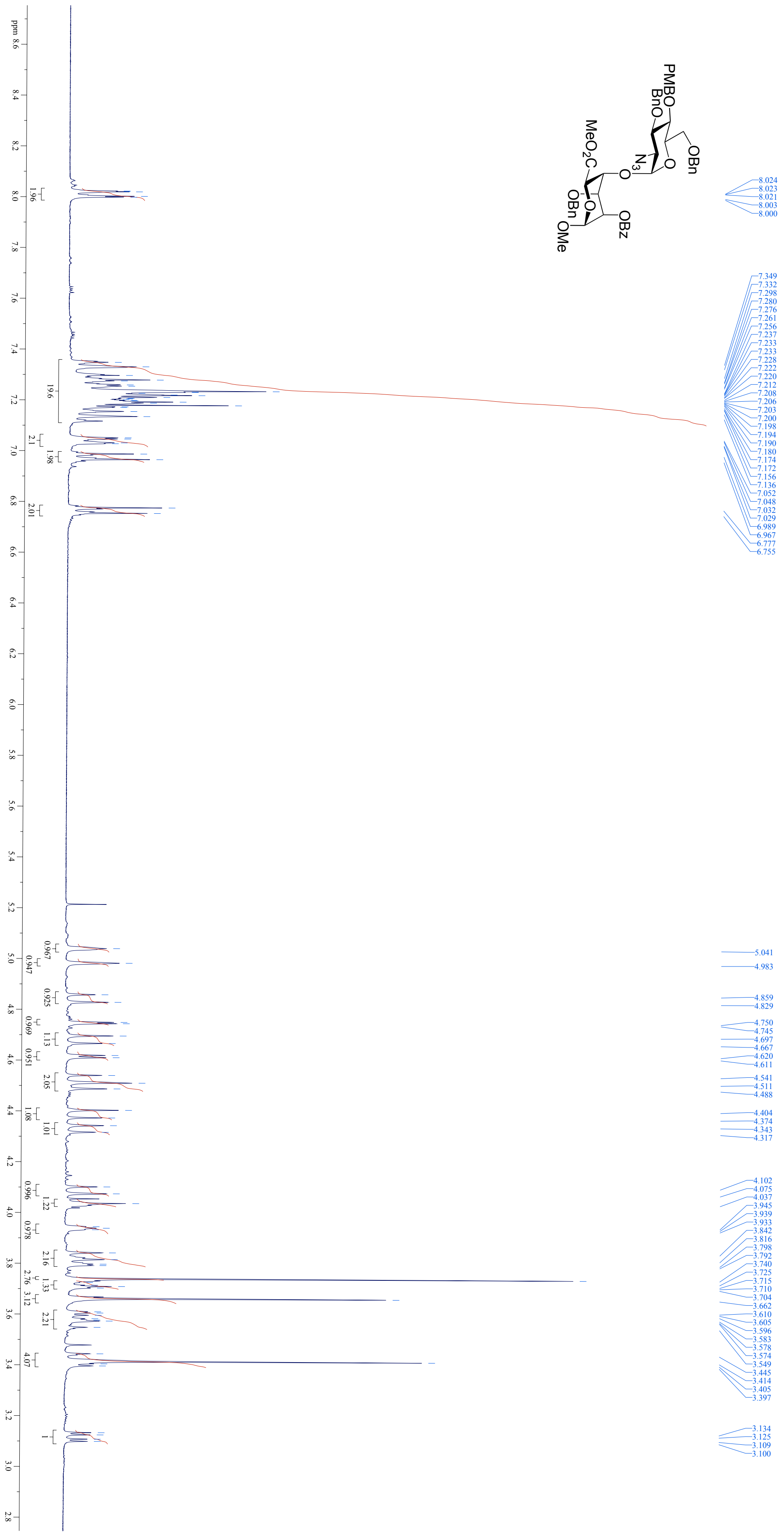

Methyl 2-azido-3,6-di-O-benzyl-2-deoxy-4-O-p-methoxybenzyl-α-D-glucopyranosyl-(1⟶4)-(methyl 2-O-benzoyl-3-O-benzyl-α-L-idopyranoside) uronate 6

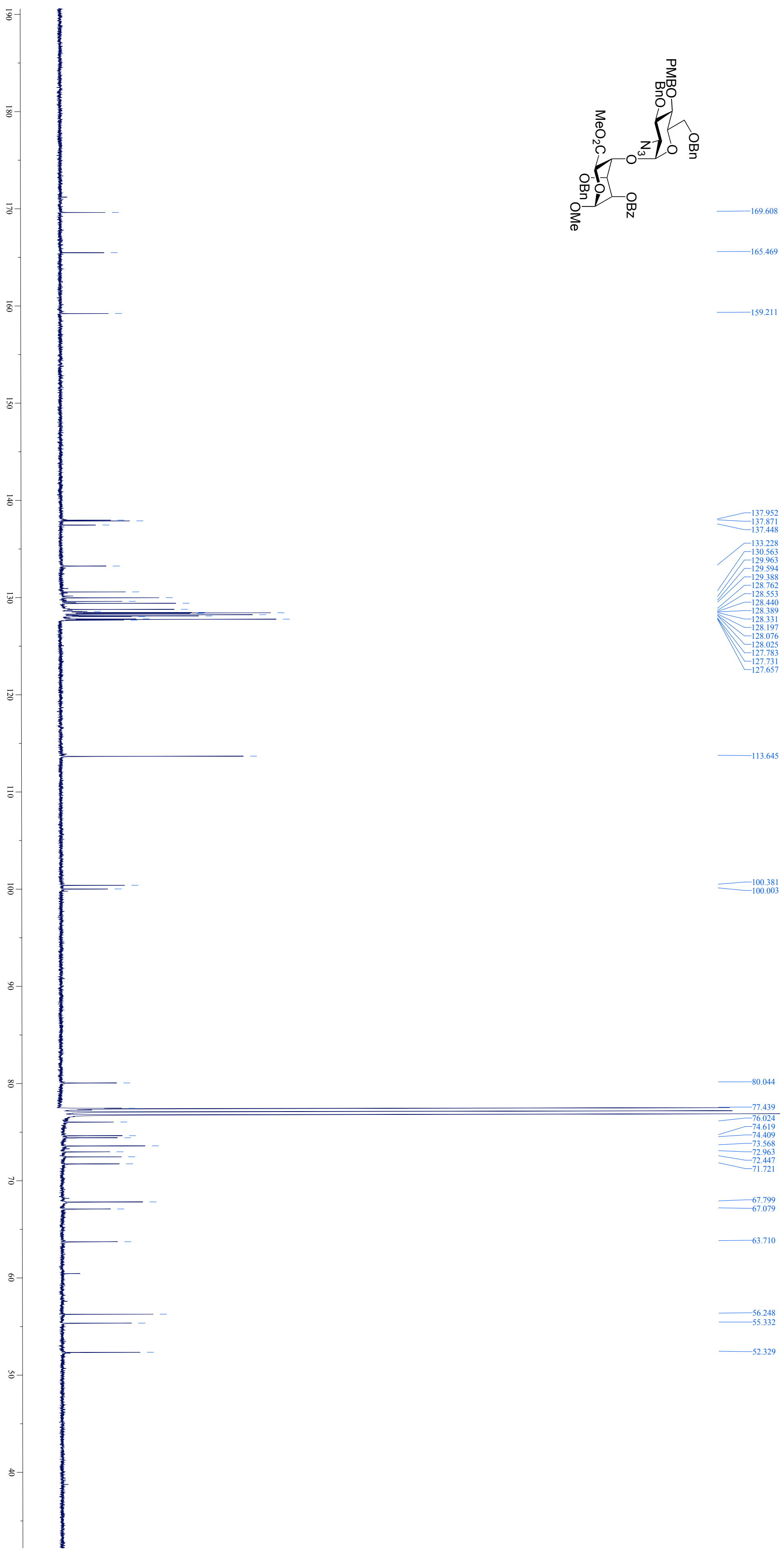

Methyl 2-azido-3,6-di-O-benzyl-2-deoxy-4-O-p-methoxybenzyl-α-D-glucopyranosyl-(1⟶4)-(methyl 2-O-benzoyl-3-O-benzyl-α-L-idopyranoside) uronate 6

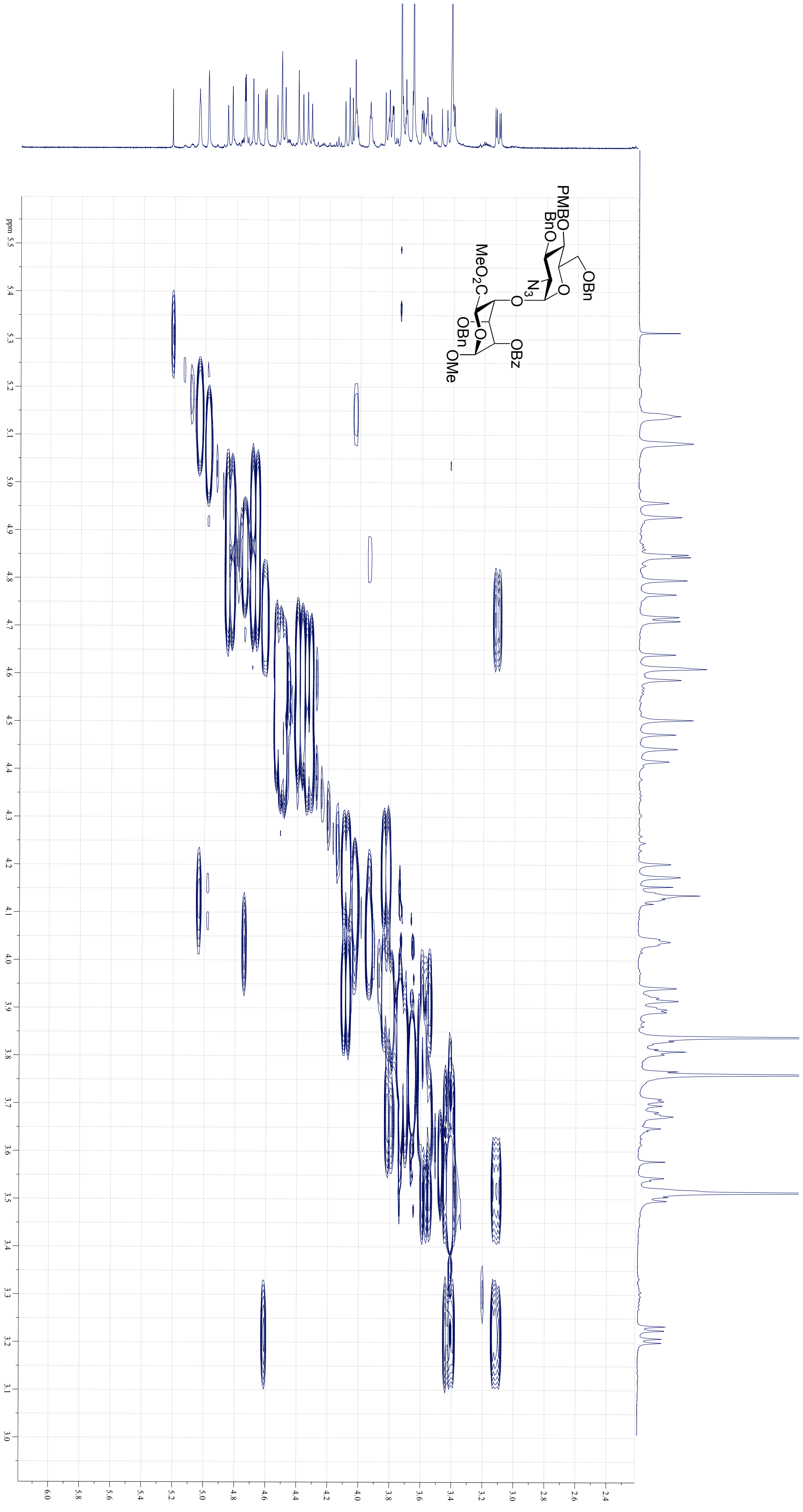

Methyl 2-azido-3-*O*-benzyl-4,6-*O*-benzylidene-2-deoxy-α-D-glucopyranosyl-(1⟶4)-(methyl 2-*O*-benzoyl-3-*O*-benzyl-α-L-idopyranoside) uronate 8

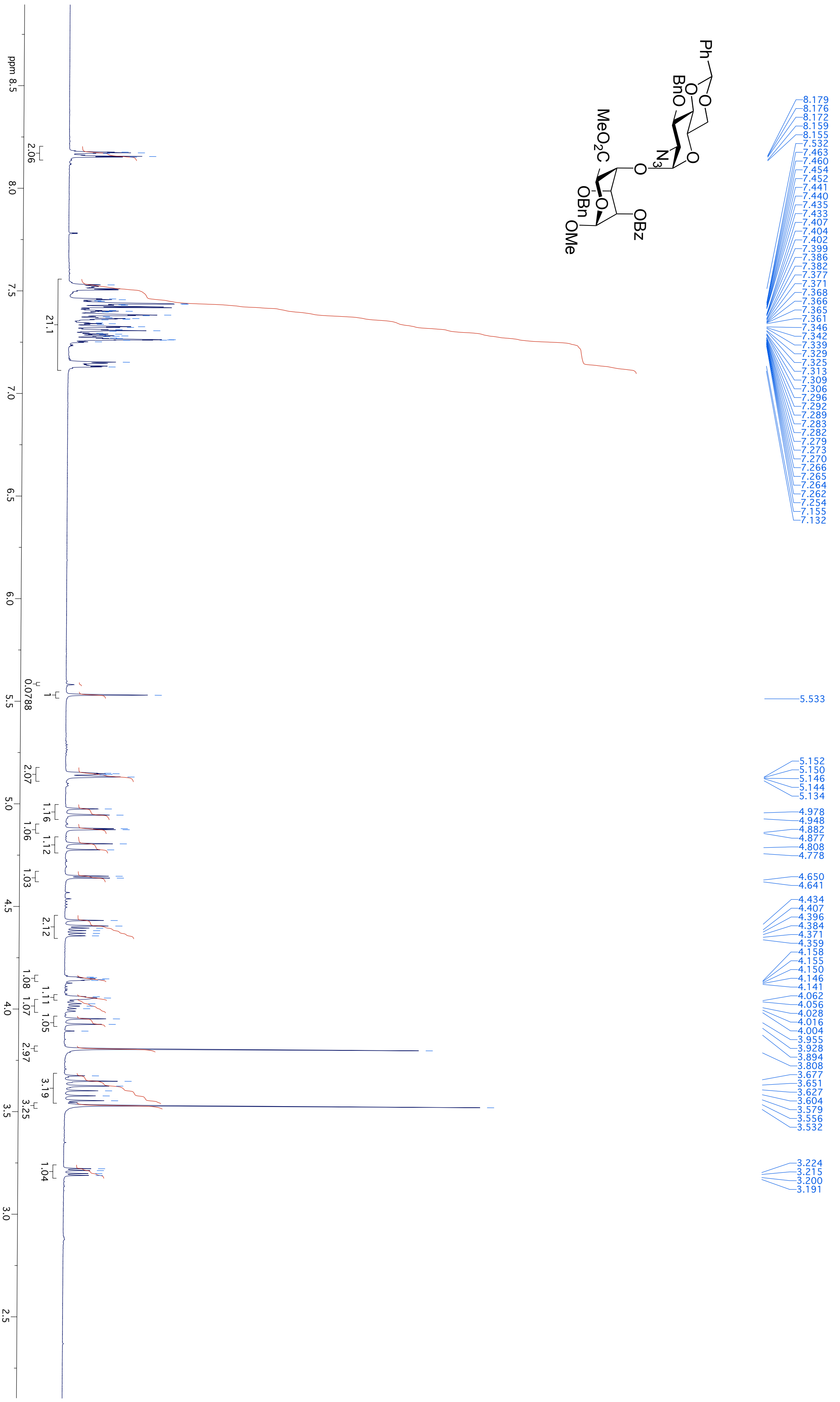

Methyl 2-azido-3-*O*-benzyl-4,6-*O*-benzylidene-2-deoxy-α-D-glucopyranosyl-(1⟶4)-(methyl 2-*O*-benzoyl-3-*O*-benzyl-α-L-idopyranoside) uronate 8

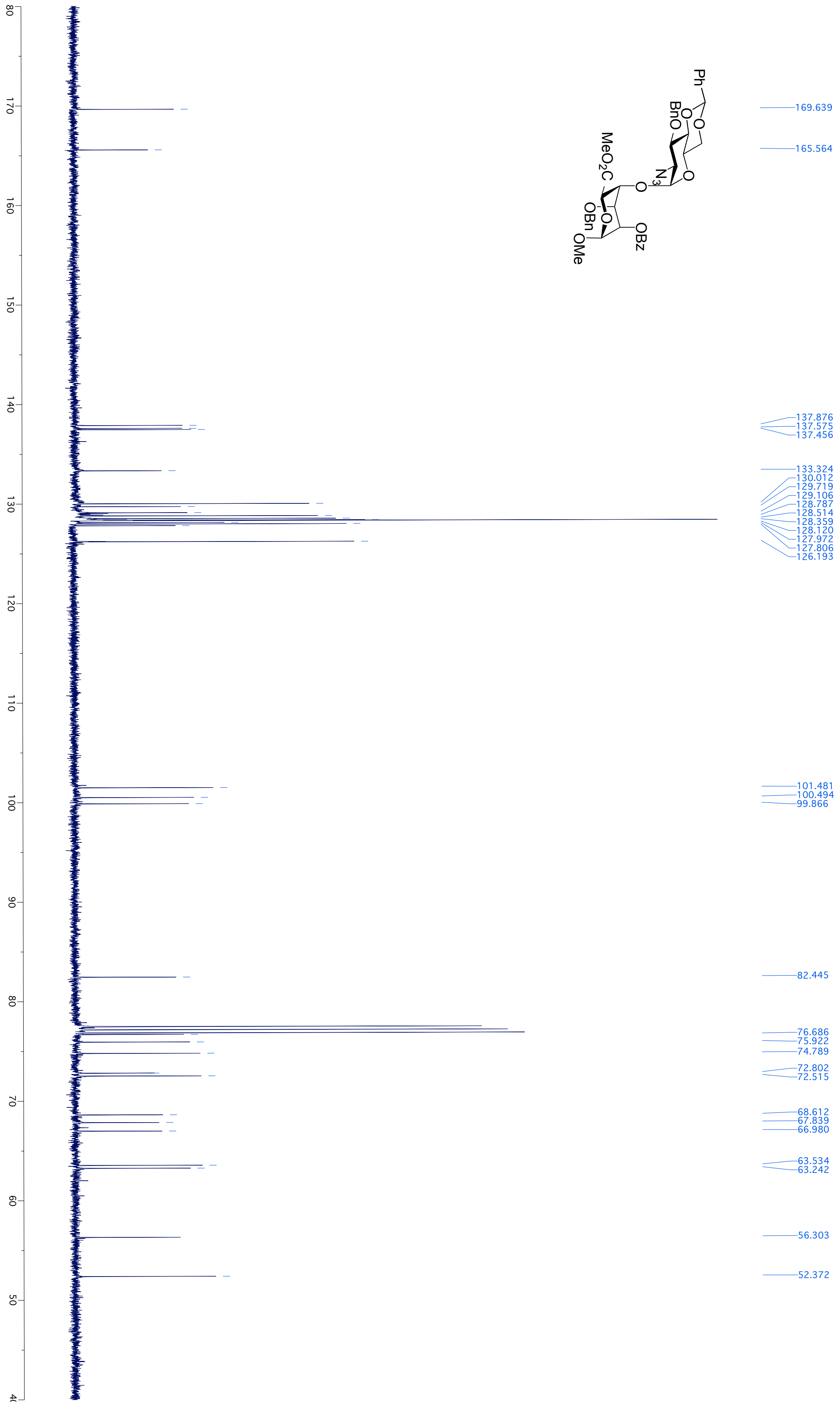

Methyl 2-azido-3-*O*-benzyl-4,6-*O*-benzylidene-2-deoxy-α-D-glucopyranosyl-(1⟶4)-(methyl 2-*O*-benzoyl-3-*O*-benzyl-α-L-idopyranoside) uronate 8

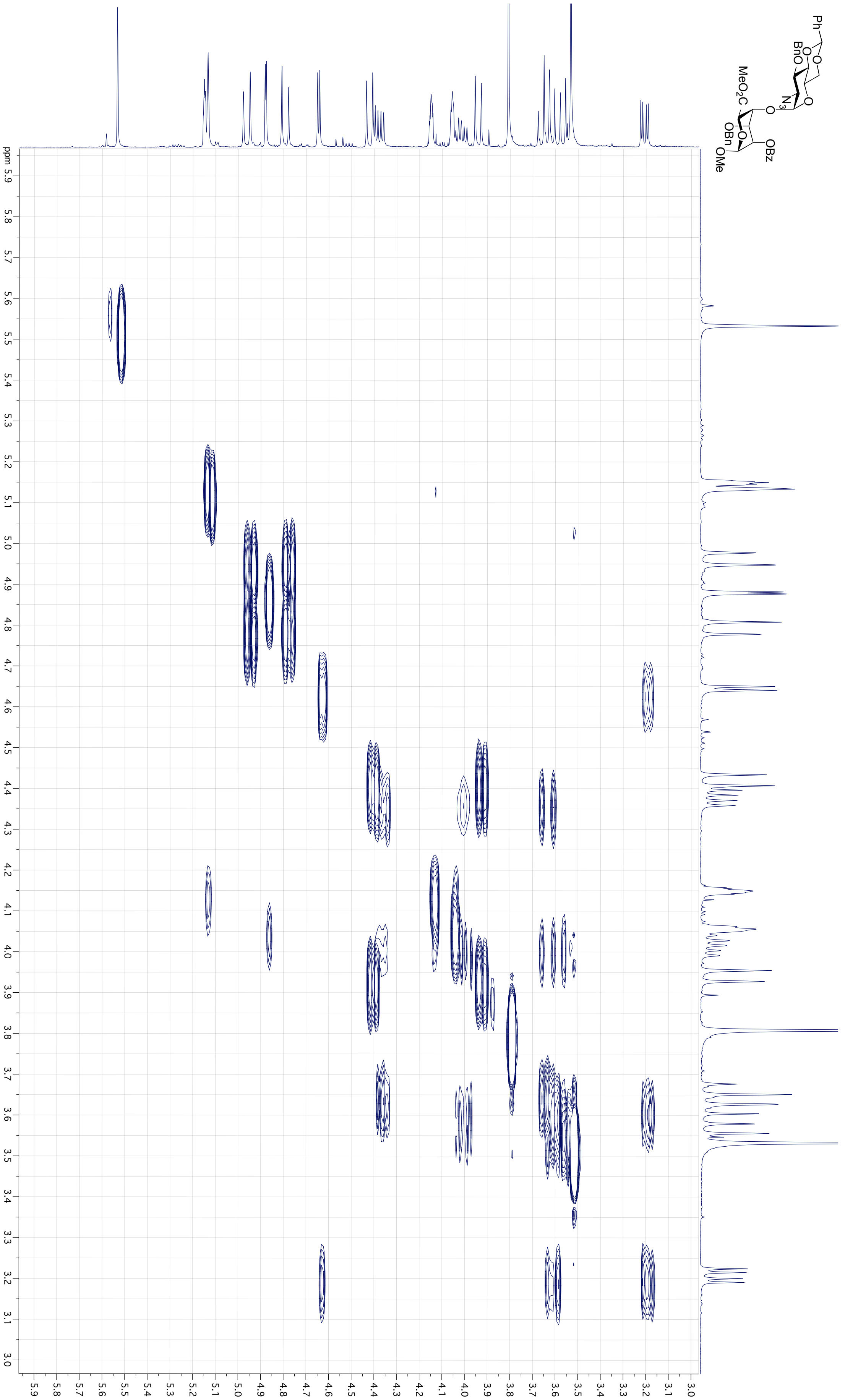

2-Azido-3-*O*-benzyl-2-deoxy-4-*O*-*p*-methoxybenzyl-α-D-glucopyranosyl-(1⟶4)-(methyl 3-*O*-benzyl-α-L-idopyranoside) uronic acid 9

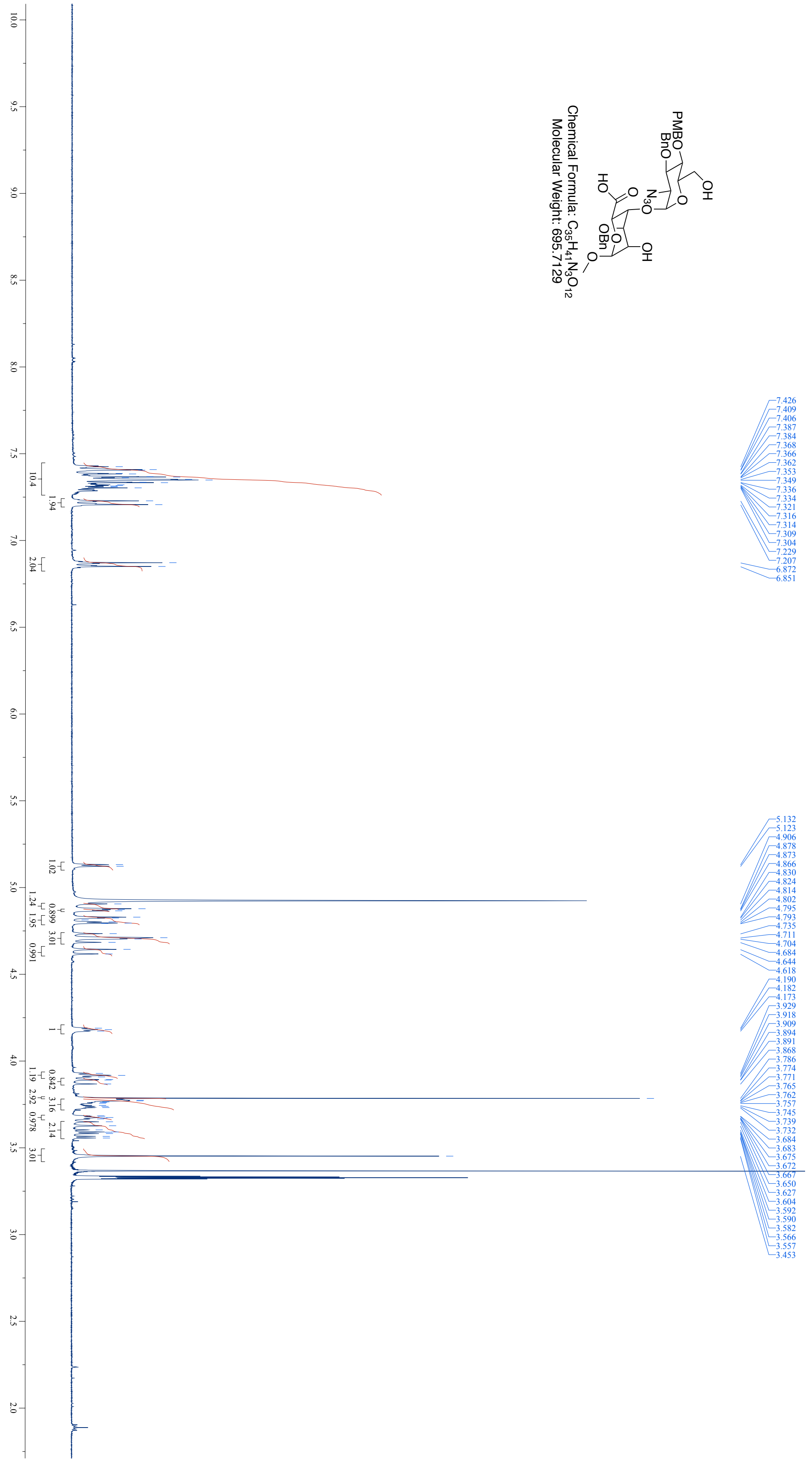

2-Azido-3-*O*-benzyl-2-deoxy-4-*O*-*p*-methoxybenzyl-α-D-glucopyranosyl-(1⟶4)-(methyl 3-*O*-benzyl-α-L-idopyranoside) uronic acid 9

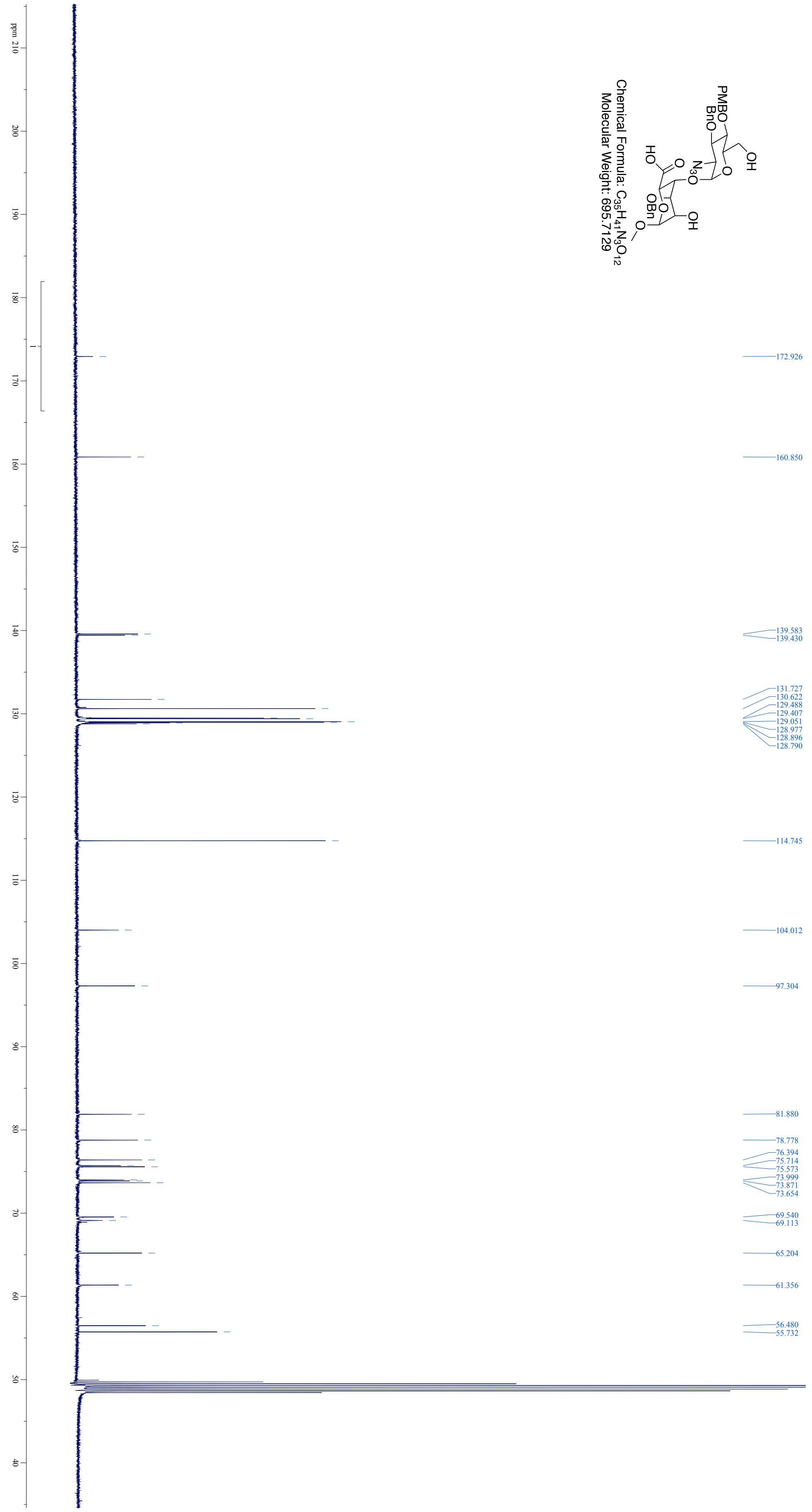

2-Azido-3-*O*-benzyl-2-deoxy-4-*O*-*p*-methoxybenzyl-α-D-glucopyranosyl-(1⟶4)-(methyl 3-*O*-benzyl-α-L-idopyranoside) uronic acid 9

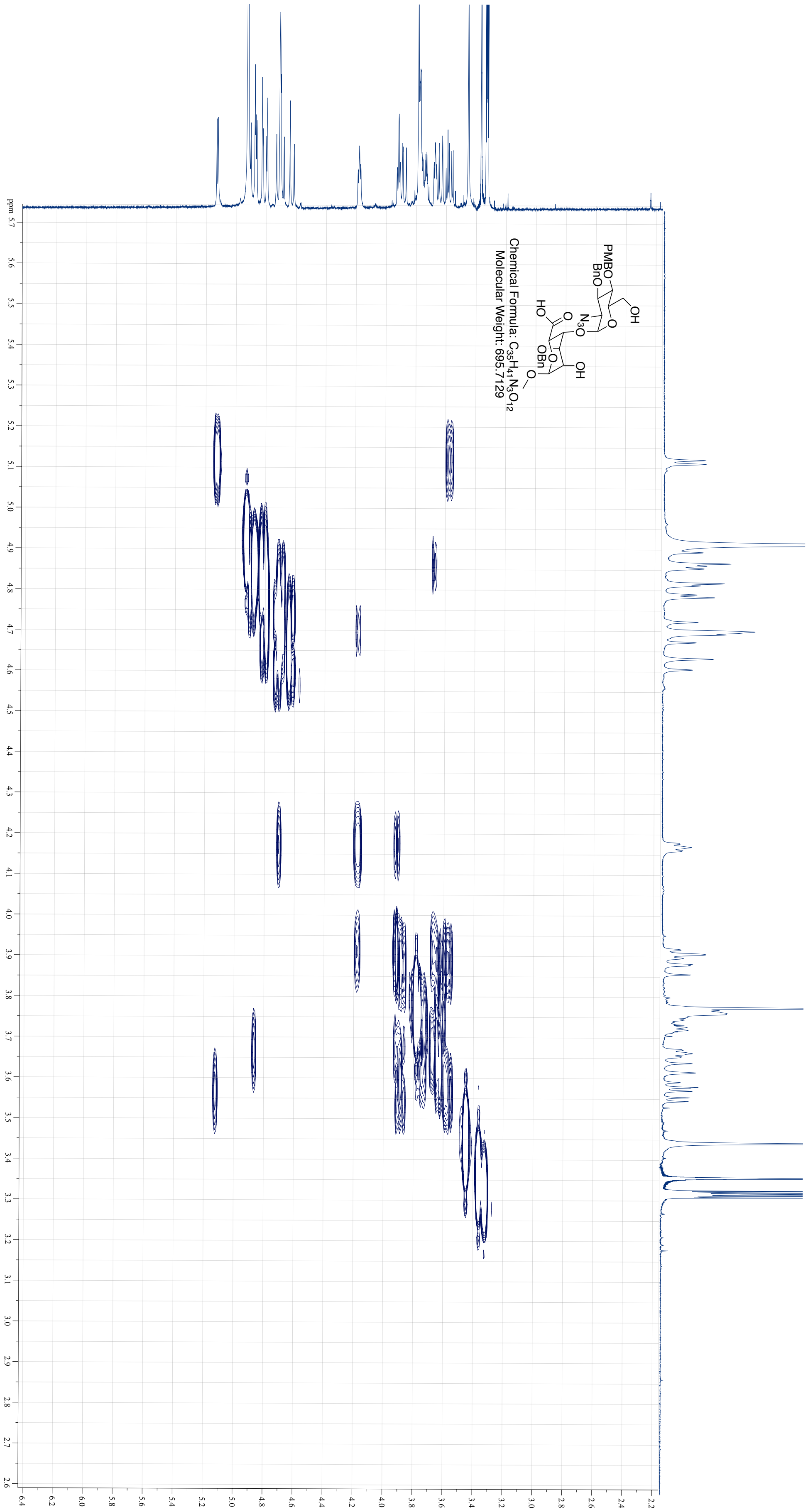

2-azido-3,6-di-*O*-benzyl-2-deoxy-4-*O*-*p*-methoxybenzyl-α-D-glucopyranosyl-(1⟶4)-(methyl 3-*O*-benzyl-α-L-idopyranoside) uronic acid 10

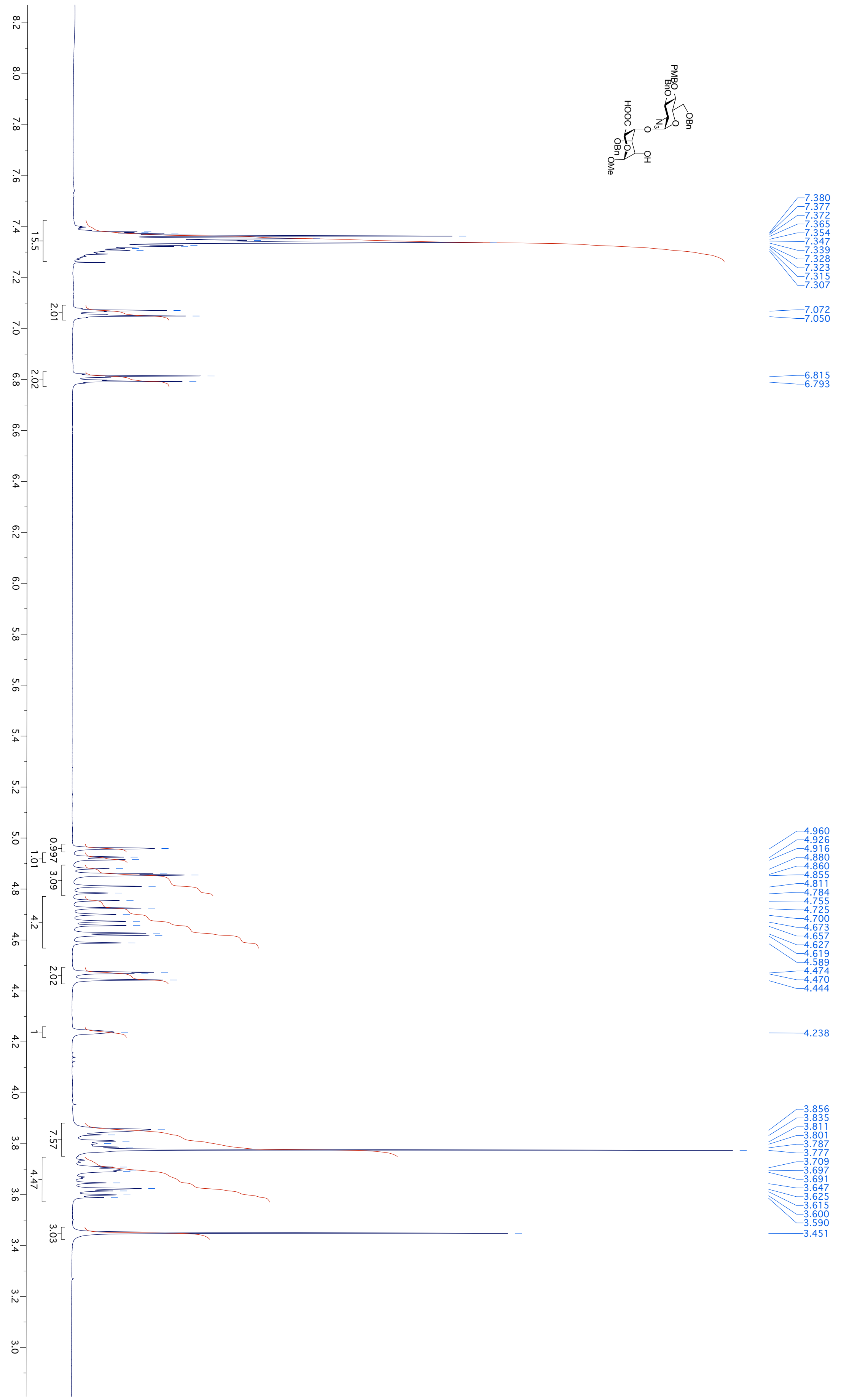

2-azido-3,6-di-*O*-benzyl-2-deoxy-4-*O*-*p*-methoxybenzyl-α-D-glucopyranosyl-(1⟶4)-(methyl 3-*O*-benzyl-α-L-idopyranoside) uronic acid 10

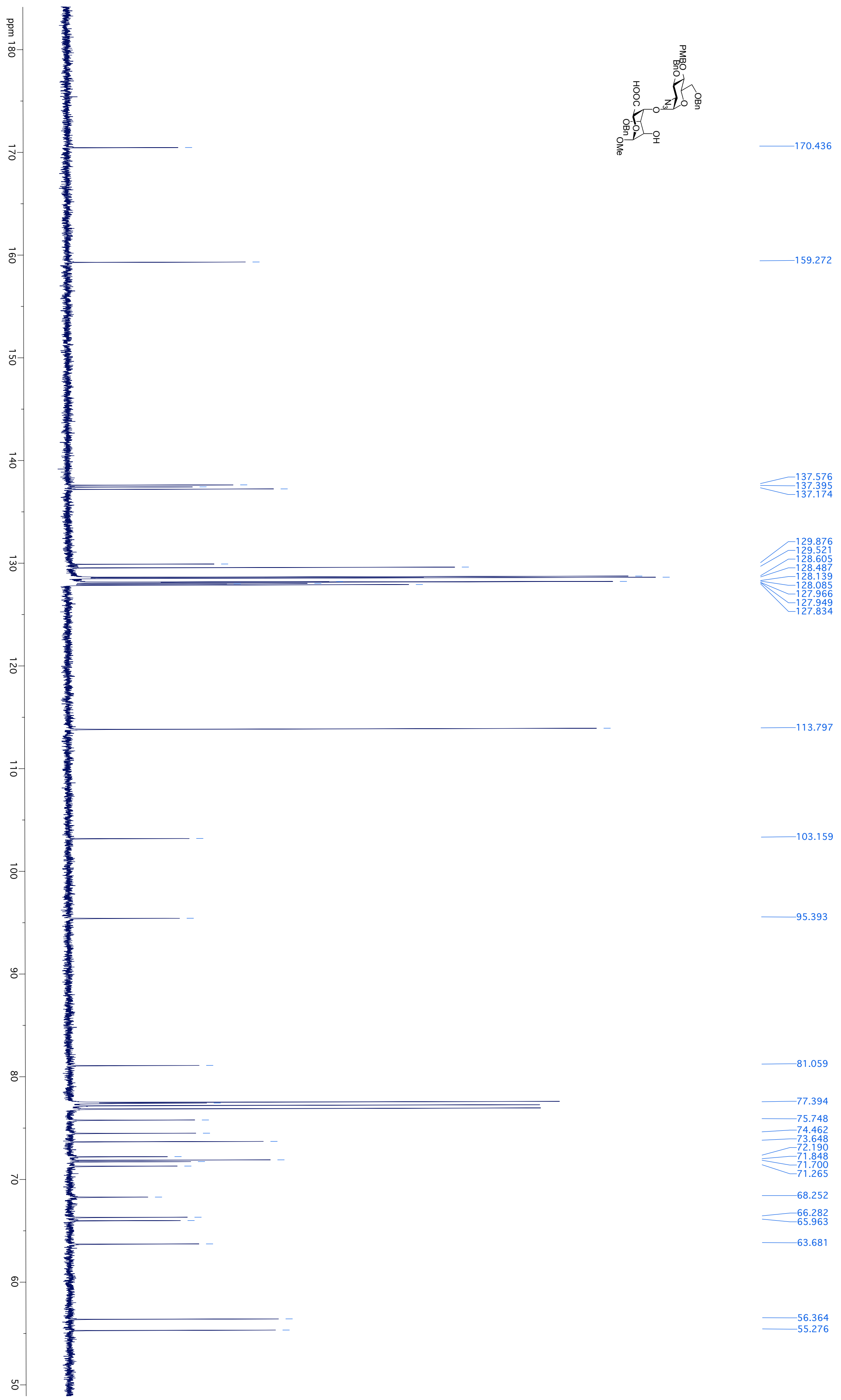

2-azido-3,6-di-*O*-benzyl-2-deoxy-4-*O*-*p*-methoxybenzyl-α-D-glucopyranosyl-(1⟶4)-(methyl 3-*O*-benzyl-α-L-idopyranoside) uronic acid 10

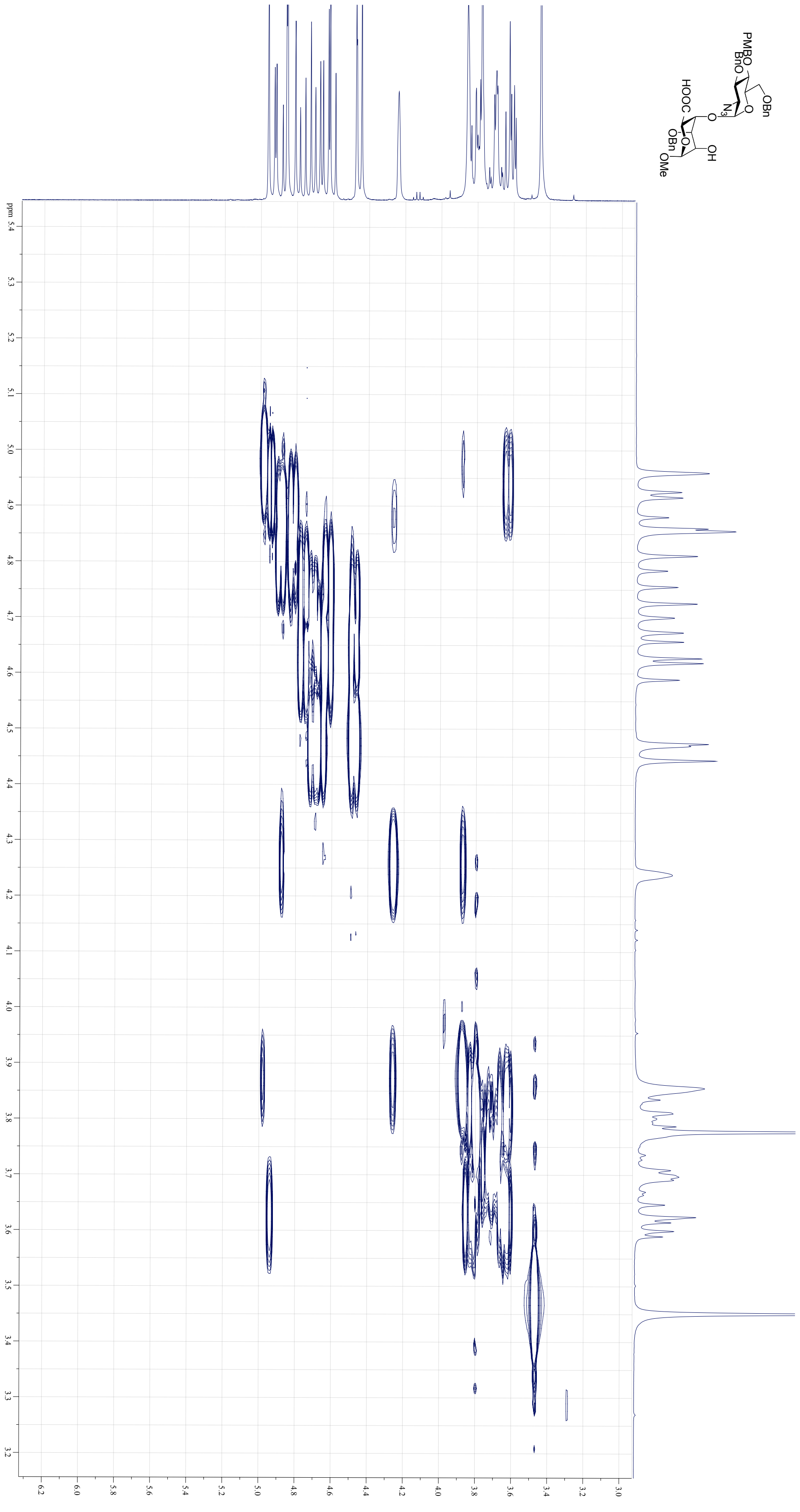

2-azido-3-*O*-benzyl-4,6-*O*-benzylidene-2-deoxy-α-D-glucopyranosyl-(1⟶4)-(methyl 3-*O*-benzyl-β-L-idopyranoside) uronic acid 11

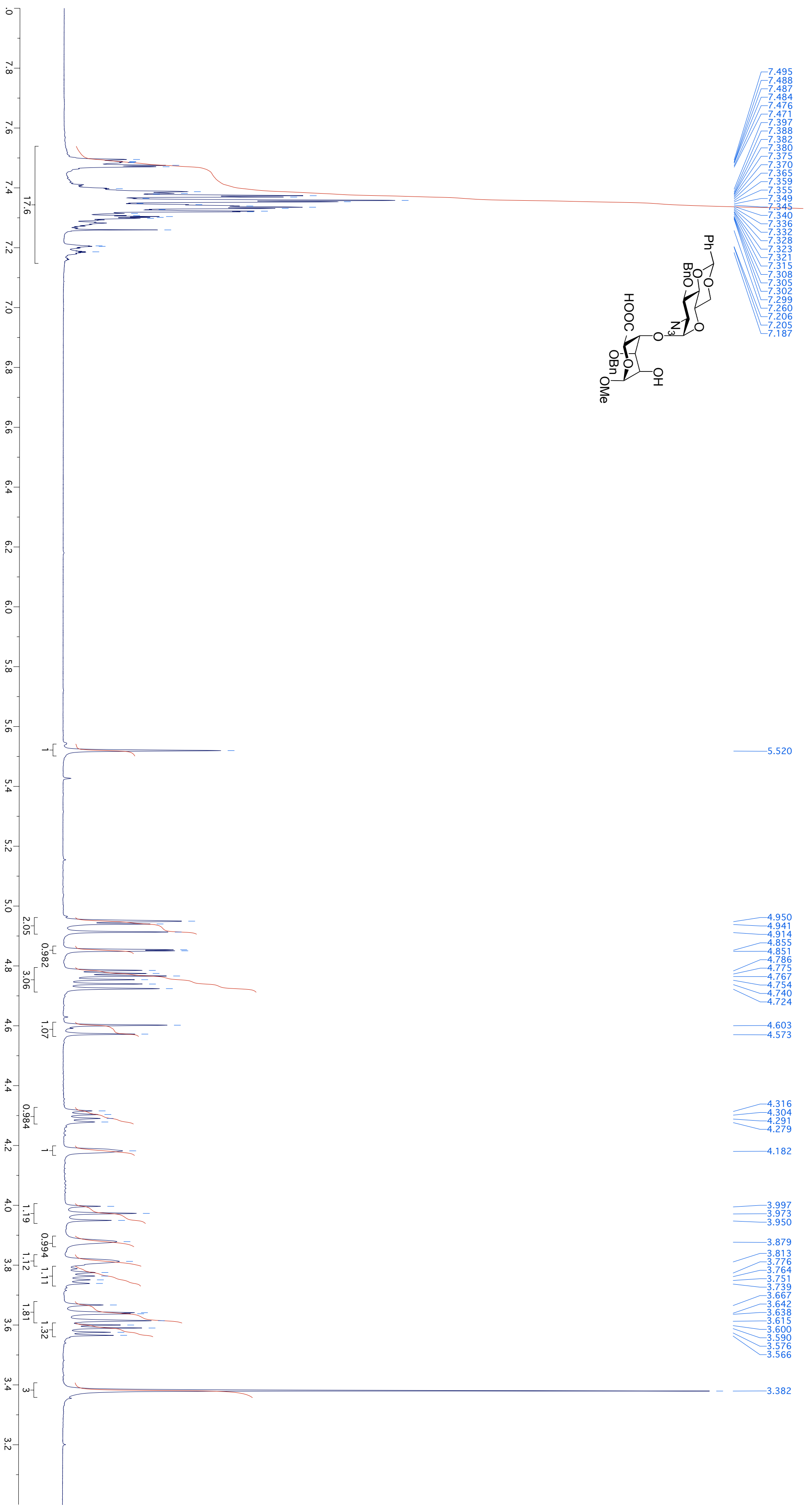

2-azido-3-*O*-benzyl-4,6-*O*-benzylidene-2-deoxy-α-D-glucopyranosyl-(1⟶4)-(methyl 3-*O*-benzyl-β-L-idopyranoside) uronic acid 11

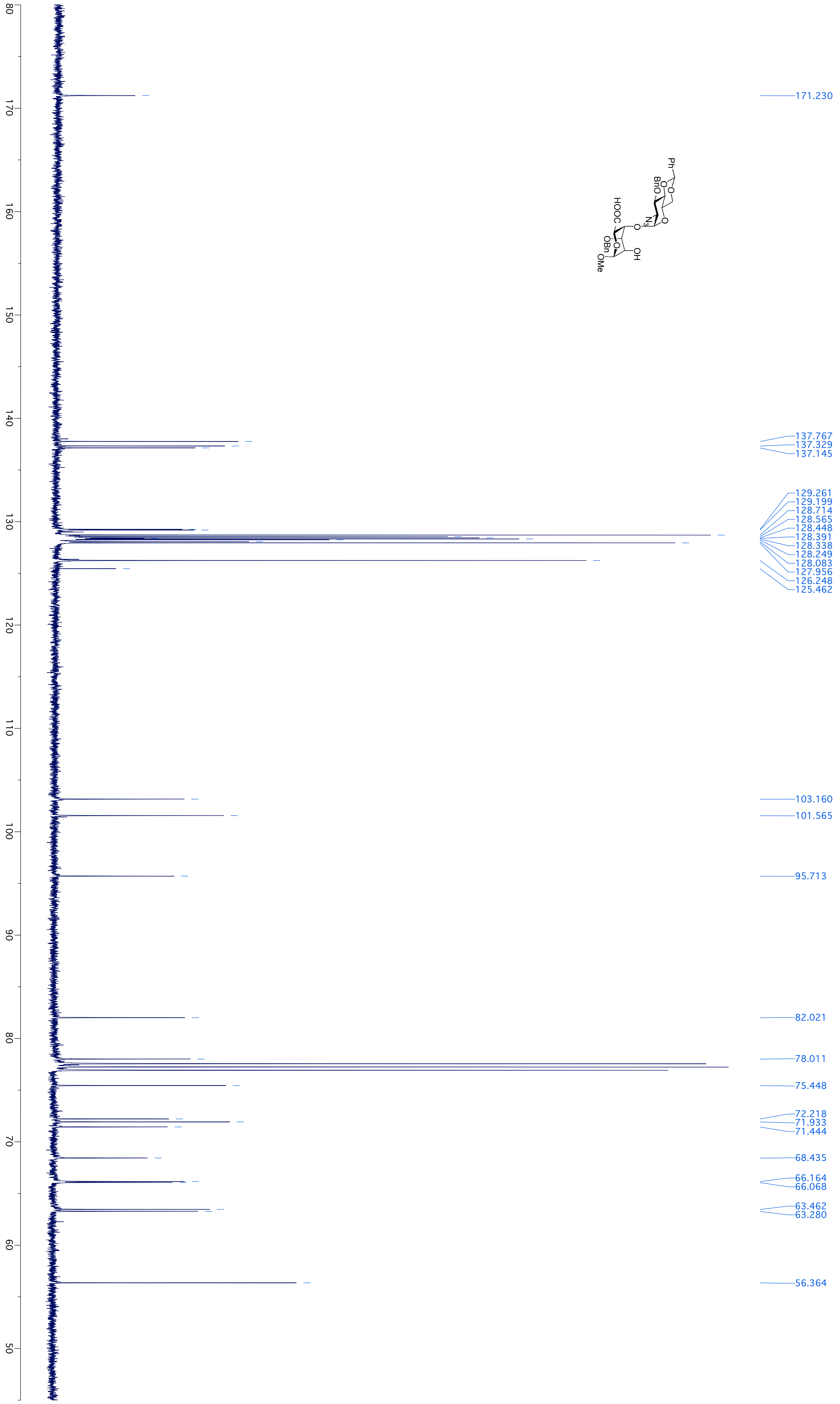

2-azido-3-*O*-benzyl-4,6-*O*-benzylidene-2-deoxy-α-D-glucopyranosyl-(1⟶4)-(methyl 3-*O*-benzyl-β-L-idopyranoside) uronic acid 11

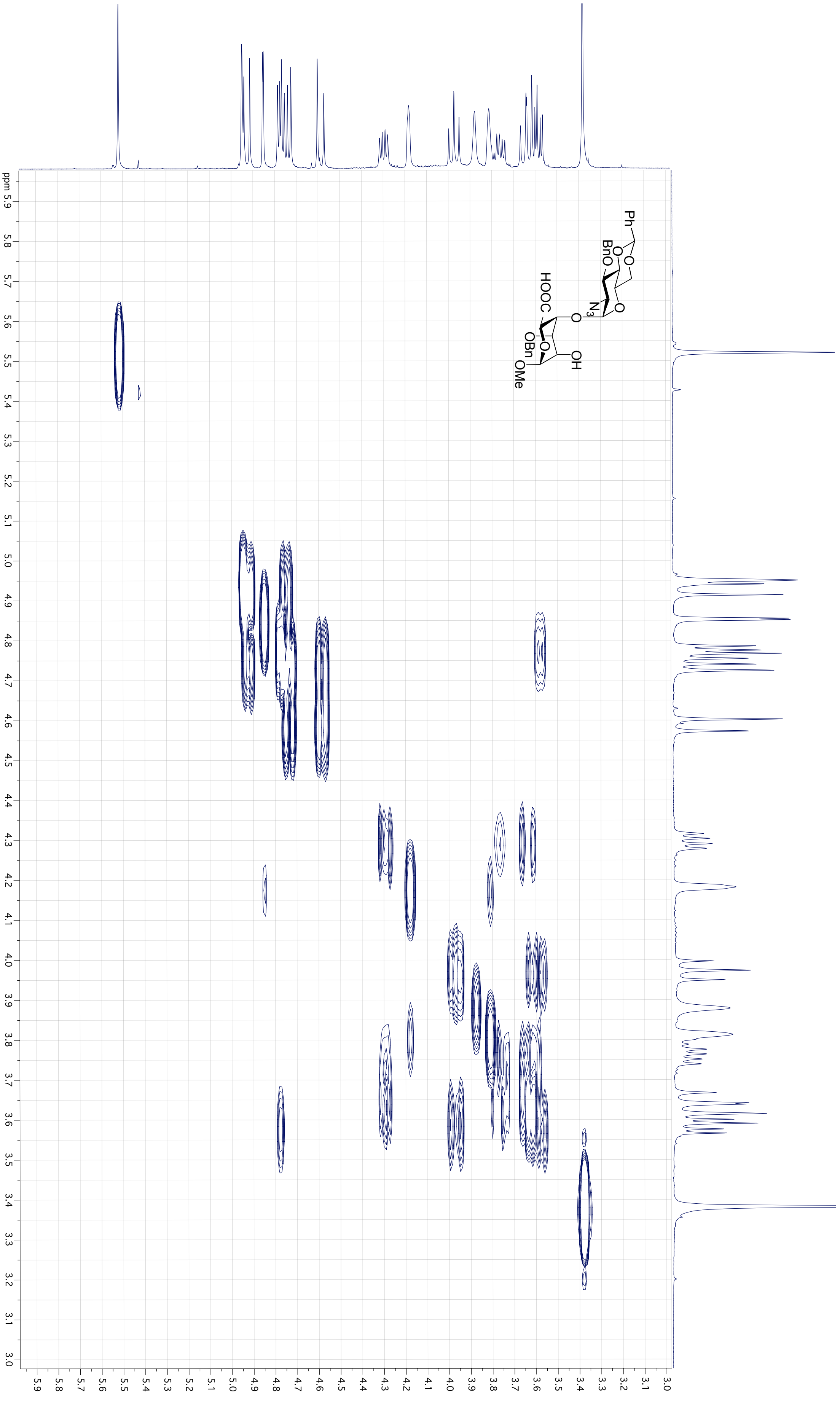

2-Azido-3-*O*-benzyl-2-deoxy-4-*O*-*p*-methoxybenzyl-6-*O*-sulfonato-α-D-glucopyranosyl-(1⟶4)-(methyl 3-*O*-benzyl-2-*O*-sulfonato-α-L-idopyranoside) uronic acid di sodium salt 12

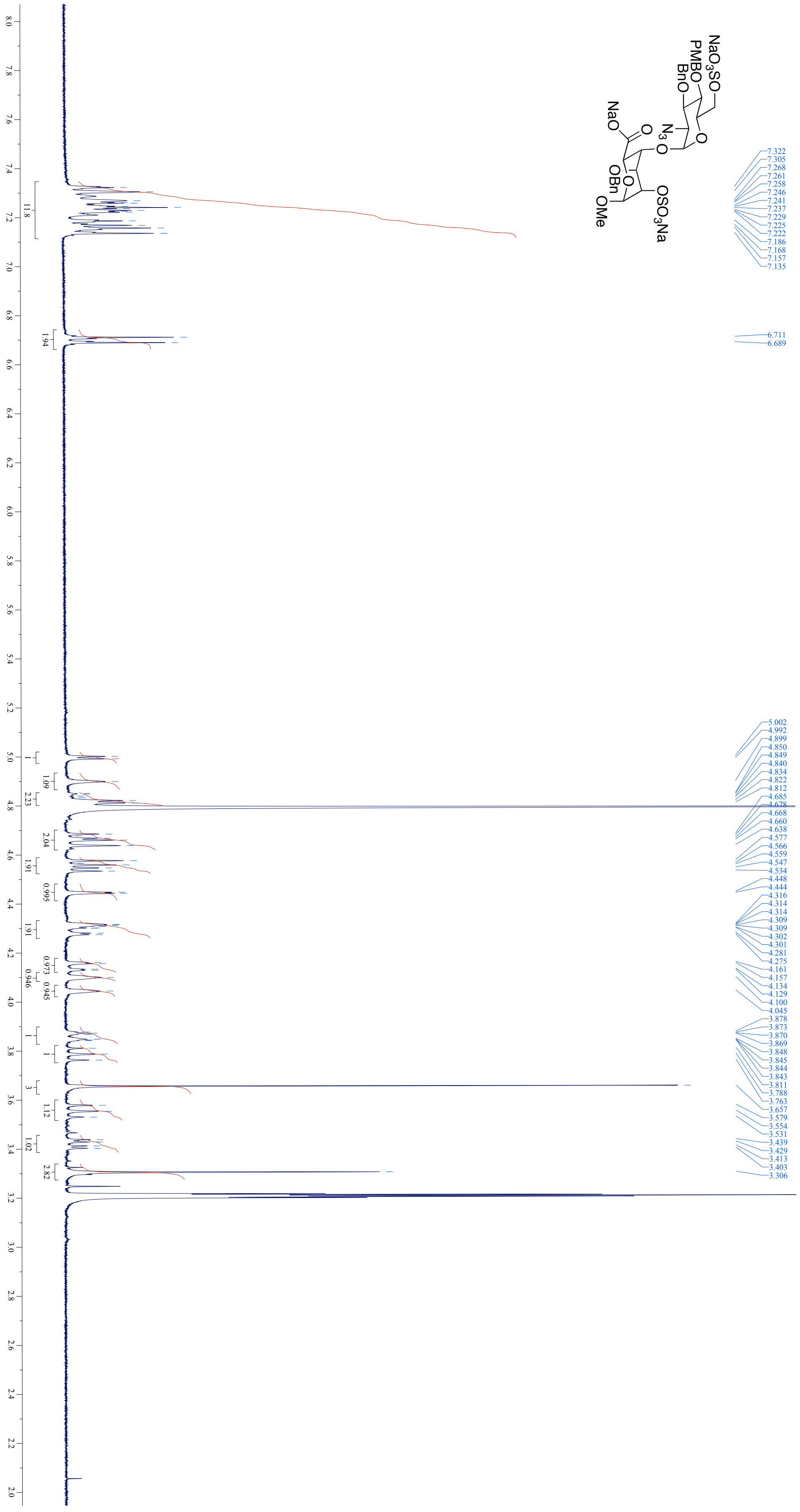

2-Azido-3-*O*-benzyl-2-deoxy-4-*O*-*p*-methoxybenzyl-6-*O*-sulfonato-α-D-glucopyranosyl-(1⟶4)-(methyl 3-*O*-benzyl-2-*O*-sulfonato-α-L-idopyranoside) uronic acid di sodium salt 12

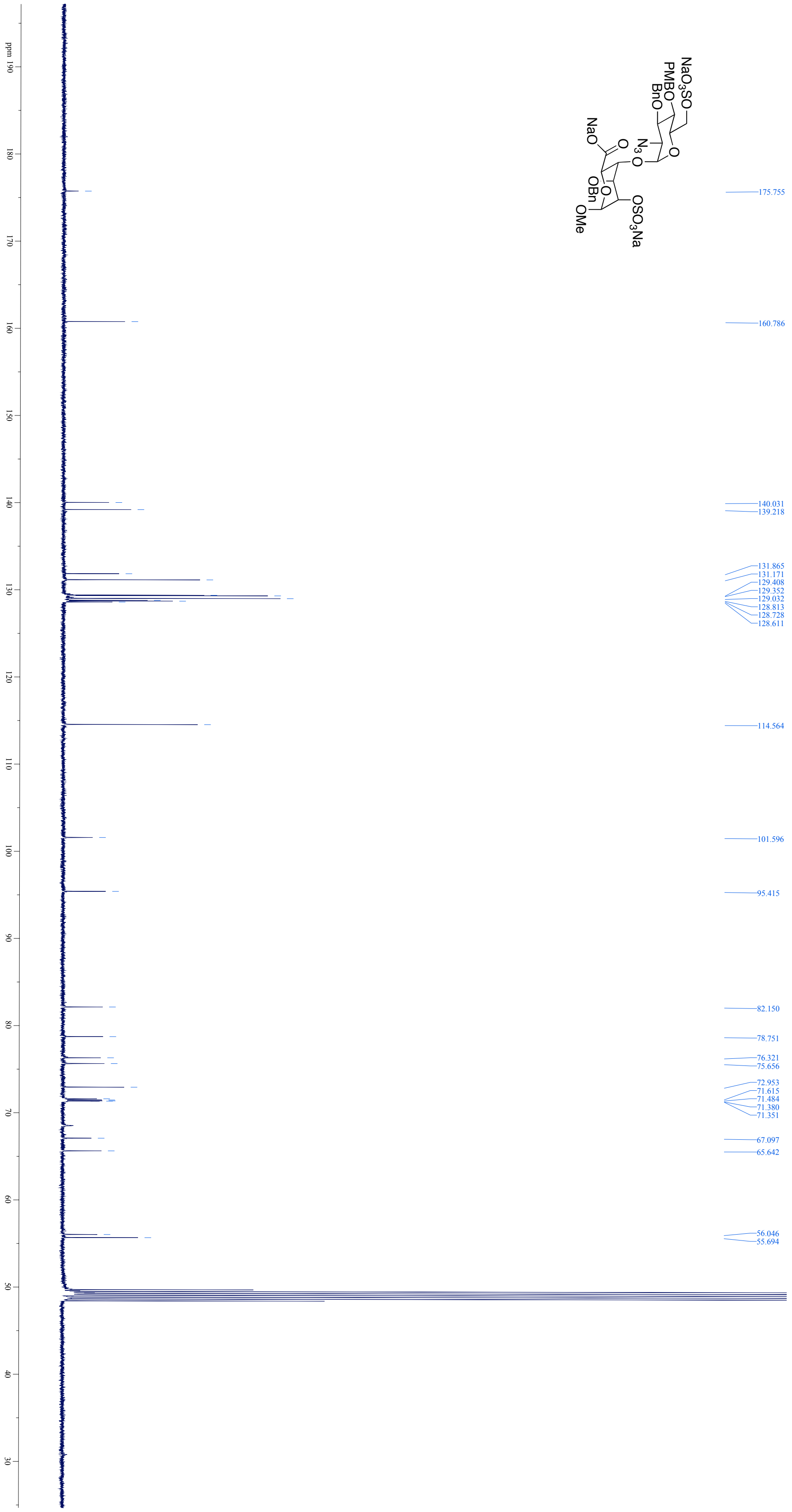

2-Azido-3-*O*-benzyl-2-deoxy-4-*O*-*p*-methoxybenzyl-6-*O*-sulfonato-α-D-glucopyranosyl-(1⟶4)-(methyl 3-*O*-benzyl-2-*O*-sulfonato-α-L-idopyranoside) uronic acid di sodium salt 12

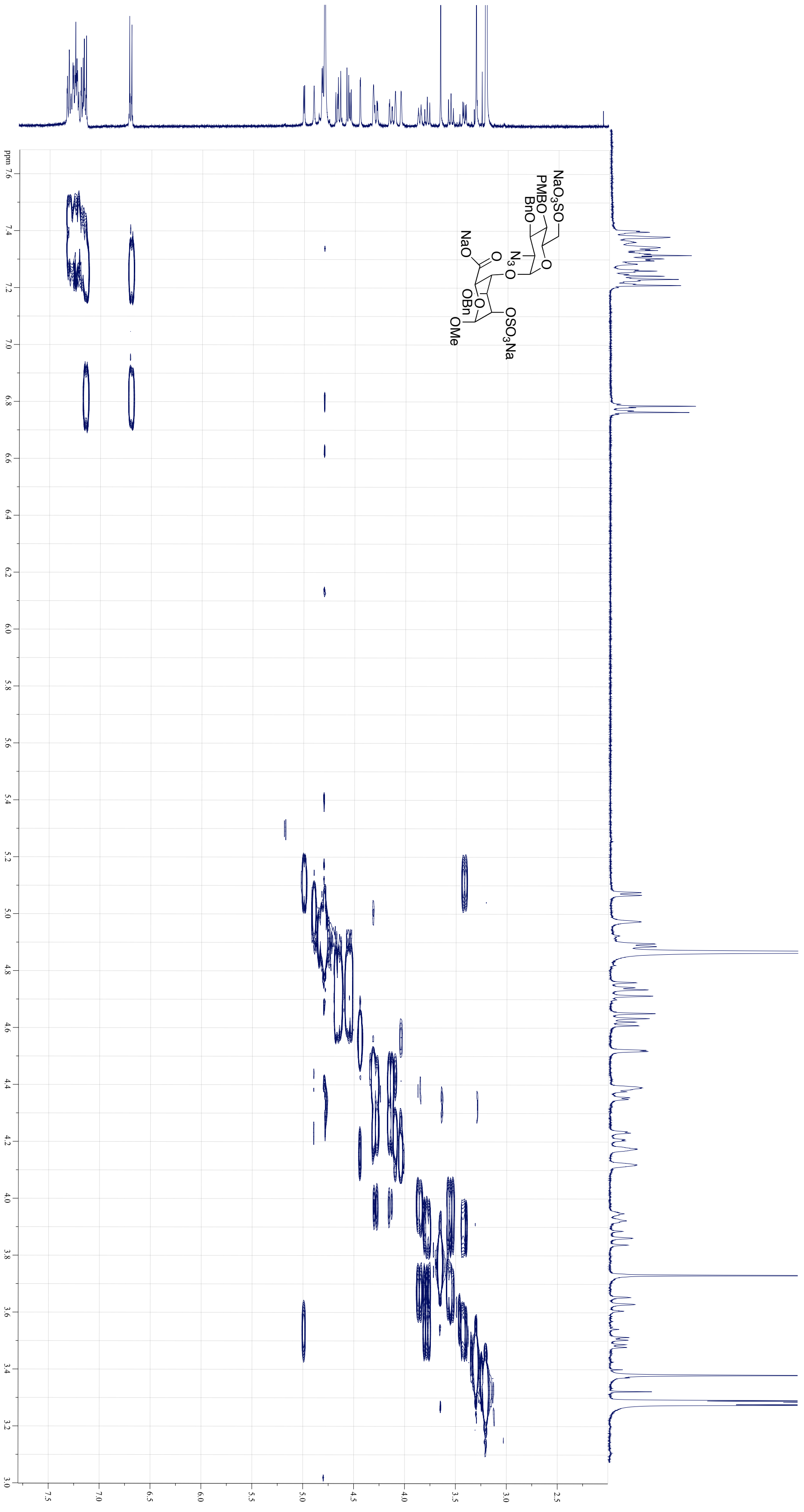

2-azido-3,6-di-*O*-benzyl-2-deoxy-4-*O*-*p*-methoxybenzyl-α-D-glucopyranosyl-(1⟶4)-(methyl 2-*O*-sulfonato-3-*O*-benzyl-α-L-idopyranoside) uronic acid mono sodium salt 13

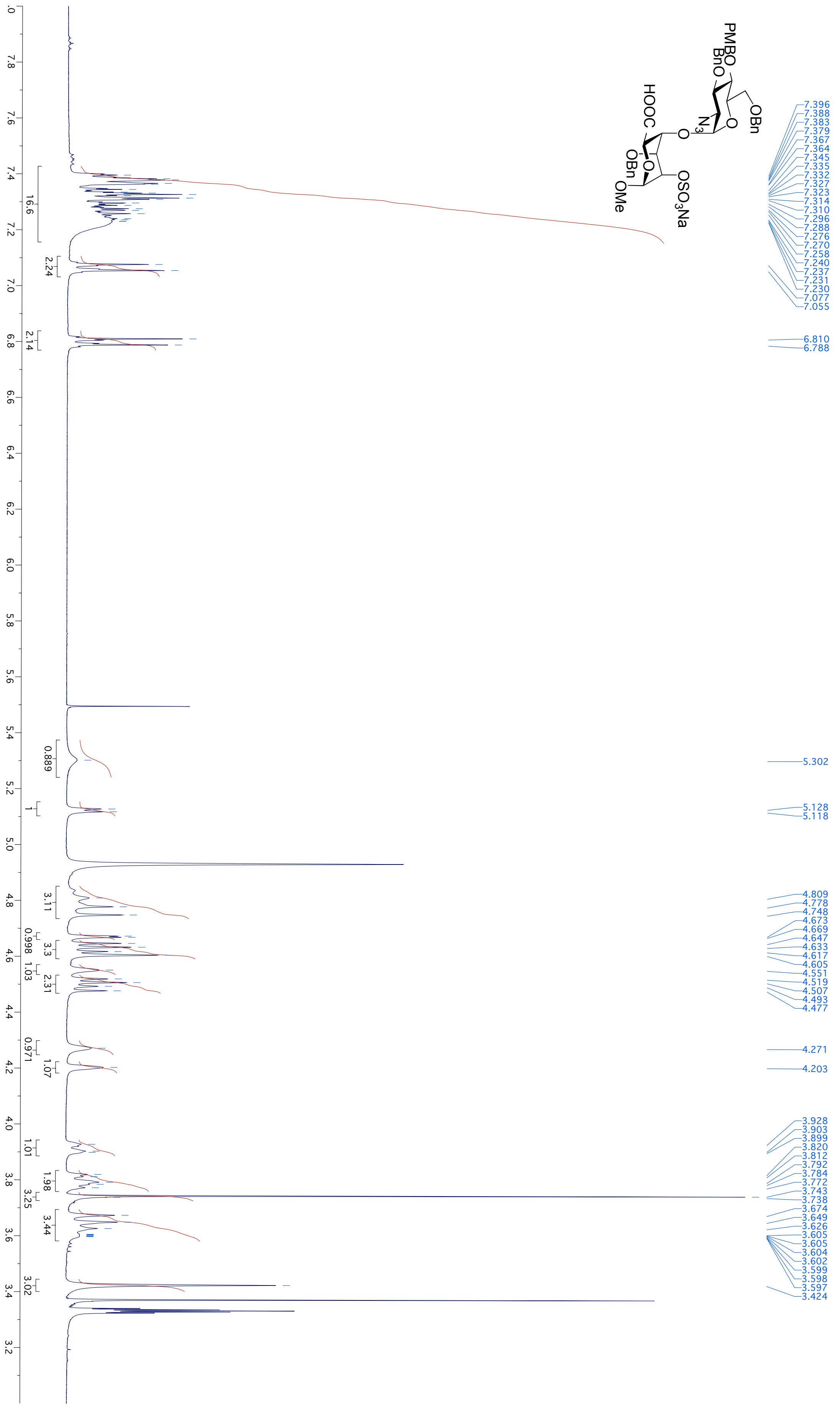

2-azido-3,6-di-*O*-benzyl-2-deoxy-4-*O*-*p*-methoxybenzyl-α-D-glucopyranosyl-(1⟶4)-(methyl 2-*O*-sulfonato-3-*O*-benzyl-α-L-idopyranoside) uronic acid mono sodium salt 13

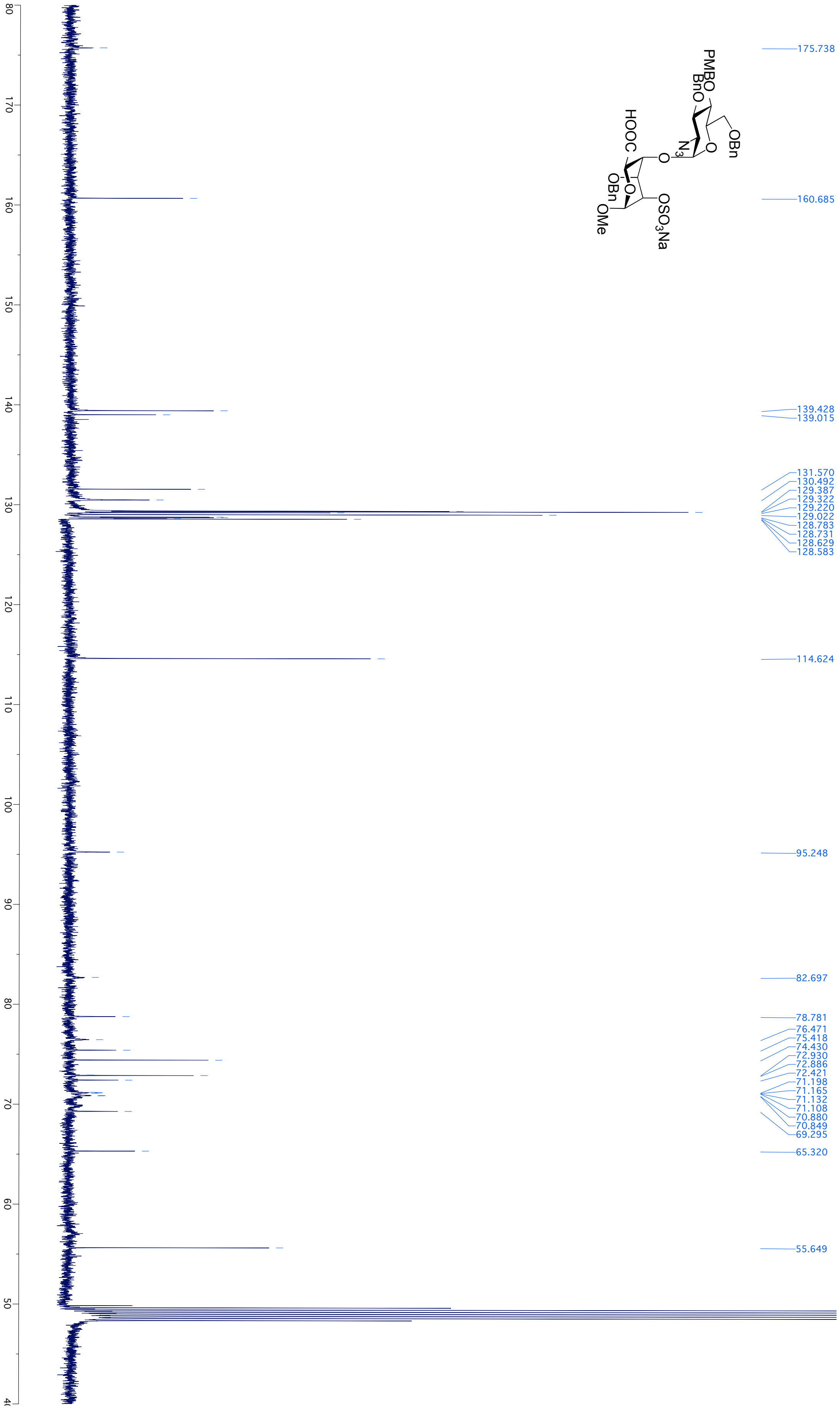

2-azido-3,6-di-*O*-benzyl-2-deoxy-4-*O*-*p*-methoxybenzyl-α-D-glucopyranosyl-(1⟶4)-(methyl 2-*O*-sulfonato-3-*O*-benzyl-α-L-idopyranoside) uronic acid mono sodium salt 13

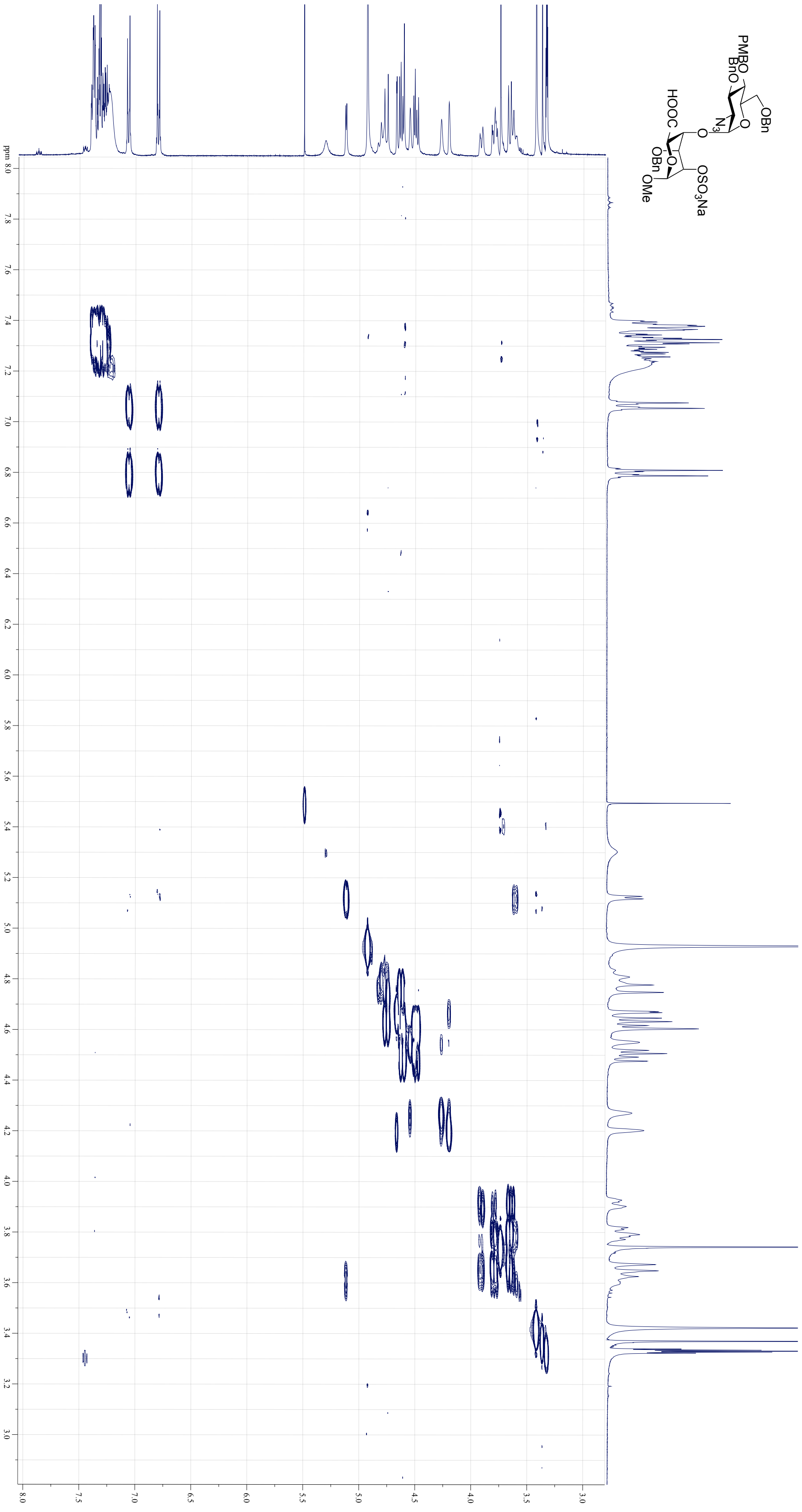

Methyl-6-O-sulfonato-α-D-glucosaminopyranosyl-(1⟶4)-2-O-sulfonato-α-L-idopyranoside uronate tri sodium salt 14

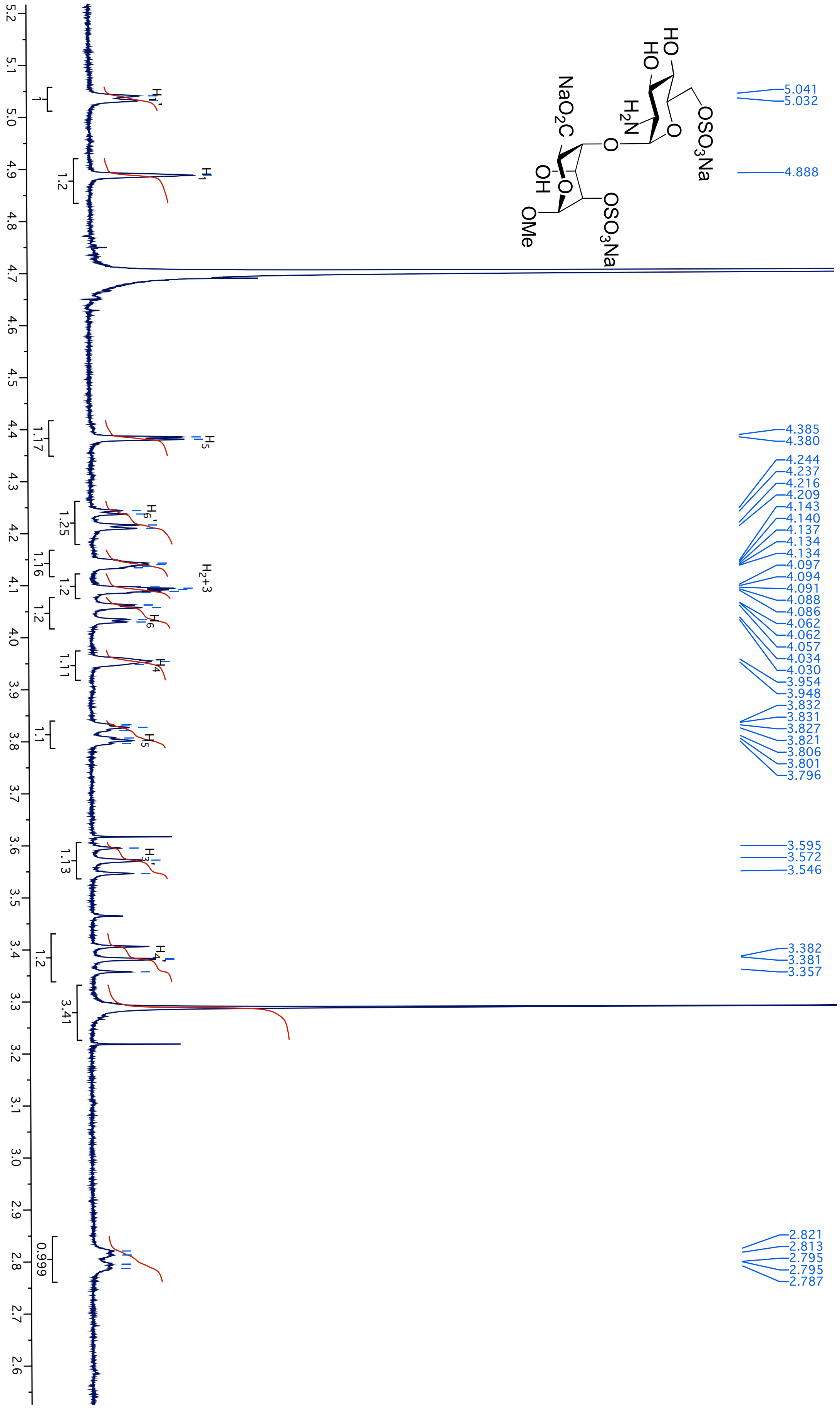

Methyl-6-O-sulfonato-α-D-glucosaminopyranosyl-(1⟶4)-2-O-sulfonato-α-L-idopyranoside uronate tri sodium salt 14

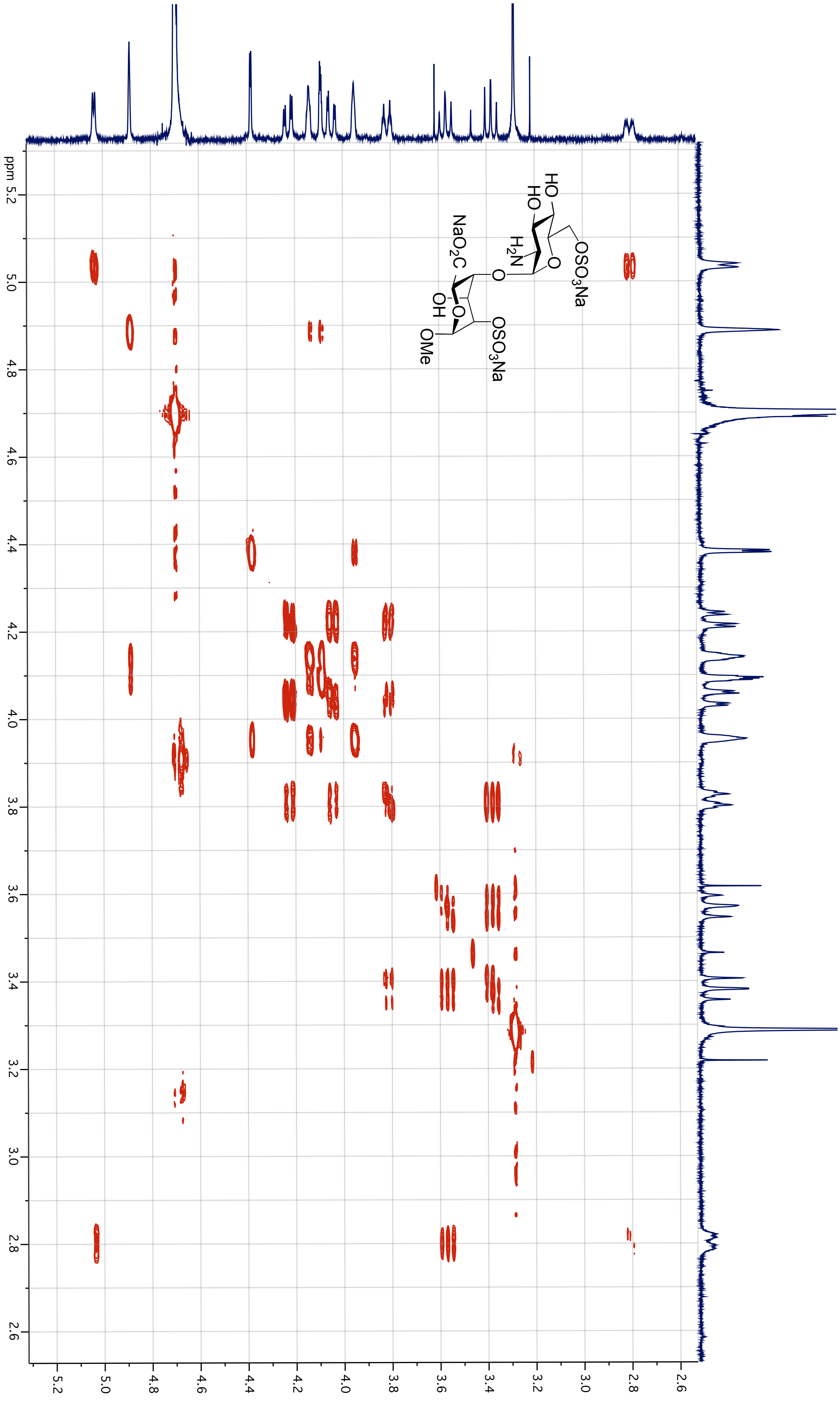

Methyl-α-D-glucosaminopyranosyl-(1⟶4)-2-O-sulfonato-α-L-idopyranoside uronate di sodium salt 15

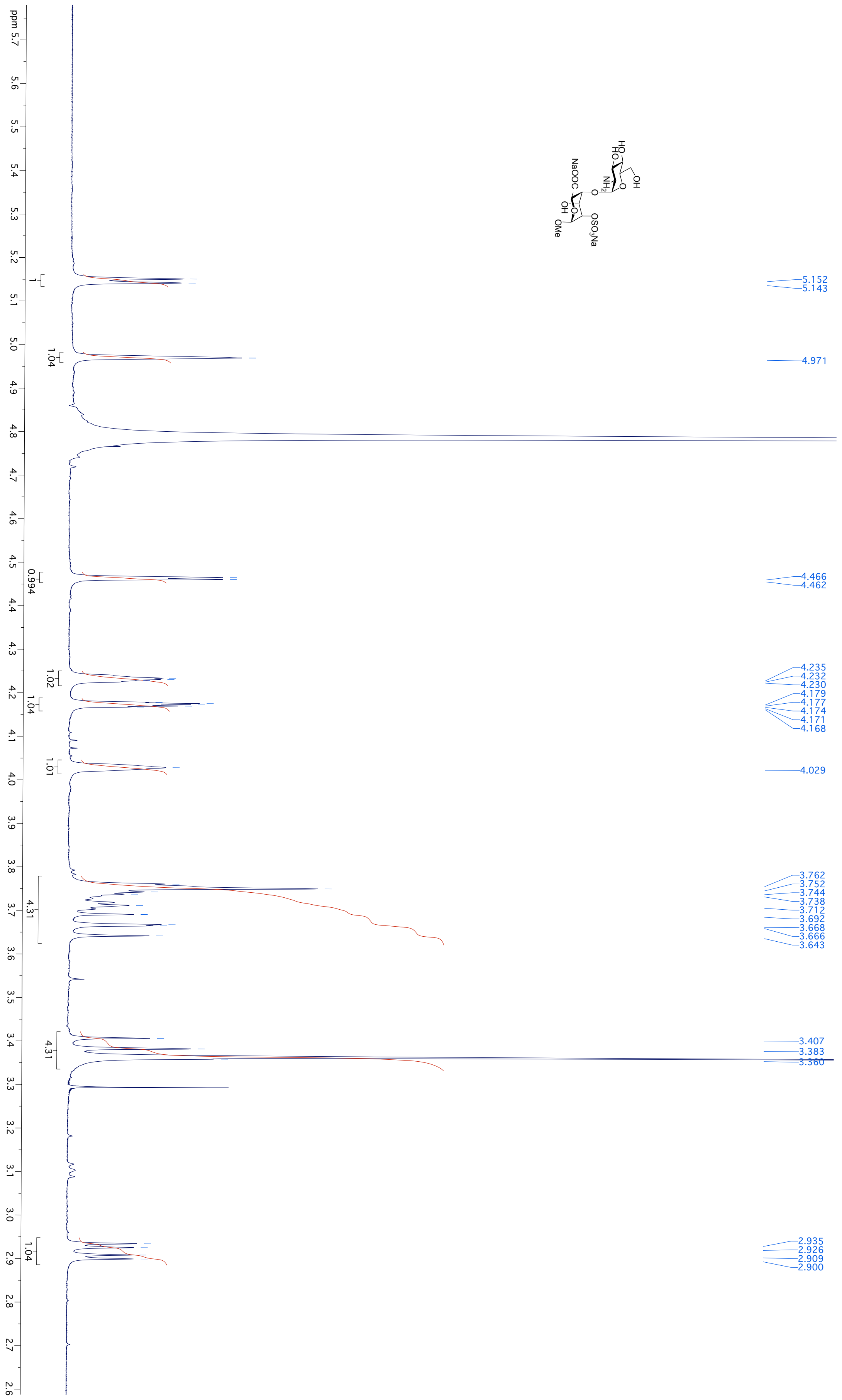

Methyl-α-D-glucosaminopyranosyl-(1⟶4)-2-O-sulfonato-α-L-idopyranoside uronate di sodium salt 15

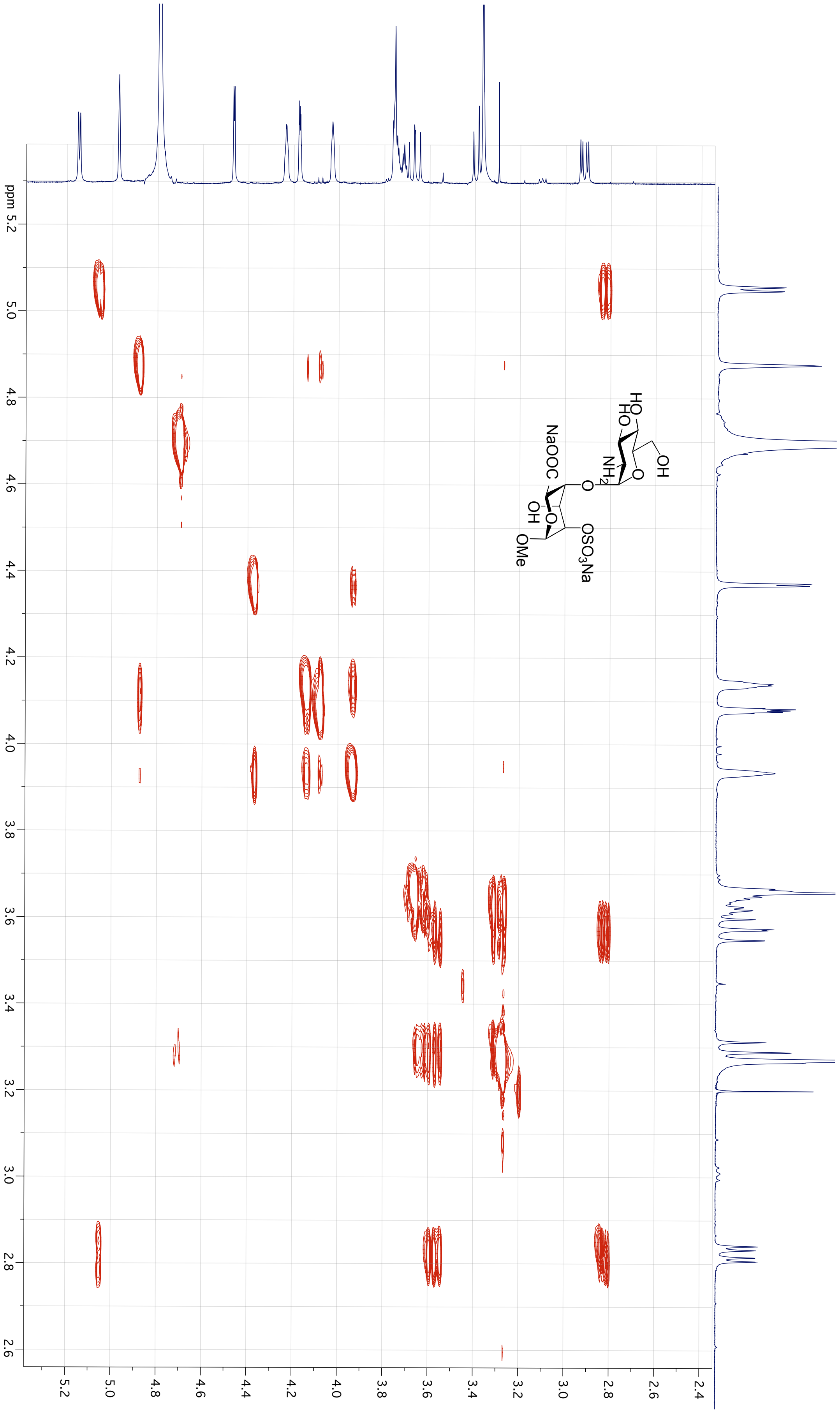

Methyl-α-D-glucosaminopyranosyl-(1⟶4)-2-O-sulfonato-α-L-idopyranoside uronate di sodium salt 15

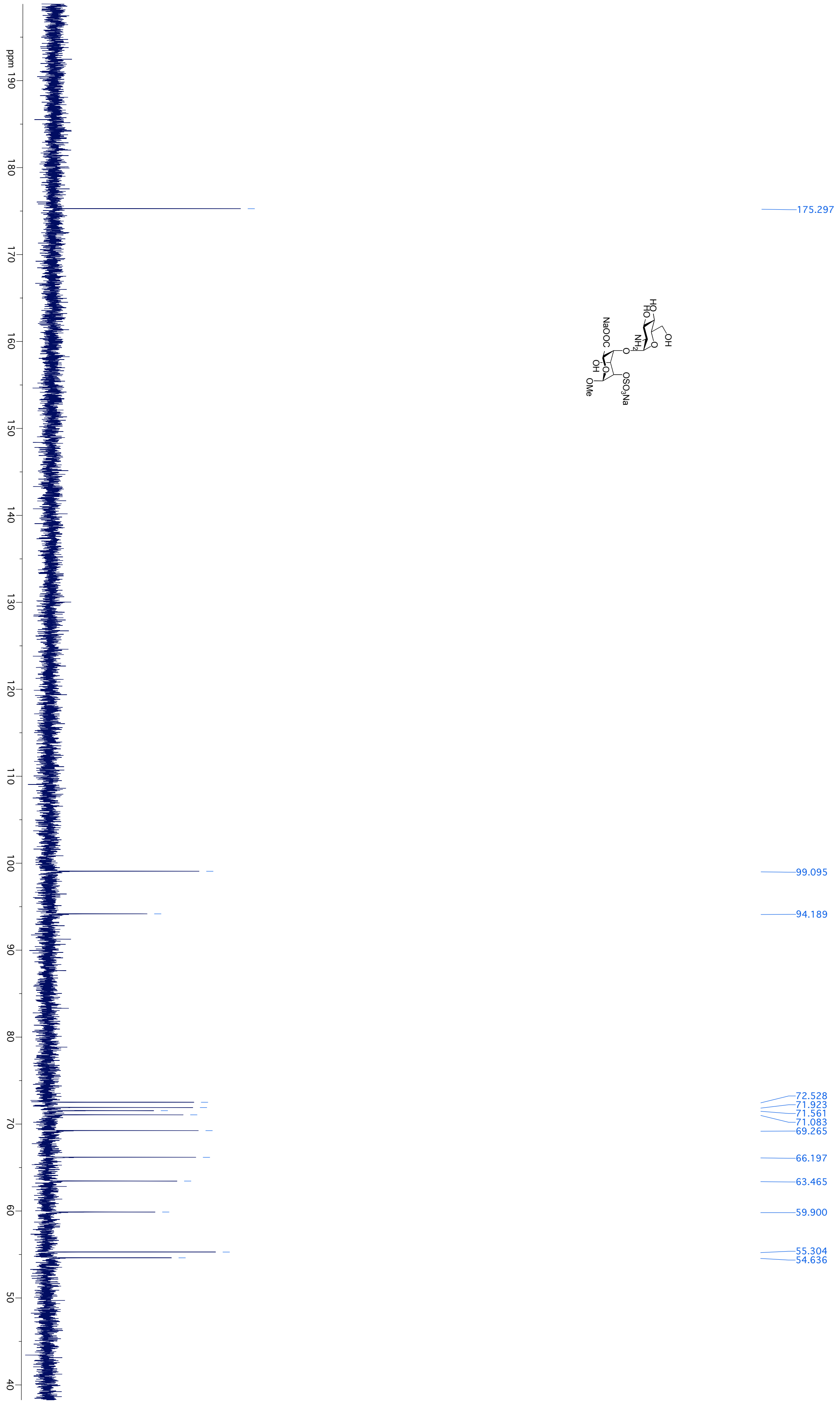

Methyl-6-O, N-di-sulfonato-α-D-glucosaminopyranosyl-(1⟶4)-2-O-sulfonato-α-L-idopyranoside uronate tetra sodium salt 16

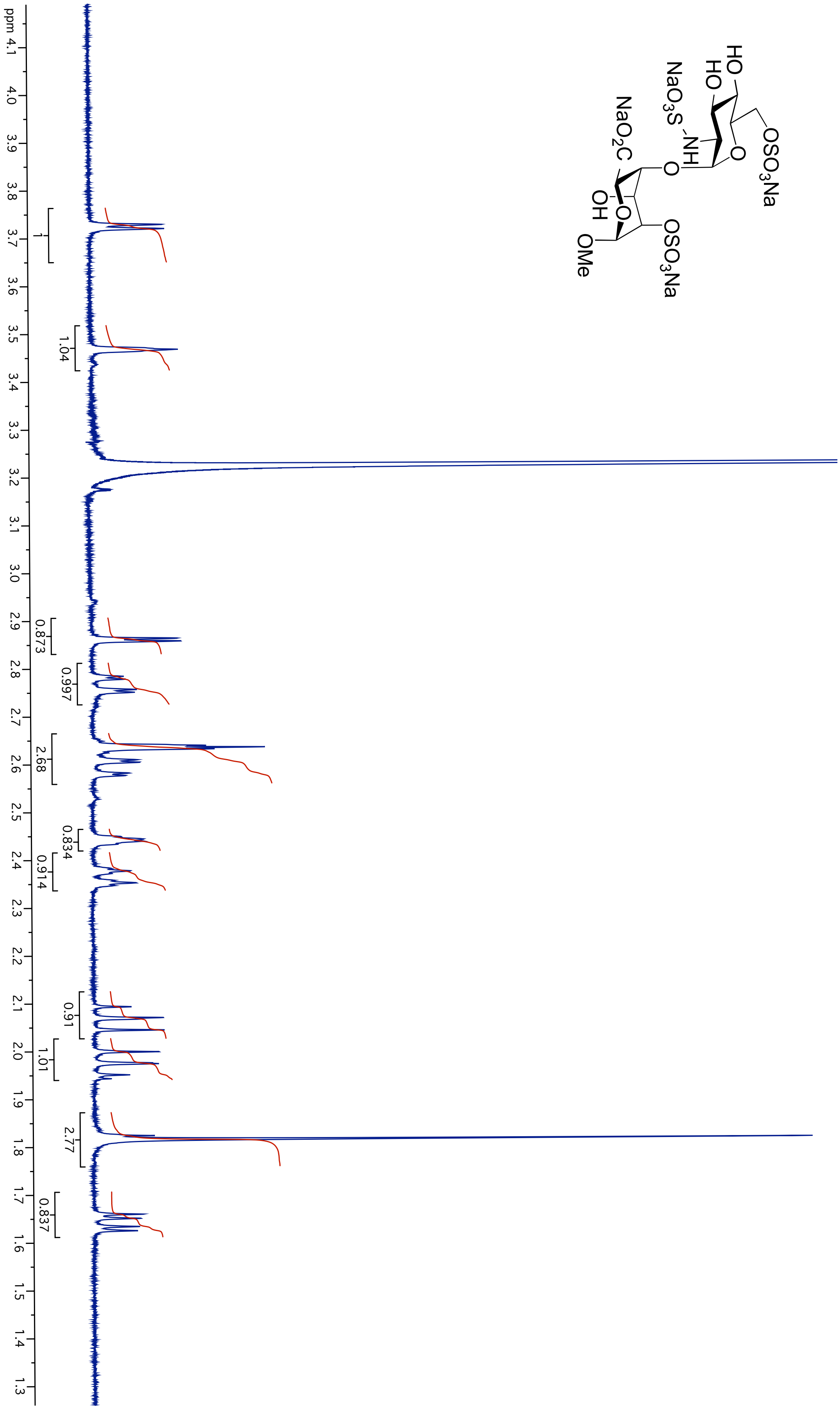

Methyl-6-O, N-di-sulfonato-α-D-glucosaminopyranosyl-(1⟶4)-2-O-sulfonato-α-L-idopyranoside uronate tetra sodium salt 16

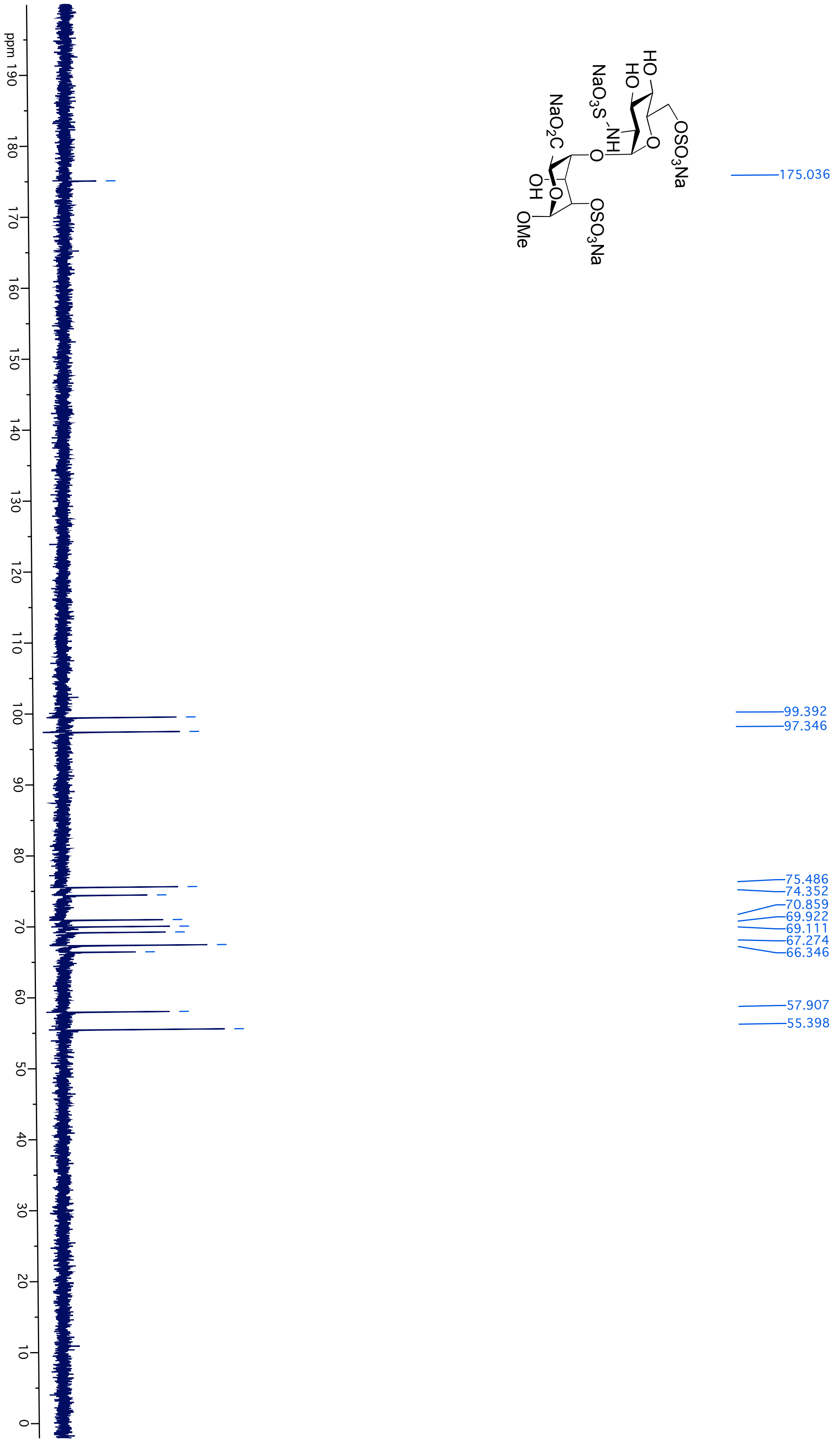

Methyl-6-O, N-di-sulfonato-α-D-glucosaminopyranosyl-(1⟶4)-2-O-sulfonato-α-L-idopyranoside uronate tetra sodium salt 16

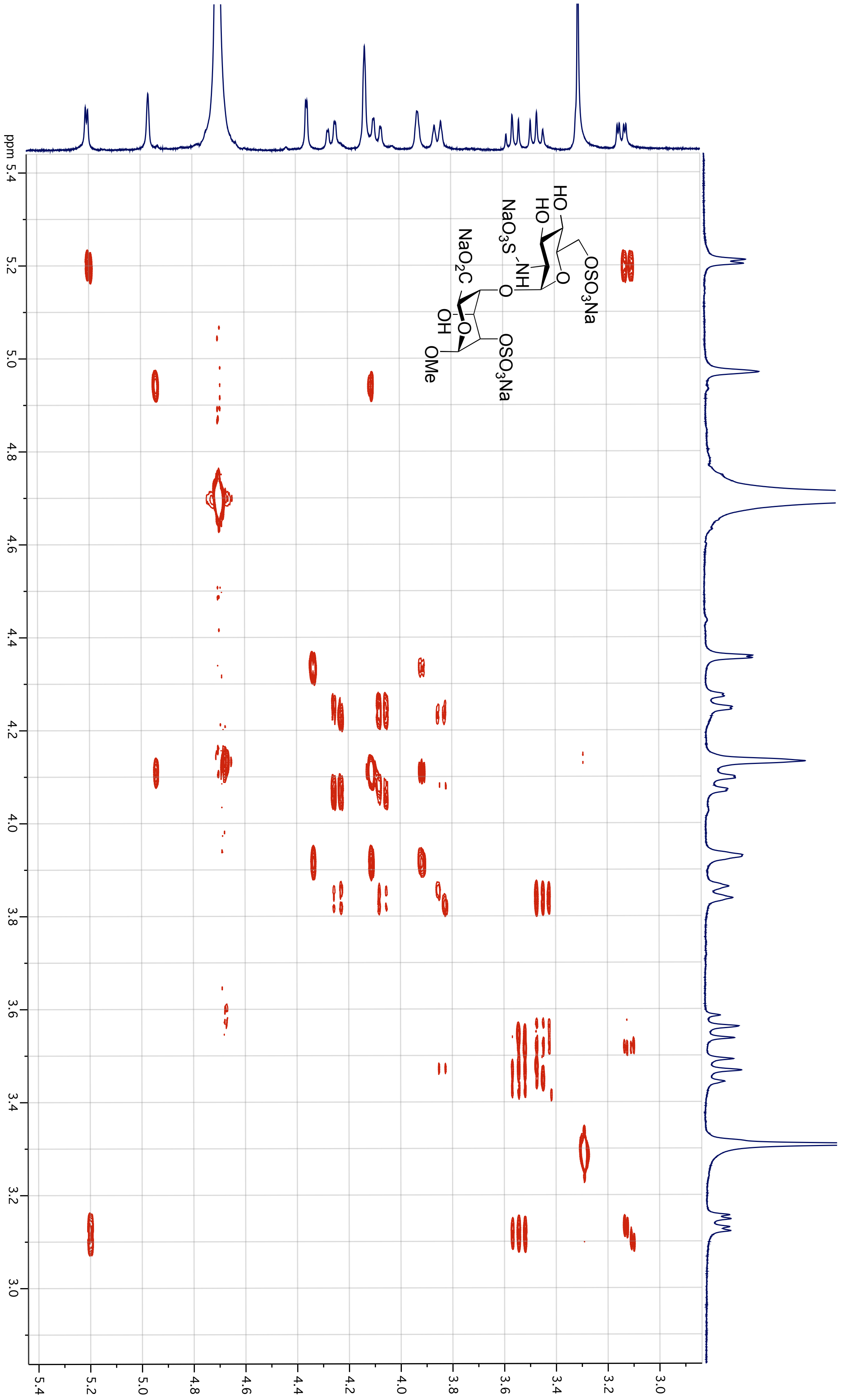

Methyl-N-sulfonato-α-D-glucosaminopyranosyl-(1⟶4)-2-O-sulfonato-α-L-idopyranoside uronate tri sodium salt 17

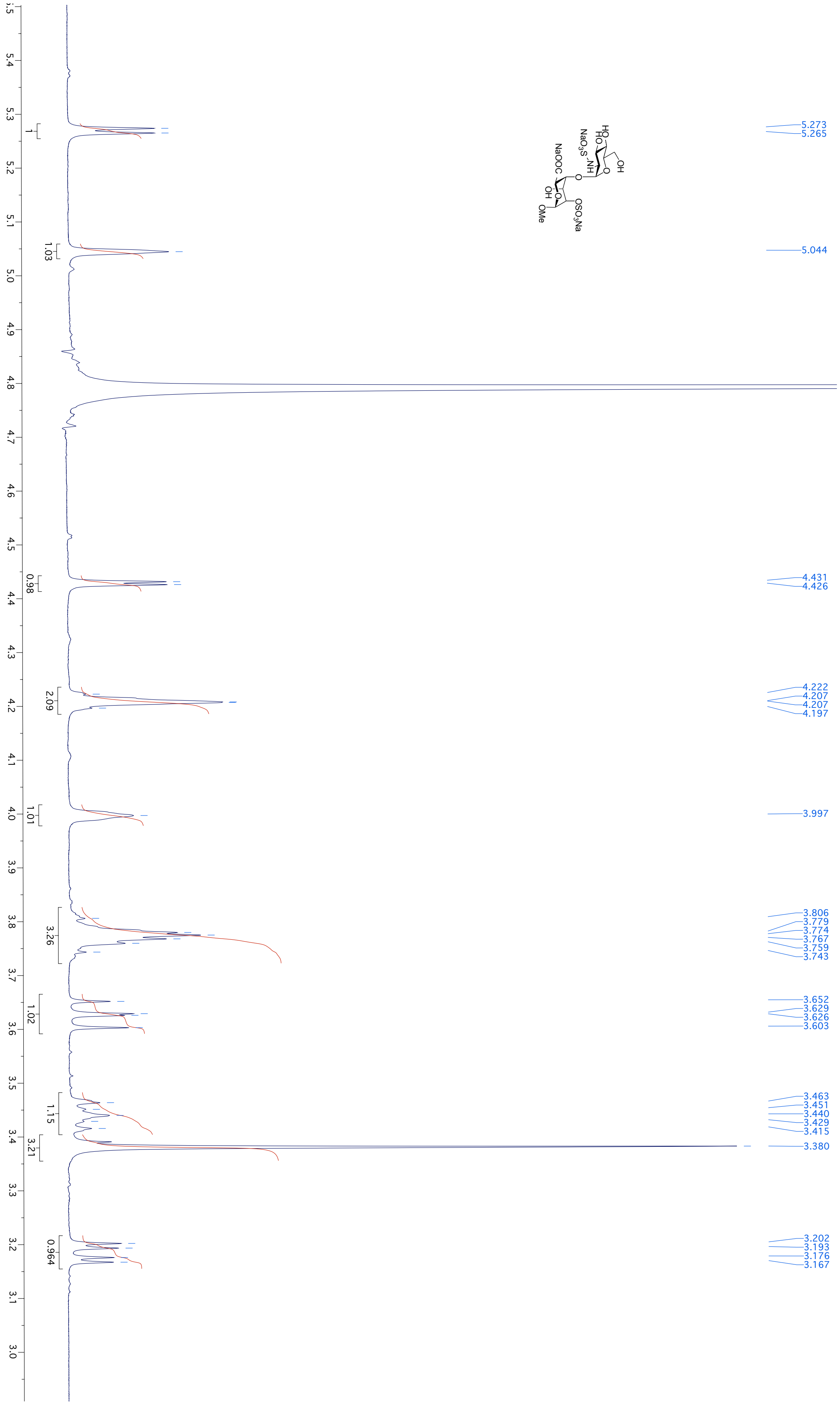

Methyl-N-sulfonato-α-D-glucosaminopyranosyl-(1⟶4)-2-O-sulfonato-α-L-idopyranoside uronate tri sodium salt 17

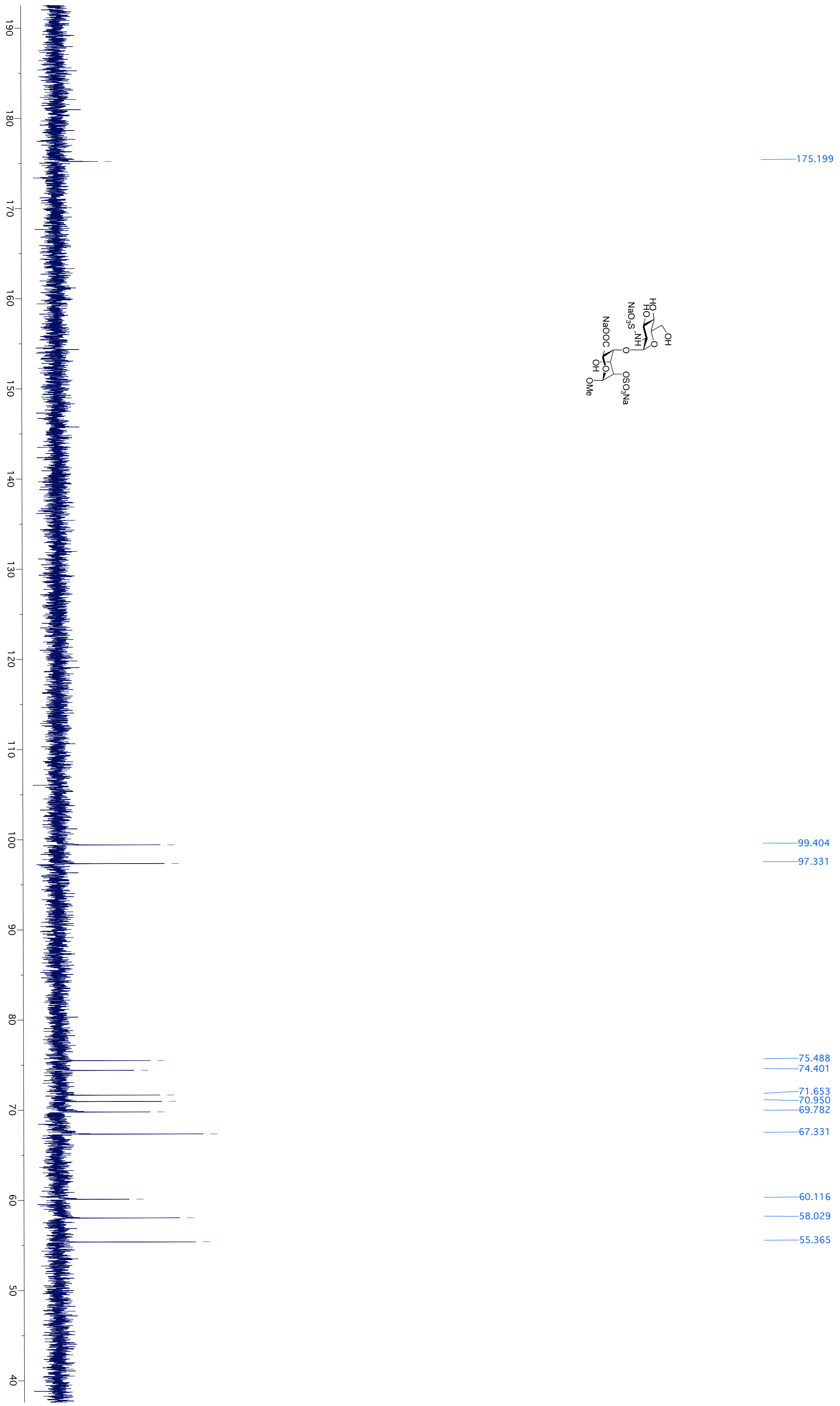

Methyl-N-sulfonato-α-D-glucosaminopyranosyl-(1⟶4)-2-O-sulfonato-α-L-idopyranoside uronate tri sodium salt 17

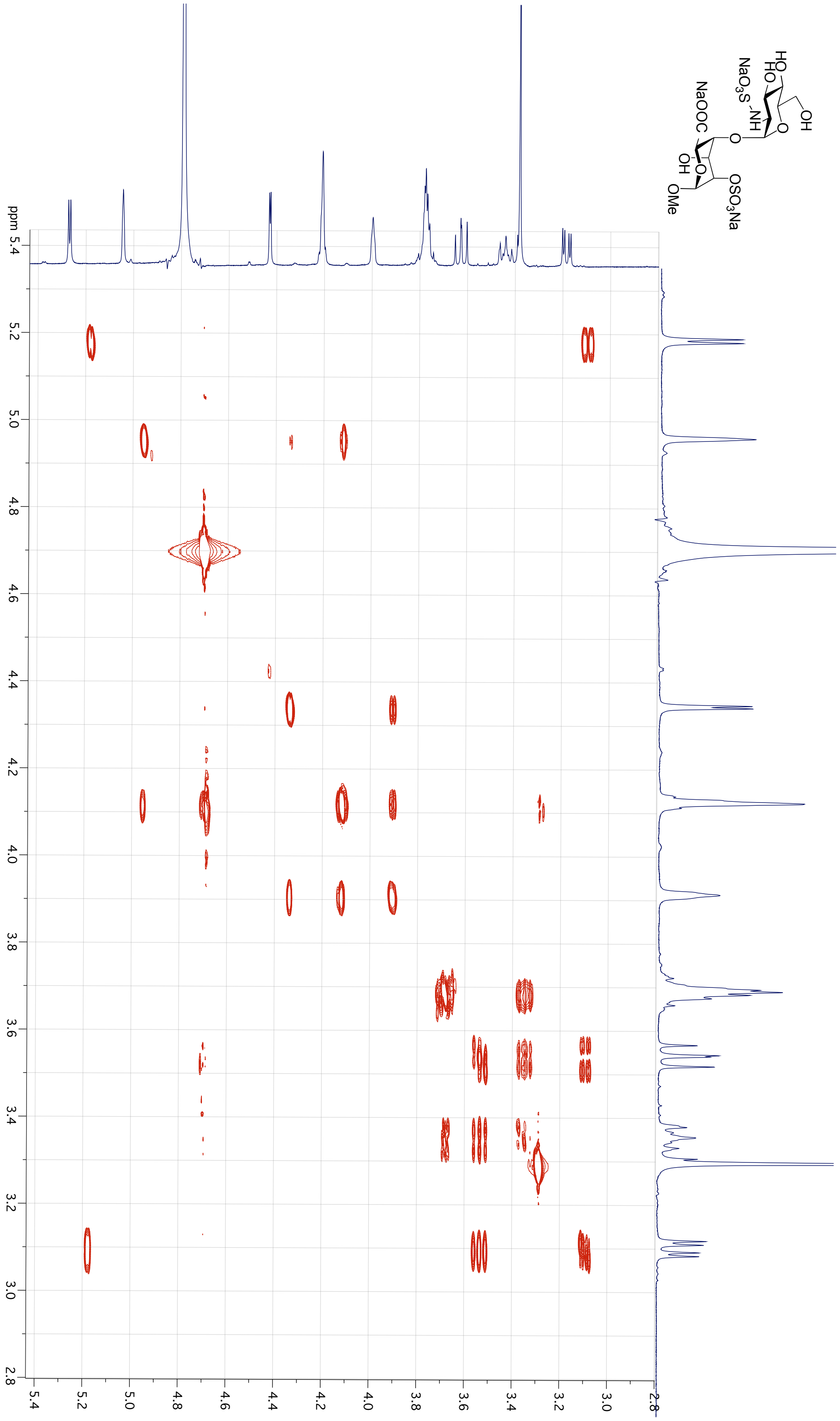

Methyl-α-D-glucosaminopyranosyl-(1⟶4)-α-L-idopyranoside uronate mono sodium salt 18

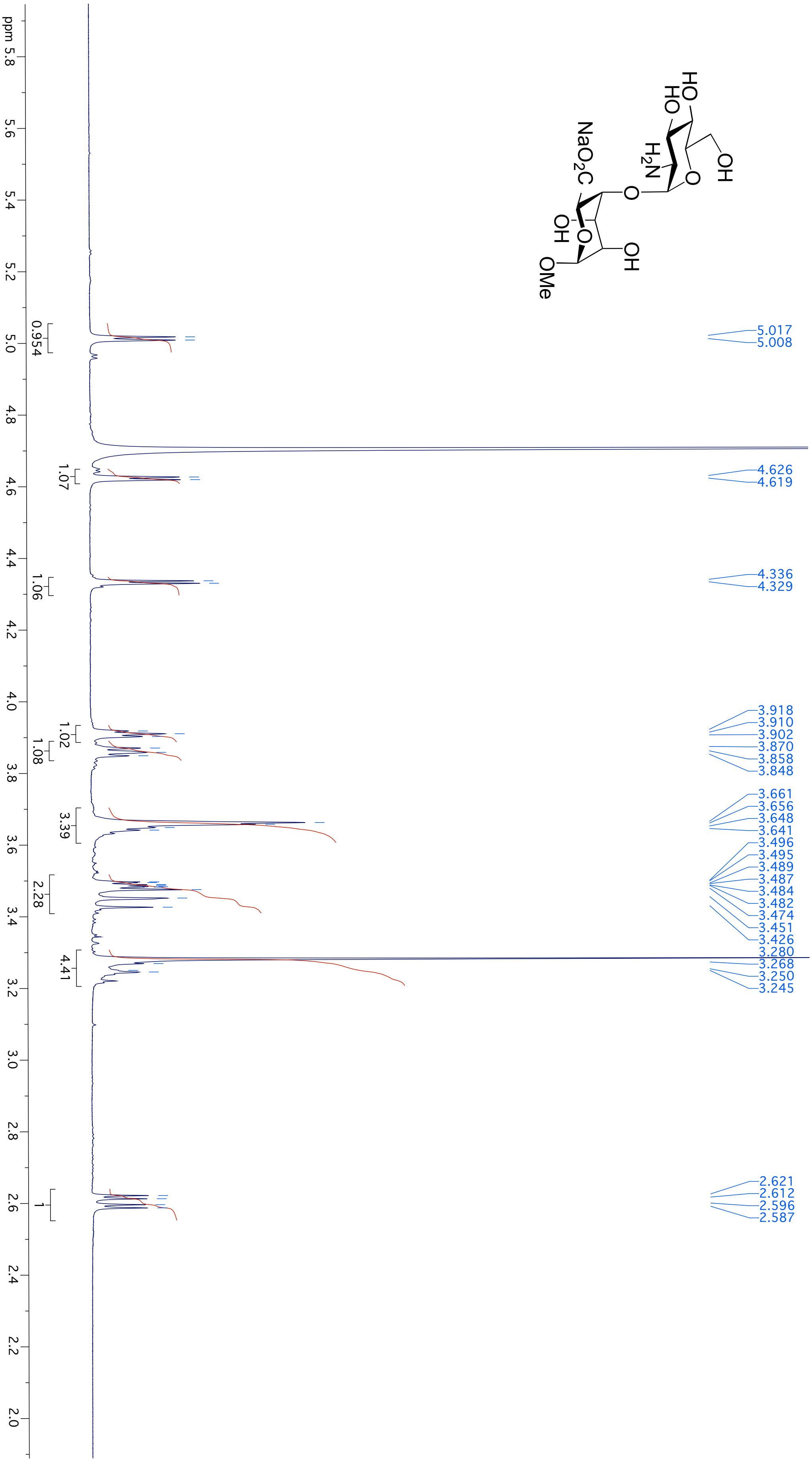

Methyl-α-D-glucosaminopyranosyl-(1⟶4)-α-L-idopyranoside uronate mono sodium salt 18

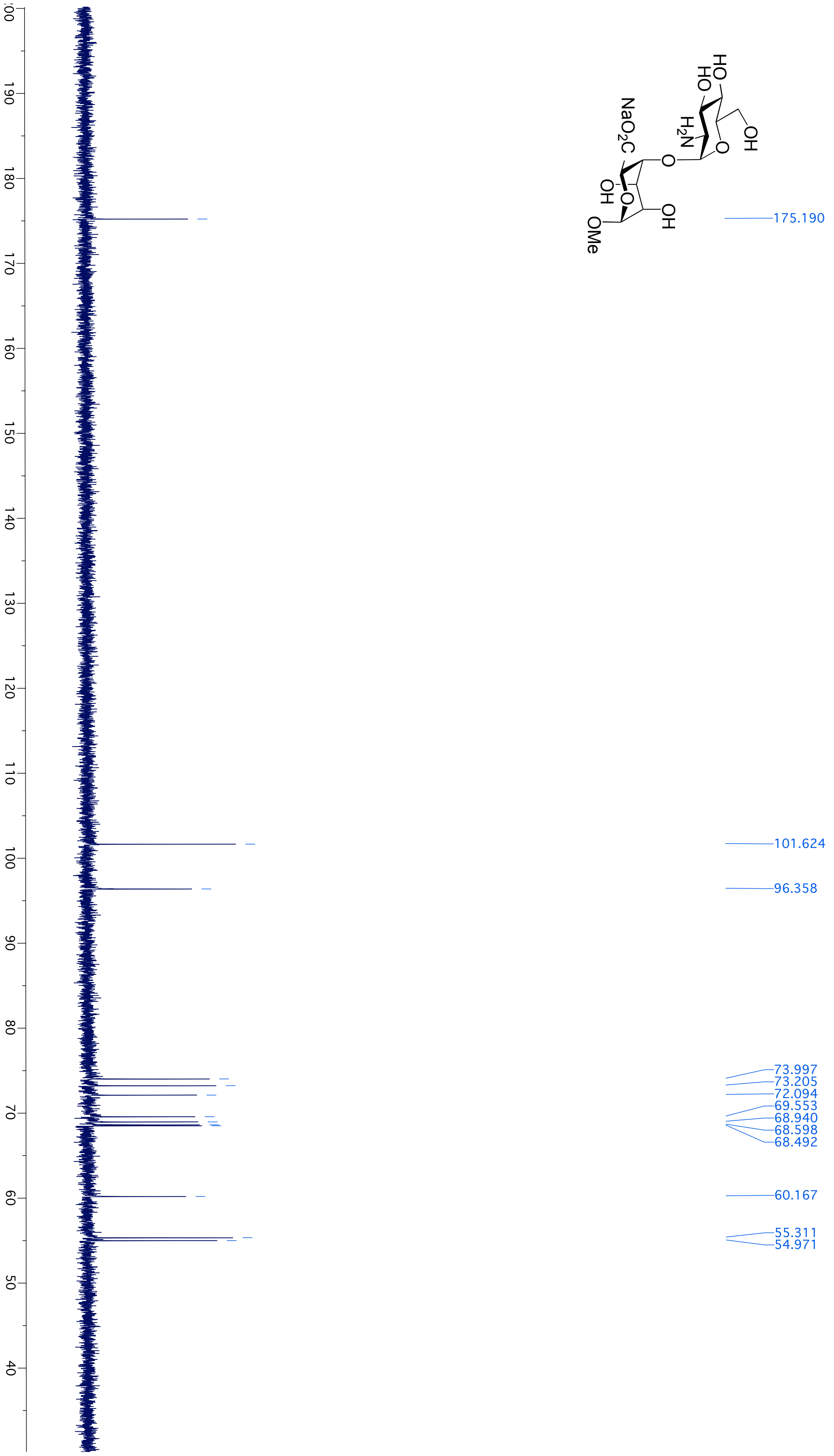

Methyl-α-D-glucosaminopyranosyl-(1⟶4)-α-L-idopyranoside uronate mono sodium salt 18

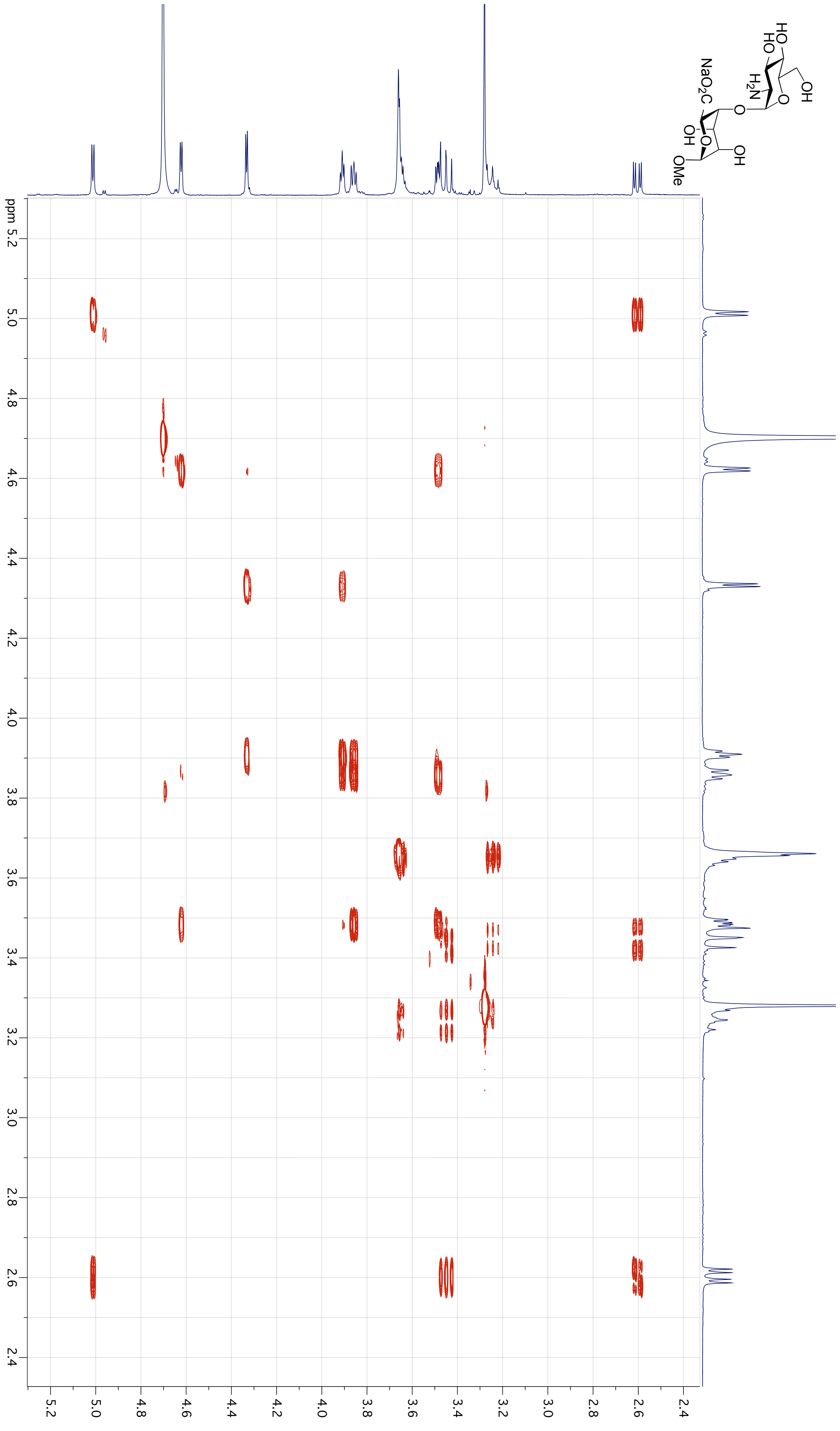

Supplement: Supplementary data 1 — Supplementary material. [file mmc1.pdf]
